# Structure-Based Design of PROTACS for the Degradation of Soluble Epoxide Hydrolase

**DOI:** 10.1021/acs.jmedchem.5c00552

**Published:** 2025-06-18

**Authors:** Julia Schönfeld, Steffen Brunst, Ludmila Ciomirtan, Lena Willmer, Michel A. Chromik, Adarsh Kumar, Timo Froemel, Nick Liebisch, Arne Hackspacher, Johanna H. M. Ehrler, Lukas Wintermeier, Christina Hesse, Jan Fiedler, Jan Heering, Hinrich Freitag, Patrick Zardo, Hans-Gerd Fieguth, Astrid Brüggerhoff, Josefine Jakob, Björn Häupl, Lilia Weizel, Astrid Kaiser, Manfred Schubert-Zsilavecz, Thomas Oellerich, Ingrid Fleming, Nils H. Schebb, Robert Fürst, Aimo Kannt, Stefan Knapp, Ewgenij Proschak, Kerstin Hiesinger

**Affiliations:** † Institute of Pharmaceutical Chemistry, 9173 Goethe University, 60438 Frankfurt am Main, Germany; ‡ Institute of Pharmaceutical Biology, 9173 Goethe University, 60438 Frankfurt am Main, Germany; § 14950 Fraunhofer Institute for Toxicology and Experimental Medicine ITEM, Member of Fraunhofer Cluster Immune Mediated Diseases (CIMD), 60596 Frankfurt am Main, Germany; ∥ Biomedical Research in Endstage and Obstructive Lung Disease Hannover (BREATH), Member of the German Center for Lung Research (DZL), 30625 Hannover, Germany; ⊥ Chair of Food Chemistry, Faculty of Mathematics and Natural Sciences, 26603 University of Wuppertal, 42119 Wuppertal, Germany; # Institute for Vascular Signalling, Centre for Molecular Medicine, 9173 Goethe University, 60596 Frankfurt am Main, Germany; ∇ Fraunhofer Institute for Translational Medicine and Pharmacology ITMP, 60596 Frankfurt am Main, Germany; ○ Institute of Pathology, Hannover Medical School, 30625 Hannover, Germany; ◆ Department of Cardiothoracic Transplantation and Vascular Surgery, 9177 Hannover Medical School, 30625 Hannover, Germany; ¶ KRH Clinics Hannover, 30459 Hannover, Germany; & Department of Medicine, Hematology and Oncology, University Hospital, Goethe University Frankfurt, 60596 Frankfurt am Main, Germany; ● Frankfurt Cancer Institute (FCI), 60596 Frankfurt am Main, Germany; ◊ German Cancer Consortium (DKTK), partner site Frankfurt/Mainz, a partnership between DKFZ and UCT Frankfurt-Marburg, Germany, 60590 Frankfurt am Main, Germany; ▲ German Cancer Research Center (DKFZ), 69120 Heidelberg, Germany; □ University Cancer Center (UCT), 60590 Frankfurt am Main, Germany; ^ Pharmaceutical Biology, Department of Pharmacy−Center for Drug Research, Ludwig-Maximilians-Universität München, 81377 Munich, Germany; ¢ Institute for Clinical Pharmacology, 9173 Goethe University, 60596 Frankfurt am Main, Germany; + Structural Genomics Consortium (SGC), Buchmann Institute for Life Sciences, 60438 Frankfurt am Main, Germany

## Abstract

The bifunctional soluble epoxide hydrolase (sEH) represents a promising target for inflammation-related diseases. Although potent inhibitors targeting each domain are available, sEH-PROTACs offer the unique ability to simultaneously block both enzymatic functions, mimicking the sEH knockout phenotype, which has been associated with reducing inflammation, including neuroinflammation, and delaying the progression of Alzheimer’s disease. Herein, we report the structure-based development of a potent sEH-PROTAC as a useful pharmacological tool. In order to facilitate a rapid testing of the PROTACs, a cell-based sEH degradation assay was developed utilizing HiBiT technology. We designed and synthesized 24 PROTACs. Furthermore, cocrystallization of sEH with two selected PROTACs allowed us to explore the binding mode and rationalize the most optimal linker length. After comprehensive biological and physicochemical characterization of this series, the most optimal PROTAC **23** was identified in primary human and murine cells, highlighting the potential of using **23** in disease-relevant cell and tissue models.

## Graphical abstract

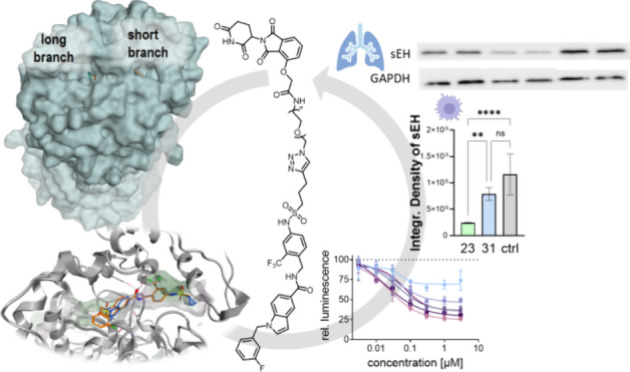

## Introduction

Soluble epoxide hydrolase (sEH) is a ubiquitously expressed bifunctional enzyme encoded by the gene *EPHX2*.^1^ The protein harbors two distinct catalytic domains connected by a proline-rich linker. The C-terminal domain exhibits epoxide hydrolase activity, while the N-terminal domain (sEH-P) represents a lipid phosphatase.^2,3^ sEH plays a pivotal role in the cytochrome P450 branch of the arachidonic acid cascade, as its epoxide hydrolase domain (sEH-H) converts anti-inflammatory epoxyeicosatrienoic acids (EpETrEs) into the corresponding biologically less active diols (dihydroxyeicosatrienoic acids, DiHETrE).^4,5^ Since sEH metabolism is the main pathway for the deactivation of EpETrEs,^6^ it is being pursued as a pharmacological target for multiple inflammatory diseases, and several sEH inhibitors have been developed,^7^ e.g., clinical candidate GSK2256294A (**1**)^8–11^ (Figure 1). The biological role of sEH-P is less well understood, and the endogenous substrates are still being investigated.^12–14^

**Figure 1:**
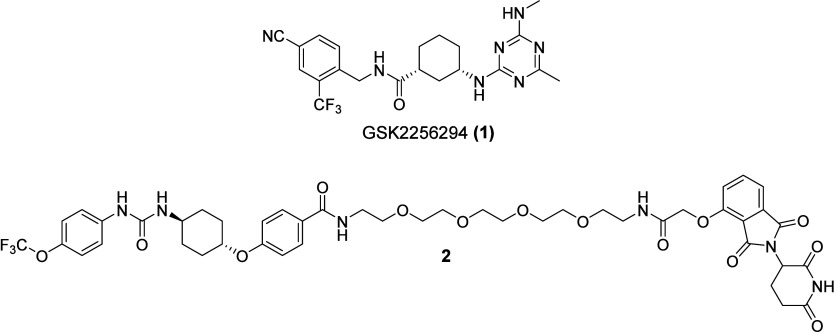
Structures of the potent sEH-H inhibitor and clinical candidate GSK2256294A (**1**) as well as the first-in-class sEH PROTAC **2**.

Depletion of the whole enzyme in sEH knockout mice has been associated with beneficial effects such as reducing inflammation,^15^ attenuating neuroinflammation,^16^ and delaying the progression of Alzheimer’s disease.^17^ Thus, simultaneous inactivation of both domains could be therapeutically advantageous and would also offer the opportunity to further study the biological functions of sEH, as some differences have been noted between the phenotypes of sEH^–/–^ mice and those that receive a sEH-H inhibitor.^18^ Although there are potent inhibitors available for both domains,^7,14^ the application of multiple drugs is often associated with negative safety profiles and drug–drug interactions.^19^ sEH-PROTACs, however, offer the unique opportunity to simultaneously degrade both domains, mimicking the sEH knockout phenotype. Proteolysis targeting chimeras (PROTACs) are heterobifunctional molecules that induce the ubiquitinylation and subsequent proteasomal degradation of a protein of interest (POI) by bringing the POI into close proximity with an E3 ligase, such as cereblon (CRBN) or the Von-Hippel-Lindau E3 ligase.^20^ Recently, Wang et al. reported the development of a first-in-class series of sEH-PROTACs. Their lead compound, **2** (Figure 1), induced sEH degradation in HepG2 and HEK293T cells. Remarkably, **2** was also more effective in reducing ER stress in a phenotypic cellular assay compared to the parent sEH inhibitor.^21^ Peyman et al. likewise demonstrated an attenuation of ER stress in mice.^22^

In this study, we developed a new series of sEH PROTACs supported by a cellular sEH degradation assay based on the HiBiT technology,^23^ allowing for rapid assessment of the degradation ability of synthesized sEH PROTACs. We performed a structure–activity-relationship investigation based on the crystal structure of previously published potent sEH inhibitor FL217 (**3**).^24^ To demonstrate the applicability to study sEH in a cellular context, the most promising PROTAC was tested in primary human and murine cells.

## Results and Discussion

The rational design approach to develop sEH PROTACs was based on the previously published crystal structure of the potent sEH inhibitor FL217 (**3**) (IC_50_ (hsEH) = 8.4 nM in vitro), shown in Figure 2.^24^ The hydrolase domain of sEH possesses a hydrophobic L-shaped binding pocket exhibiting a long branch (∼15 Å) and a short branch (∼10 Å).^24,25^ The amide-based inhibitor **3** functions as a transition state mimetic of the endogenous epoxide substrates: Tyr383 and Tyr466 residues in the active site coordinate to the carbonyl-O via two H-bonds, while Asp335 interacts with the amide-N. Analyzing the crystal structure, two possible exit vectors to address either the exit of the short branch or the long branch of the binding pocket were identified. We rationalized that replacing the cyclopropane ring and attaching a linker to the sulfonamide moiety of **3** would lead to a short branch addressing sEH-H ligand, whereas functionalization at the indole-N would result in a long branch sEH-H ligand (Figure 2). There are many potent sEH-H inhibitors, but we chose compound **3** because it was developed in-house, and therefore, the assay data was comparable. The advantage of FL217 is the opportunity to use the same exit vector functionalization for both designs.

**Figure 2:**
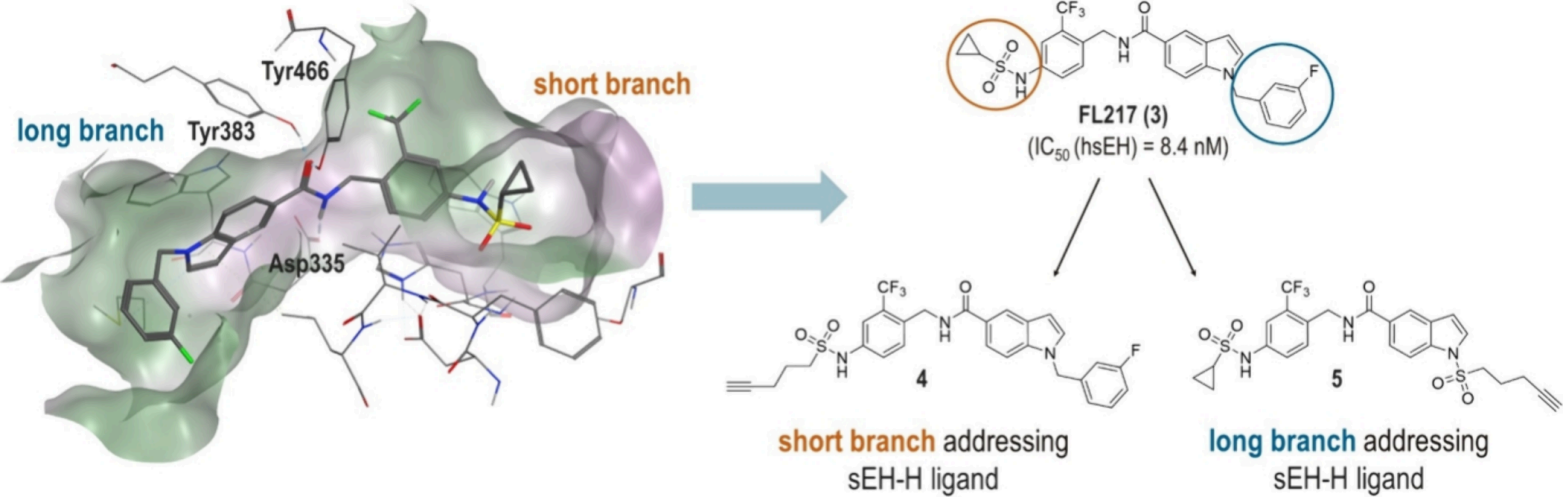
Structure-based design of sEH PROTACs. Left: Crystal structure of sEH-H, cocrystallized with sEH-H inhibitor FL217 (**3**) (PDB: 7P4K). Right: Two sEH-H ligands were developed from sEH-H inhibitor FL217, each bearing a terminal alkyne group for further functionalization.

Molecular docking experiments were performed to identify the appropriate linker length for the inhibitory scaffold to reach the exit of the binding pocket (Figure S1). Based on these results, we modified **3** at both attachment points with a C3-alkyl linker bearing a terminal alkyne group and obtained short branch addressing ligand **4** and long branch addressing ligand **5** (Figure 2). The terminal alkyne handle of these ligands allowed for an efficient variation of linkers and E3 ligase recruiters using the Cu­(I) catalyzed azide–alkyne cycloaddition (CuAAC, “CLICK” reaction) as well as the Sonogashira coupling reaction.

In this study, we recruited the E3 ligase cereblon (CRBN) using derivatives of the well-established CRBN-ligands thalidomide, pomalidomide, and lenalidomide. Linker variation was implemented by the choice of rigid alkyne linkers as well as flexible PEG linkers of different lengths (PEG1–PEG6).

Short branch addressing sEH-H ligand **4** was synthesized from aniline precursor **11** in a nucleophilic substitution reaction with pent-4-yne-1-sulfonyl chloride and pyridine. The synthesis of precursor **11** was performed in three steps according to the previously published procedure by Lillich et al:^24^ Indole ester **6** was substituted with benzyl chloride **7** in a Finkelstein-type nucleophilic substitution reaction. After ester hydrolysis, carboxylic acid **9** was treated with benzylic amine **10** under amide coupling conditions to obtain **11** (Scheme 1).

**Scheme 1:**
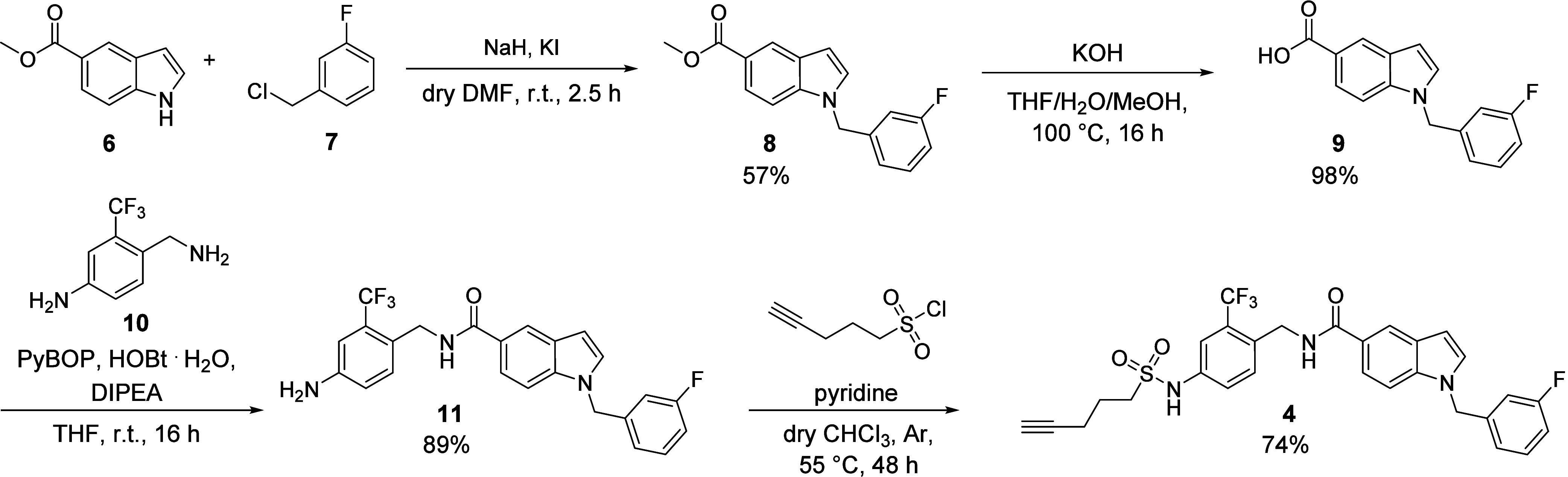
Synthetic Route for sEH-H Ligand **4** Addressing the Short Branch of the Binding Pocket

The synthesis of long branch addressing sEH-H ligand **5** was conducted in four steps. According to the previously published method,^24^ aniline precursor **16** was synthesized from starting material 4-Amino-2-(trifluoromethyl)­benzonitrile (**12**) in three steps. **12** underwent nucleophilic substitution with cyclopropanesulfonyl chloride, followed by reduction of **13** to benzylic amine **14** and subsequent amide coupling with 1*H*-indole-5-carboxylic acid (**15**) to obtain **16**. Lastly, **5** was synthesized from precursor **16** in a nucleophilic substitution reaction with pent-4-yne-1-sulfonyl chloride and NaH (Scheme 2).

**Scheme 2:**
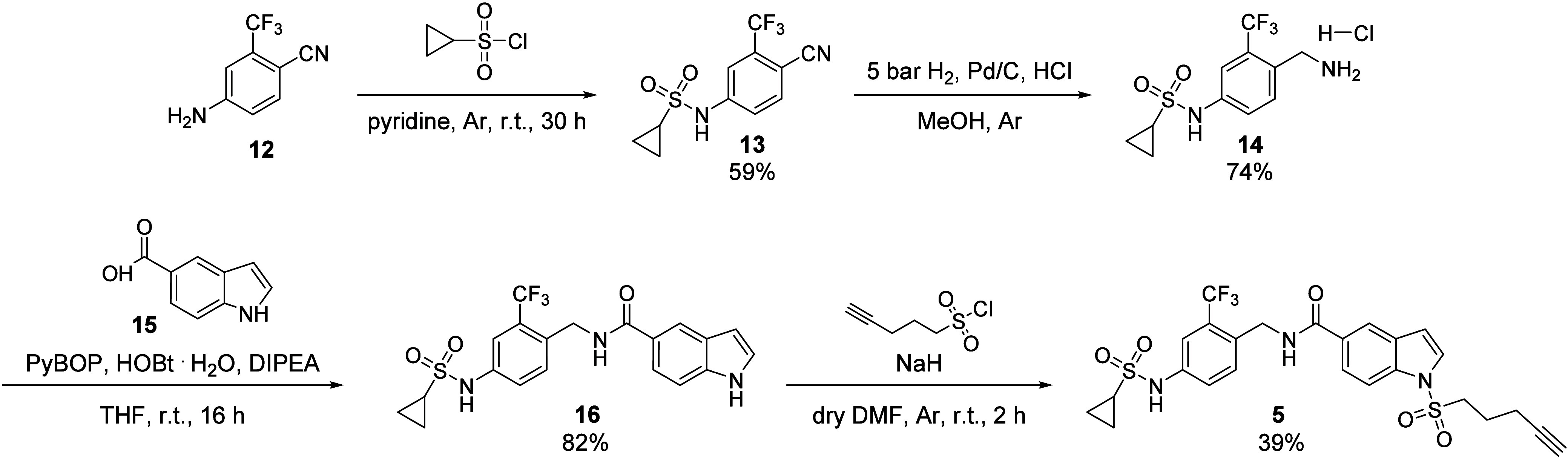
Synthetic Route for sEH-H Ligand **5** Addressing the Long Branch of the Binding Pocket

The set of PROTACs with rigid alkyne linkers (**18a**–**e** and **19a**–**e**) was synthesized under Sonogashira coupling conditions^26^ with both sEH-H ligands **4** and **5** (Scheme 3). Five commercially available Br-functionalized CRBN recruiters were used, from which two were pomalidomide derivatives **17a**–**17b** (functionalized in positions 4 and 5, respectively) and three were lenalidomide derivatives **17c**–**17e** (Br in positions 4, 5, and 6, respectively). With this set of regioisomeric compounds, we aimed to examine the possible impact of the attachment point to the CRBN recruiter on sEH degradation. Position 7 was not taken into consideration for this study, as Fischer et al. demonstrated that this position is not solvent-exposed in the binding pocket of CRBN.^27^

**Scheme 3:**
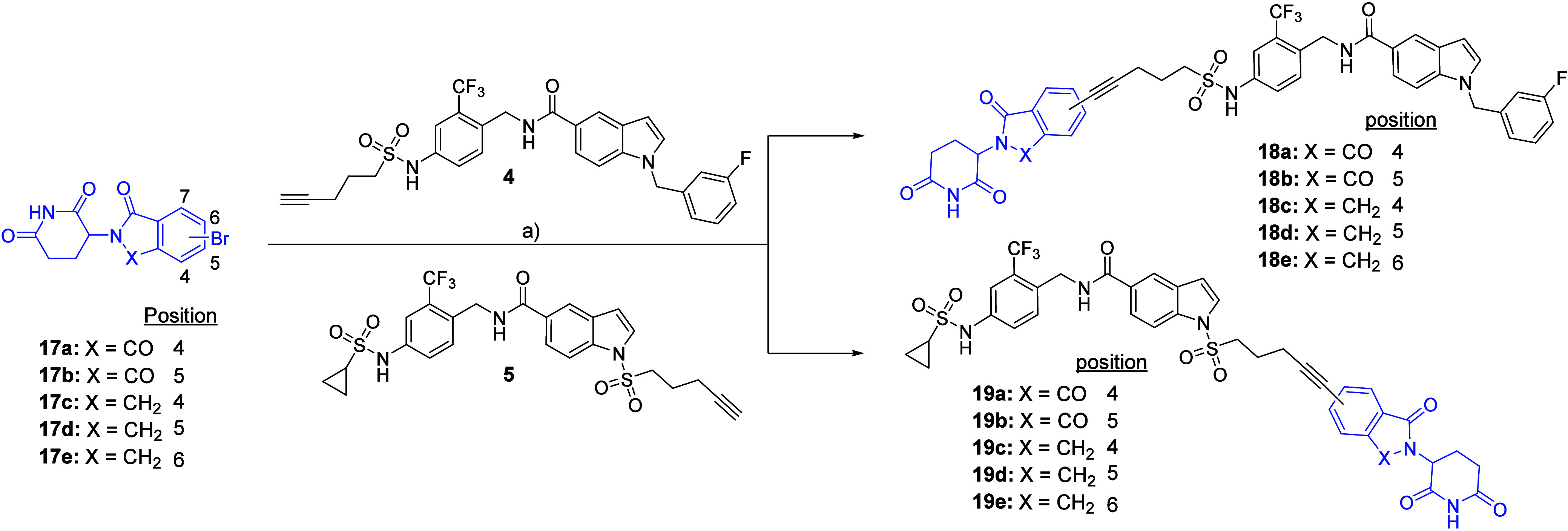
Preparation of PROTACs with Alkyne Linkers^a^

a Reaction conditions (a): Pd­(PPh_3_)_2_Cl_2_ (0.1 equiv), CuI (0.2 equiv), DMF, NEt_3_, 80 °C, 16 h.

For the set of PROTACs harboring PEG linkers, we used commercially available azide-functionalized pomalidomide derivatives (**20a**–**20f**) with linker lengths varying from PEG1 to PEG6. Both sEH-H ligands **4** and **5** were treated with **20a**–**20f** under standard CuAAC conditions^28^ to obtain the corresponding PROTACs **21a**–**21f** and **22a**–**22f** (Scheme 4).

**Scheme 4:**
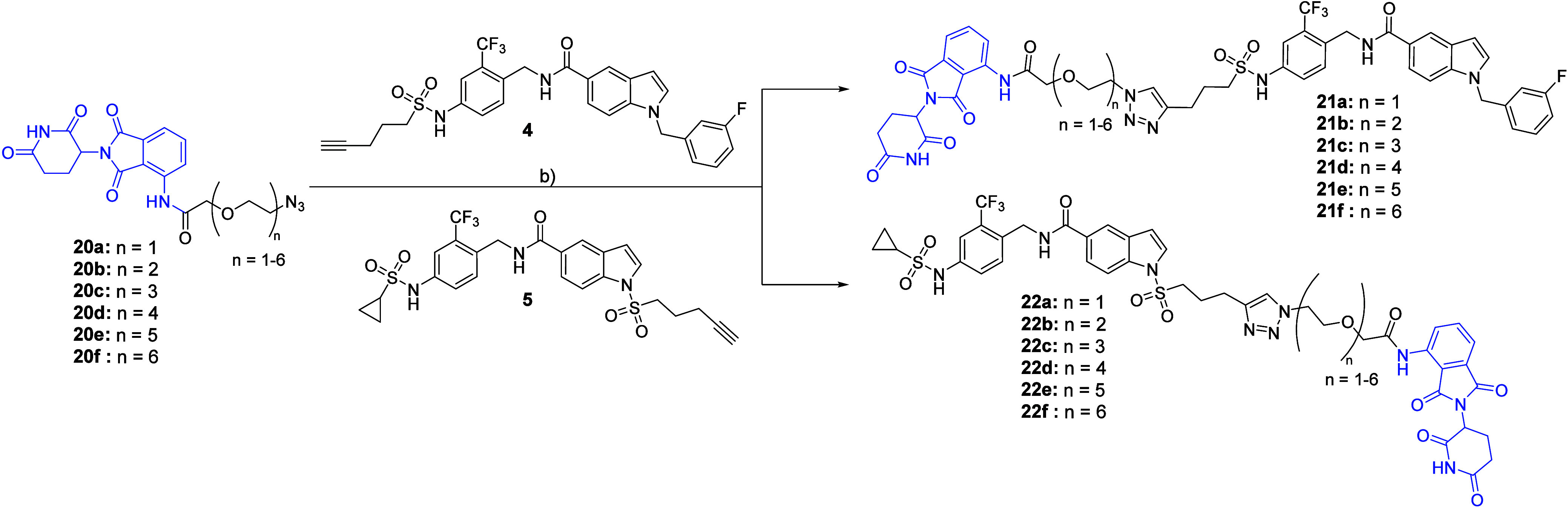
Preparation of PROTACs with PEG Linkers^a^

a Reaction conditions (b): CuSO_4_·5H_2_O (0.3 equiv), sodium ascorbate (0.3 equiv), DMF/H2O = 4:1, r.t., 16 h.

To validate the postulated binding modes of short branch vs long branch addressing PROTACs, the two PEG2 exhibiting PROTACs **21b** and **22b** were cocrystallized with human sEH-H (Figure 3). The obtained cocrystal structures confirmed our hypothesis: while **21b** addressed the exit of the short branch of the binding pocket, **22b** (shown in orange) addressed the long branch exit.

**Figure 3:**
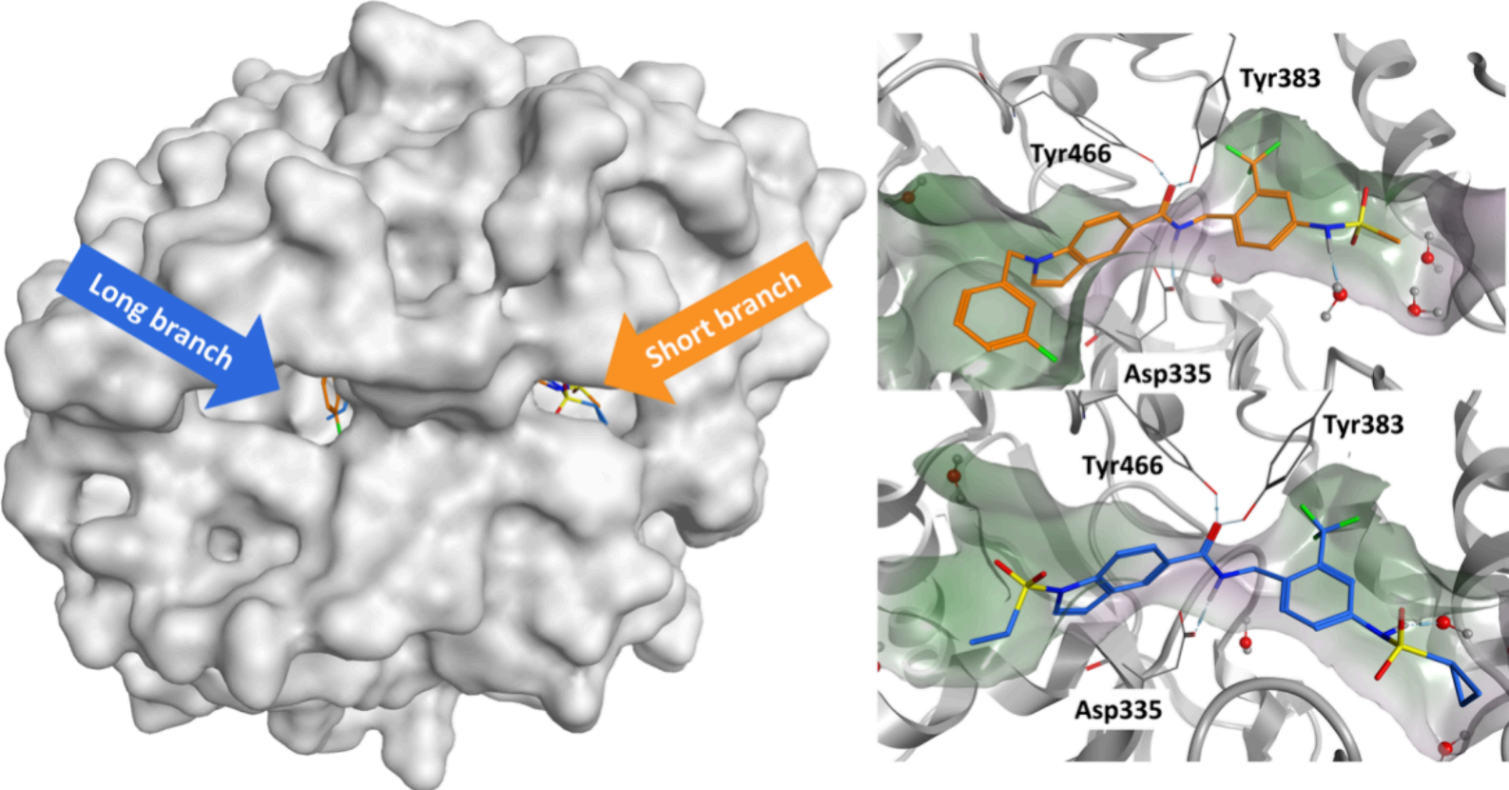
Overlaid cocrystal X-ray structures of **21b** (orange) and **22b** (blue) with human sEH-H (PDB codes: 8S76, 8S77). Left: Protein surface of hsEH-H labeled with both exits of the binding pocket. As predicted, **21b** addressed the exit of the short branch of the binding pocket, while **22b** addresses the exit of the long branch. Right: **21b** and **22b** interact with asparagine 335 from the catalytic triad and tyrosine 446 and tyrosine 383. The PEG2-linkers and CRBN ligands were not resolved.

For all synthesized PROTACs, the in vitro sEH inhibitory potency was determined in an enzyme activity assay with recombinant sEH (murine and human isoforms) and the fluorogenic substrate 3-Phenyl-cyano­(6-methoxy-2-naphthalenyl)­methyl ester-2-oxiraneacetic acid.^29^ All of the compounds exhibited low nanomolar potencies toward human sEH-H (hsEH-H), confirming that the attached linkers were tolerated in the active site of hsEH-H. For the murine isoform, potencies were typically less potent by 1 to 2 orders of magnitude using the short branch exit vector **18a**–**e** and **21a**–**f** (Tables 1 and 2), yet still showed strong activity.

**Table 1:**
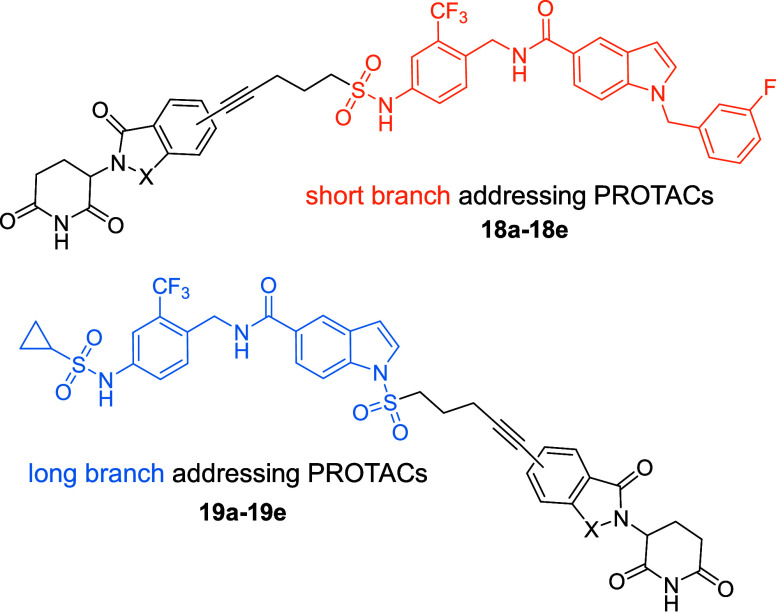

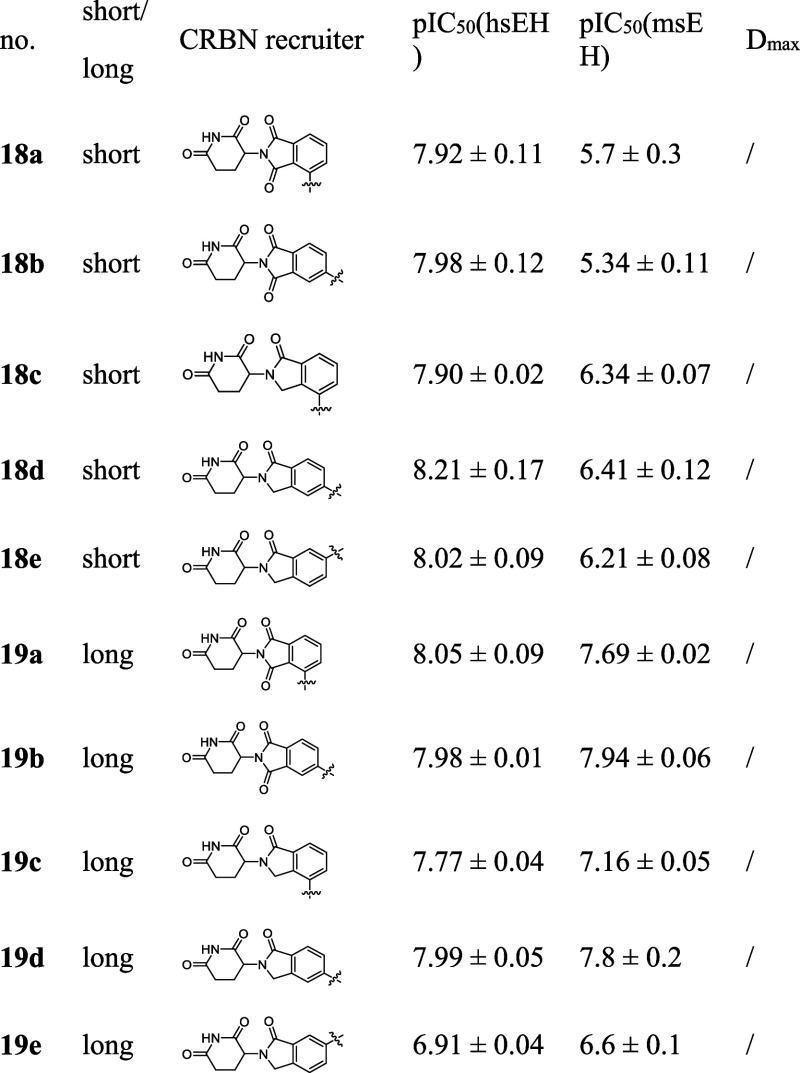
Potential sEH PROTACs with Alkyne Linkers Linked from Different Positions of the CRBN Recruiters

**Table 2:**
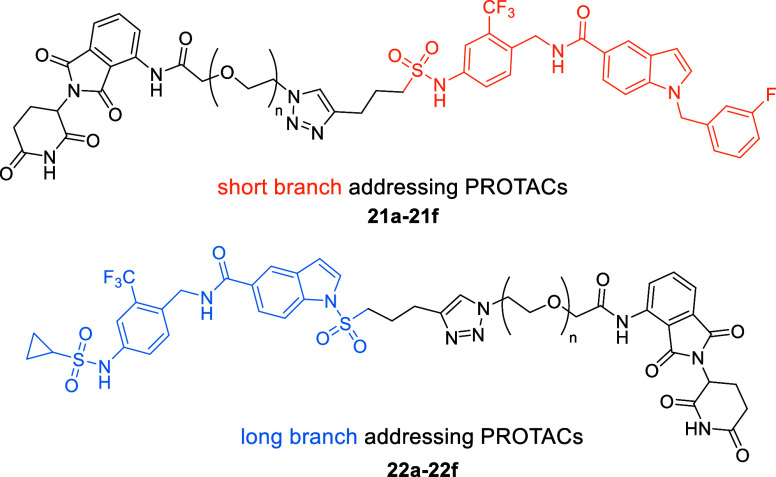

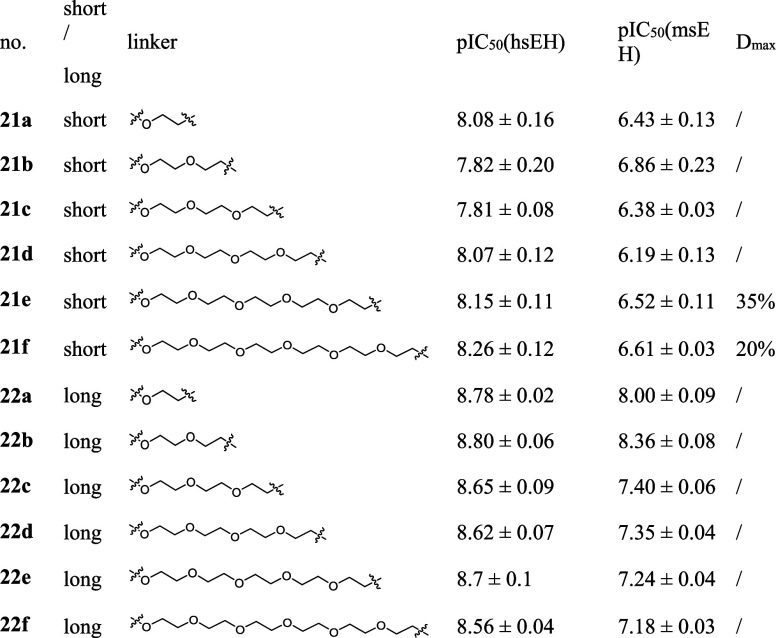
Synthesized sEH PROTACs with PEG Linkers

In order to test PROTACs for their degradation potency, Western blots are predominantly used in the literature. As this method has limited throughput and does not allow for an accurate determination of the PROTAC’s degradation efficiency (*D*_max_) or potency (DC_50_),^30^ a cellular HiBiT-based^23^ degradation assay for sEH was established in this study. The HiBiT system allows us to quantify the cellular level of a protein of interest by detection of luminescence generated by reconstitution of the small enzyme NanoLuc. In this setup, the POI is fused to a small peptide tag of 11 amino acids called HiBiT, which represents a fragment of NanoLuc. HiBiT has a very high affinity for the 18 kDa polypeptide LgBiT, the residual scaffold of the NanoLuc. Thus, degradation of the sEH-HiBiT fusion protein was monitored by a decrease in bioluminescence (Figure 4A). This assay setup allowed for a precise calculation of the maximum percentage of degradation (*D*_max_), marking the PROTAC’s degradation efficiency, as well as the degradation potency DC_50_ (effective concentration to reach 50% of the PROTAC’s *D*_max_).

**Figure 4:**
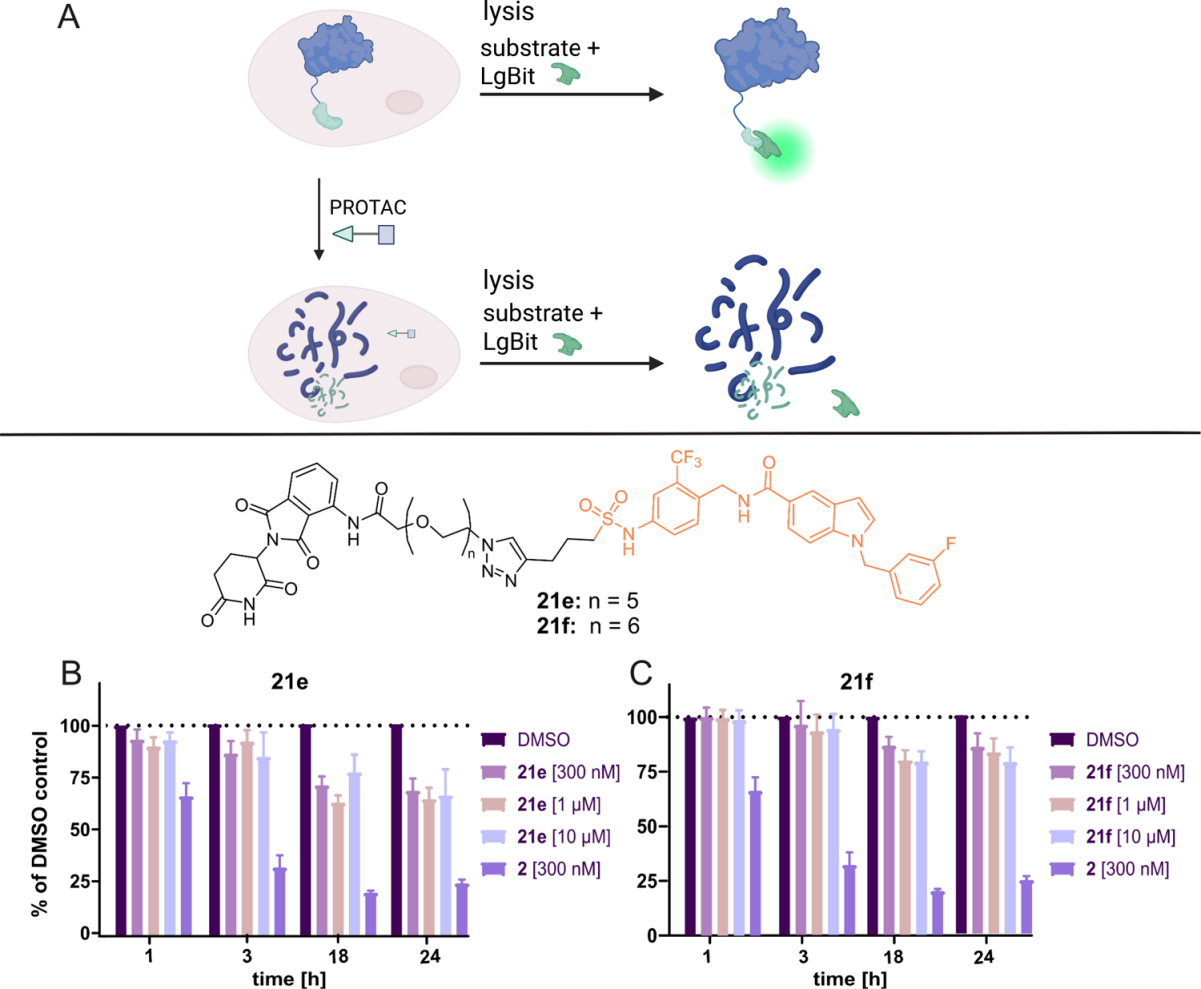
Development of a cellular HiBiT-based sEH degradation assay and testing of the synthesized PROTACs. A: HiBiT assay principle. The figure was prepared using BioRender.com. B and C: Results for short branch addressing sEH PROTACs **21e** with a PEG5 linker (Graph B) and **21f** with a PEG6 linker (Graph C). The best result within this series of PROTACs was obtained for **21e** with *D*_max_ = 35%.

A stable cell line (HeLa^sEH‑HiBiT^) was generated in which the construct containing human sEH (aa1-aa555) followed by a linker and C-terminal HiBiT, is expressed under the control of a constitutive HpGK promoter (for coding and protein sequences see SI). HeLa cells were stably transfected with the construct using the Sleeping Beauty method. The assay was set up in a 384-well plate format with 2000 cells per well in a total volume of 55 μL. After treatment of the cells with the respective compounds for different incubation intervals, cells were lysed and LgBit and the substrate were added (Promega Corporation), followed by luminescence detection using a plate reader. As a positive control, the recently disclosed sEH PROTAC **2** was used. PROTAC **2** ^21^ was tested at eight concentrations ranging from 0.001 to 3 μM along with DMSO treatment, and cells were incubated for 1, 3, 18, and 24 h, respectively. The luminescence signals obtained were plotted relative to the solvent control (0.5% DMSO) against the concentration of **2** (Figure S2A). Consistent with the original report on this compound, complete loss of sEH was not achieved; a phenomenon attributed to the selective degradation of the enzyme in the cytosol but not in peroxisomes. In line with the literature data, a hook effect was observed at 3 μM concentration of **2** after 1 and 3 h treatment. Two control experiments were performed in order to validate the PROTAC-induced degradation. First, cells were cotreated with **2** (300 nM) and sEH-H inhibitor **1** (3 μM). As expected, this led to a rescue from degradation, indicating that the degradation was mediated by the PROTAC binding to the active site of sEH-H. Furthermore, when cells were cotreated with PROTAC **2** (300 nM) and the proteasome inhibitor MG132 (3 μM), the luminescence signal increased to almost 100% of that of the solvent control. The latter observation indicated that the PROTAC-dependent loss of sEH protein required the proteasomal degradation pathway (Figure S2B), and contradicts the previous report that the lysosomal inhibitor Bafilomycin A1 (BafA1) was more effective than MG132.

We initially tested the synthesized sets of PROTACs at three concentrations (10.0, 1.0, and 0.1 μM) using the established HeLa^sEH‑HiBiT^ cell line monitoring sEH levels after 1, 3, 18, and 24 h. None of the PROTACs harboring an alkyne linker (**18a**–**e** and **19a**–**e**) decreased luminescence compared to DMSO-treated cells at any concentration or incubation time. The same was observed for long branch addressing compounds with PEG1-PEG6 (**22a**–**22f**) as well as short branch addressing compounds with PEG1-PEG4 linkers (**21a**–**21d**). However, the short branch addressing PROTACs **21e** and **21f**, containing a PEG5 and PEG6 linker, showed a significant signal decrease of 35% and 20%, respectively (Figure 4B,C). Thus, the most promising compound was **21e**, a combination of the short branch addressing inhibitor scaffold **4** and a PEG5 linker.

Next, the short branch addressing inhibitor scaffold as well as the linker length of **21e** were kept constant, but the CRBN ligand was changed from the amide functionalized pomalidomide derivative to an ether-functionalized thalidomide derivative. Structurally, the resulting compound **23** was a constitutional isomer of hit compound **21e**. Additionally, we aimed to examine whether the triazole-bridge of **21e** may have a limiting effect on sEH degradation, and also synthesized the amide analogue **24**, maintaining the ether functionalized thalidomide (Scheme 5).

**Scheme 5:**
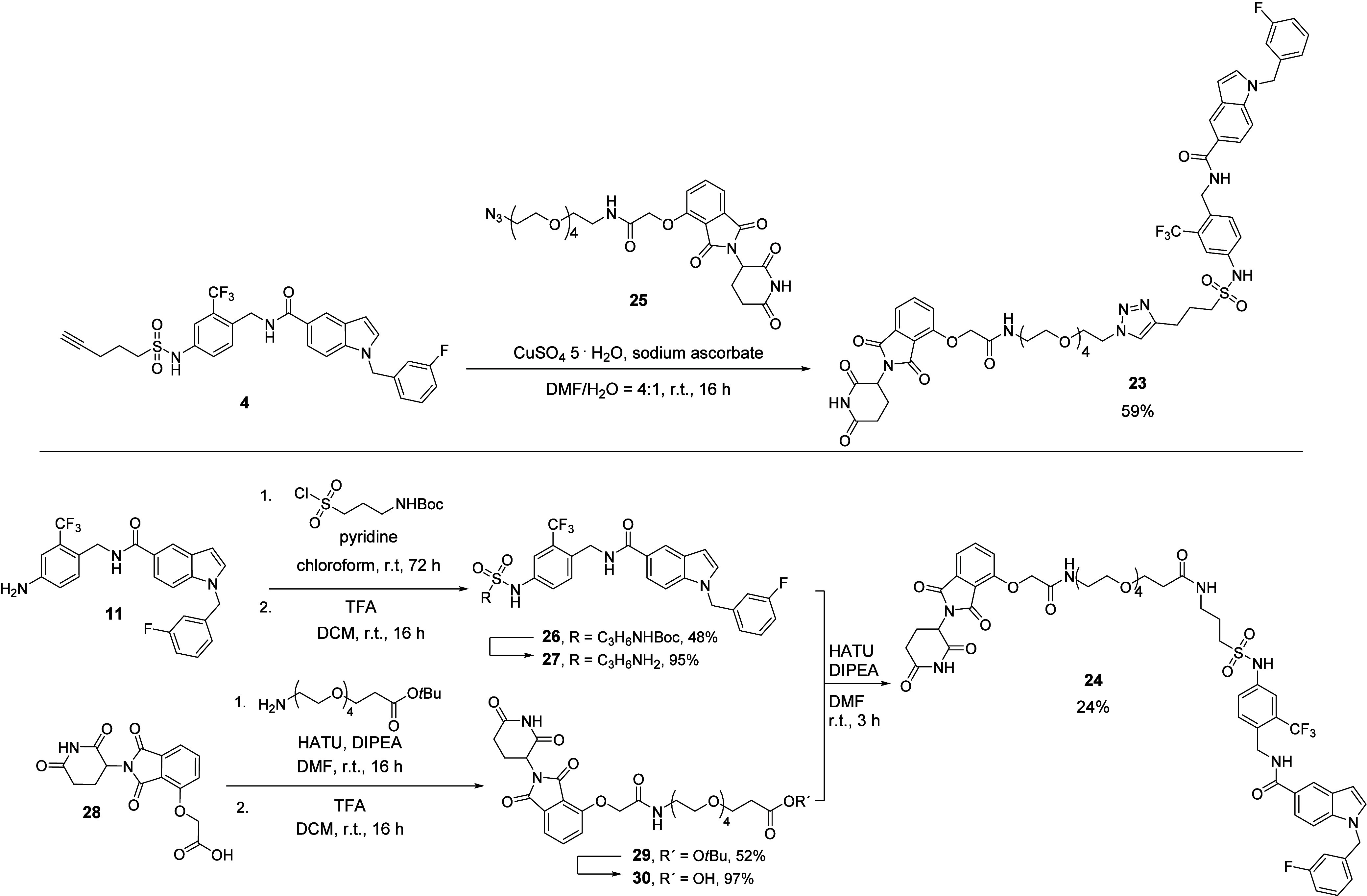
Synthetic Routes for the Synthesis of sEH PROTACs **23** and **24**

**23** was synthesized from the short branch sEH-H ligand **4** and the commercially available building block **25** under CuAAC conditions (Scheme 5, upper panel). In order to synthesize amide-based PROTAC **24**, a new FL217-based sEH-H ligand **27** with a primary amine handle for amide coupling was synthesized. **27** was synthesized in two steps from previously synthesized aniline derivative **11**: A nucleophilic substitution reaction was performed with *tert*-Butyl (3-(chlorosulfonyl)­propyl)­carbamate and pyridine to afford compound **26**, which was Boc deprotected with trifluoroacetic acid (TFA). sEH-H ligand **27** was then treated in an amide coupling reaction with building block **30**, which had been synthesized in two steps before from **28** (Scheme 5, lower part).

Both compounds were analyzed for their inhibitory potency toward hsEH-H and msEH-H, as described for the first set of PROTACs (Table 3). As previously observed for other short-branch addressing compounds, both PROTACs **23** and **24** exhibited excellent low nanomolar potencies toward hsEH-H, but were less potent (up to 2 orders of magnitude) toward the murine isoform. Interestingly, the triazole-containing PROTAC **23** was more potent than its amide-containing analogue **24**.

**Table 3:**
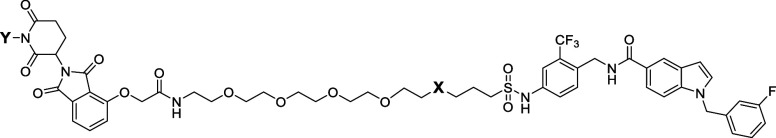

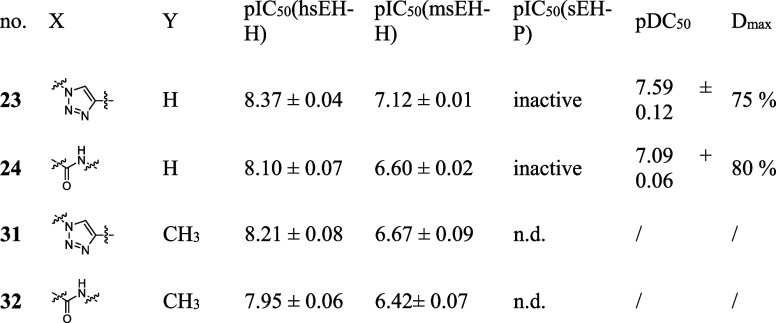
Biochemical Characterization of the Optimized PROTACs **23** and **24** and the Corresponding Methylated PROTACs **31** and **32**

Next, both PROTACs were tested in the sEH-HiBiT assay. In a first experiment, three concentrations were screened (0.3, 1.0, and 3.0 μM), and cells were incubated for up to 24 h. One-hour incubation with the triazole-based PROTAC **23** (0.3 and 1.0 μM) was sufficient to elicit a 25% degradation of the sEH, while the amide-basedPROTAC **24** induced a 50% degradation of the protein. After 18 h, the effectiveness increased to 75% for **23** and 80% for **24**. Next, the tests were expanded to include seven concentrations of each compound (3 nM–3 μM) and six different incubation times (up to 24 h). The resulting dose- and time-response curves (Figure 5A,B) revealed that both PROTACs induced comparable sEH degradation: *D*_max_ = 75% and *D*_max_ = 80% for **23** and **24**, respectively. Both compounds also showed high degradation potencies in the nanomolar range and the highest *D*_max_ after 18 h, with a slight recovery after 24 h. The same behavior has been reported for the positive control, i.e., compound **2** (Figure S2A). Indeed, in experiments in which the incubation was extended up to 48 h, the overall level of degradation achieved was reduced, i.e., only 25% using PROTAC **23** and 30% using compound **24** (Figure S3). This finding may indicate a compensation mechanism leading to rapid resynthesis of sEH in the cells, or perhaps more likely, to the rapid metabolism and inactivation of the PROTACs in the cell-based assay.

**Figure 5:**
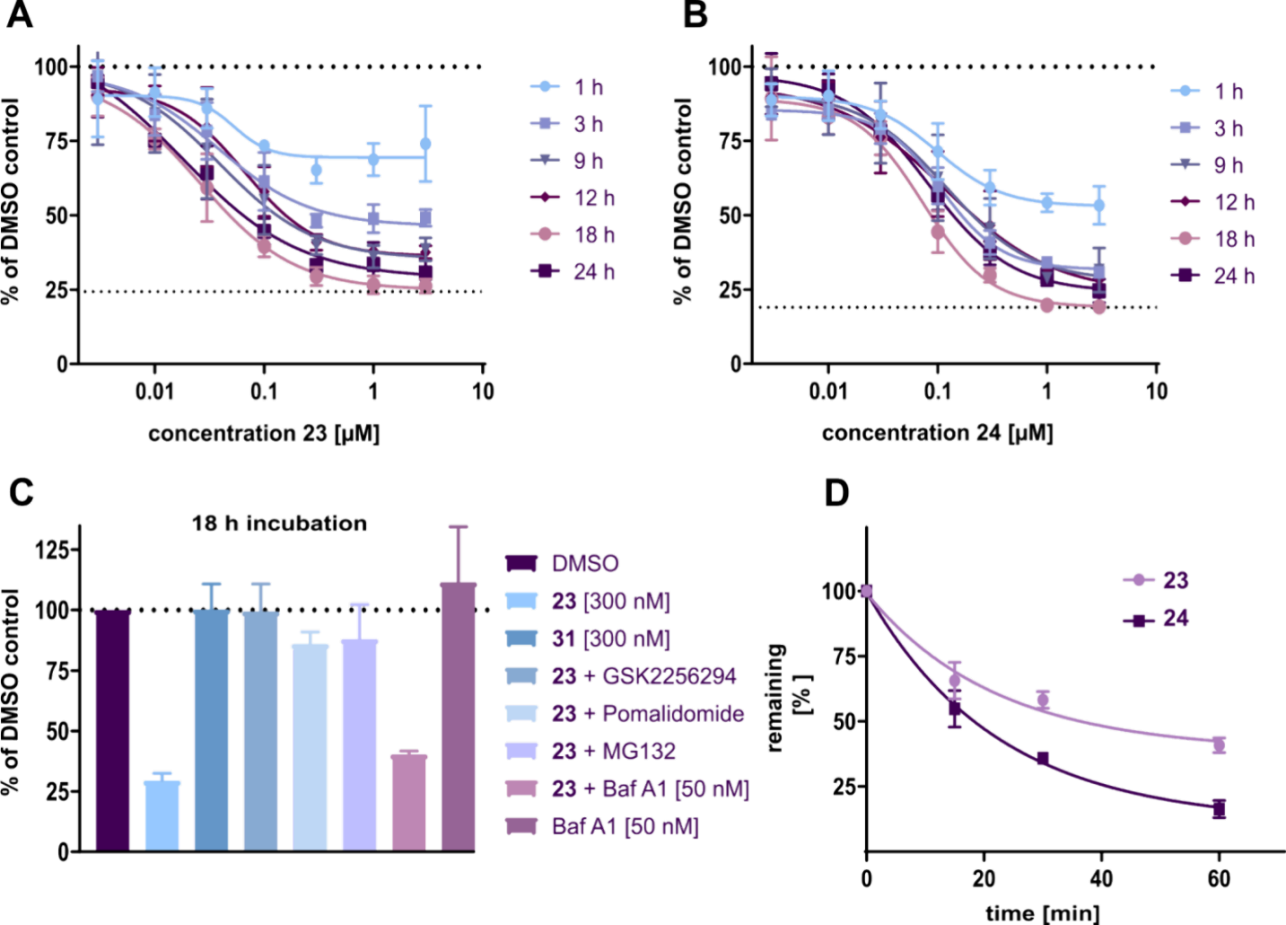
Biochemical characterization of optimized PROTACs **23** and **24**. A: degradation curves of triazole-based PROTAC **23** for different incubation times (*N* = 3). B: Degradation curves of amide-based PROTAC 24 for different incubation times (*N* = 3). C: Control experiments for PROTAC **23** (*N* = 3). HeLa^sEH-HiBiT^ were cotreated with **23** [300 nM] and sEH-H inhibitor GSK2256294 [3 μM] or CRBN ligand Pomalidomide [3 μM] or proteasome inhibitor MG 132 [3 μM] or lysosome inhibitor Bafylomicin A1 [50 nM] for 18 h. D: Evaluation of metabolic stability of PROTACs **23** and **24** in rat liver microsomes.

A set of control experiments^31^ was performed with both compounds **23** and **24** in order to validate their mechanism of action. This involved synthesizing corresponding negative control compounds referred to here as compounds **31** and **32** (for experimental details, see SI). The control compounds were methylated on the glutarimide-N of the PROTAC thalidomide moiety to prevent CRBN binding and sEH degradation, but not the ability of the compounds to inhibit sEH-H activity. Indeed, compounds **31** and **32** were strong sEH-H inhibitors, but they did not induce protein degradation (Table 3, Figures 5C and S4). Next, cells were cotreated with the respective PROTAC and an excess of the potent sEH-H inhibitor **1** or the CRBN binder pomalidomide. Both cotreatments resulted in the restoration of the luminescence, indicating that ternary complex formation is essential for degradation. These experiments demonstrated target engagement with the active site of sEH-H and not the sEH-P domain. This was confirmed in an in vitro sEH-P activity assay, in which PROTACs **23** and **24** were inactive (Table 3). In order to determine whether sEH degradation involved the proteasomal or the lysosomal degradation pathway, cells were cotreated with PROTAC **23** and the proteasome inhibitor MG132 or the lysosomal inhibitor bafilomycin A1. As observed previously when studying the effects of PROTAC **2**, cotreatment with MG132 restored the signal, while bafilomycin A1 was without effect. Taken together, these results suggested that the PROTAC-induced degradation of sEH was CRBN-dependent and relied on the proteasome.

Optimized PROTACs **23** and **24** were further characterized regarding their physicochemical and in vitro pharmacokinetic properties, as well as their cytotoxicity. The water solubility of both PROTACs was determined to be in a range between 3 and 5 μM (Figure S5A). The compounds did not induce any significant toxicity as HepG2 cell viability was unaffected even after 72 h exposure to the highest concentration tested (50 μM) (Figure S5B). Finally, the in vitro metabolic stability was determined in rat and murine liver microsomes. Rat liver microsomes metabolized 84% of compound **24** within 60 min, and 59% of compound **23** (Figure 5D). Murine microsomes were less effective, with 48% of compound **23** and 34% of compound **24** remaining after 1 h (Figure S5C). Based on these data and the enhanced degradation potency (Table 3), the triazole-containing PROTAC **23** was tested in further cell-based experiments. A target engagement assay was performed in HeLa^sEH‑HiBiT^ cells, and the lipid metabolites resulting from sEH-H activity were analyzed after treatment with **23** and **31**. LC-MS/MS was used to analyze the sEH-H activity by monitoring the generation of the sEH products: 14,15-dihydroxy-5*Z*,8*Z*,11*Z*-eicosatrienoic acid (14,15-DiHETrE), 11,12-dihydroxy-5*Z*,8*Z*,14*Z*-eicosatrienoic acid (11,12-DiHETrE), and 8,9-dihydroxy-5*Z*,11*Z*,14*Z*-eicosatrienoic acid (8,9-DiHETrE). After treatment with PROTAC **23** or its negative control **31**, sEH-H activity was reduced, as indicated by a reduced level of detected dihydroxy fatty acids (Figure 6A). PROTAC **23** and negative control **31** exhibit similar sEH-H inhibitory activity (Table 3), and the observed difference in metabolite levels could be explained by the degradation and loss of function of sEH by **23** (Figure 6B), resulting in the formation of less metabolites and lower protein expression compared to the vehicle control.

**Figure 6:**
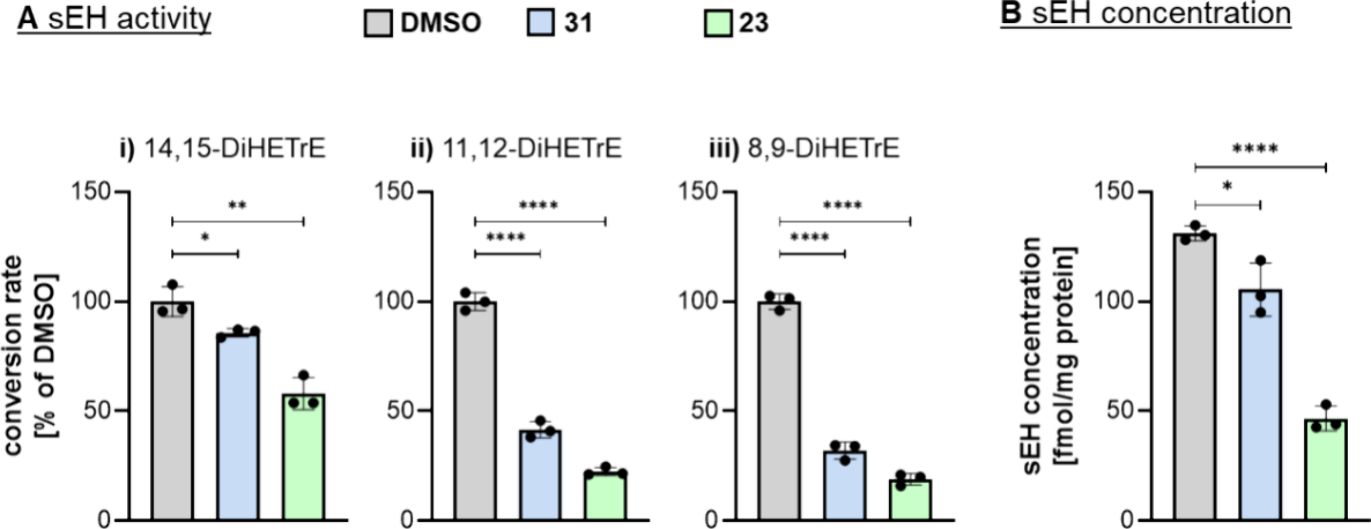
A: Conversion of 14(15)-, 11(12)- and 8(9)-EpETre to the corresponding sEH products (i) 14,15-, (ii) 11,12-, and (iii) 8,9-DiHETrE). The enzyme assay was carried out using cell homogenates of HeLa^sEH-HiBiT^ cells (*n* = 3) incubated with 0.5% DMSO or 3 μM of the PROTACs **23** or **31**) for 3 h at 37 °C. The cell homogenates (0.5–1.3 mg protein/mL) were incubated for 30 min with a mixture of 14(15)-, 11(12)-, and 8(9)-EpETre (20–70 μM).^36–39^ To distinguish nonenzymatic hydrolysis and sEH conversion the hydrolysis occurring after addition of sEHi TPPU (1 μM) was subtracted from the product formation. Shown is the conversion rate as % of the DSMO control (mean ± SD, *n* = 3). B: sEH concentration in the cells determined by LC-MS/MS based targeted proteomics analysis. Shown is the concentration per mg cellular protein (mean ± SD, *n* = 3). Statistical analysis was performed using unpaired Students *t* test comparing each treatment with DMSO control (*: *p* < 0.05; **: *p* < 0.01; ****: *p* < 0.0001).

In order to further examine the usefulness of PROTAC **23** as in a more in vivo situation, we studied its impact on sEH expression in human monocyte-derived macrophages. Human macrophages express sEH following inflammatory activation with lipopolysaccharide and interferon γ.^32^ The addition of the inactive PROTAC, i.e., compound **31**, to inflammatory activated macrophages did not affect sEH expression, while the protein was barely detectable in cells treated with compound **23** (Figure 7). These results indicate that PROTAC **23** is able to initiate the degradation of sEH in primary human cells.

**Figure 7:**
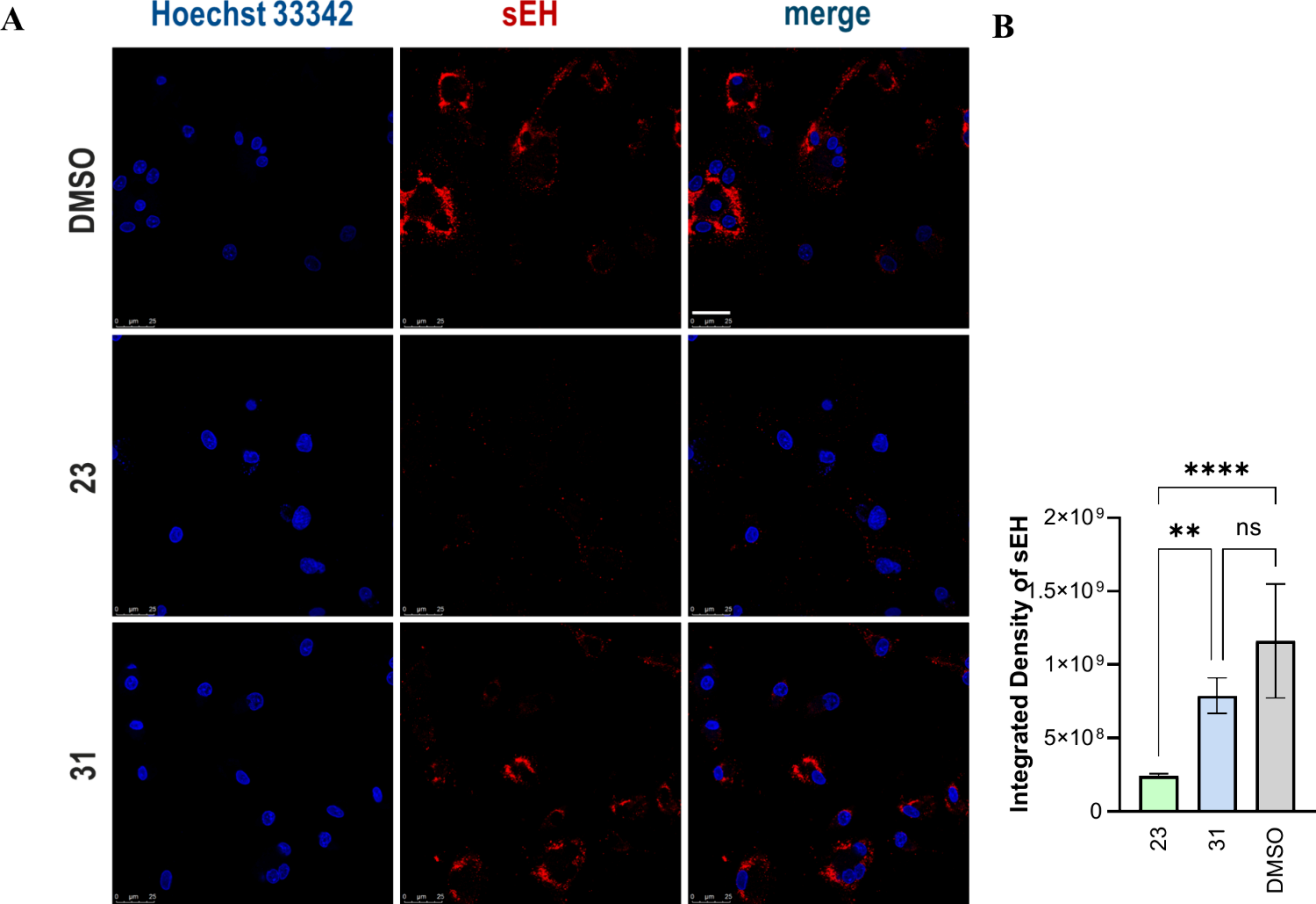
Immunofluorescence staining experiments of sEH in human primary M1 macrophages treated with 300 nM **23** or negative control compound **31** or untreated (DMSO). A: Cell nuclei were marked with Hoechst 33342 (λ_ex_: 325–375 nm, λ_em_: 435–485 nm), sEH was stained via immunocytochemistry (Alexa633, λ_ex_: 590–650 nm, λ_em_: 662–738 nm). Cell nuclei and sEH were visualized by fluorescence microscopy at 63× magnification. Representative experiment is shown. Scale bar: 25 μm. *n* = 3. B: Data are expressed as mean ± standard error. Statistical calculations were carried out using GraphPad Prism software. Statistical significance was determined by one-way ANOVA with Tukey’s posthoc test. ns not significant, **p < 0.01, ****: *p* < 0.0001).

Next, we performed immunoblotting for sEH relative to β-actin as a housekeeping protein in DMSO or PROTAC-treated primary murine hepatocytes. Also here, PROTACs **23** and **24** elicited a significant degradation in sEH levels (Figure 8A). However, a comparison of the efficiency of the degradation in human macrophages versus murine hepatocytes was clearly distinct, with a residual expression of 10% in macrophages treated with PROTAC **23**, versus a residual expression of 60% in hepatocytes. In this assay, a small effect of compound **31** on sEH levels was apparent, which went hand in hand with decreased β-actin levels, possibly indicating a slight hepatotoxic effect. Also, in an isolated tissue, the PROTACs were shown to be effective. Indeed, in precision-cut human lung slices, PROTAC **23** effectively decreased sEH levels while the negative control **31** was ineffective (Figure 8A). Quantitative MS-based proteomics analysis of the tissue was performed, and sEH levels were reduced in comparison to untreated tissue (Figure S6). sEH has only medium abundance in lung tissue.^33^ In the context of lung diseases, studies have shown that sEH inhibitors can mitigate inflammation, improve lung function, and reduce tissue damage. A phase I clinical trial in patients with chronic obstructive pulmonary disease (COPD) has shown that sEH inhibitors may exhibit potential therapeutic benefit in patients with COPD.^11^ The results demonstrated the applicability of PROTAC **23** to study the function of sEH in primary cells as well as complex distal lung tissue, highlighting its successful transition to nonartificial systems across different species.

**Figure 8:**
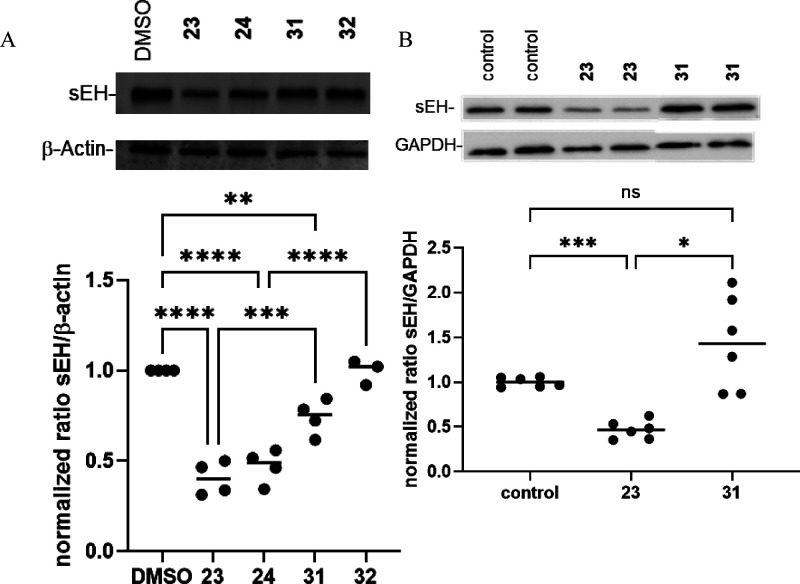
Immunoblotting of sEH in different primary cells and tissues. A: For immunoblotting primary murine hepatocytes were treated 18 h with either DMSO (0.5%), PROTACs **23** and **24**, or negative control compounds **31** and **32** (300 nM). Data are expressed as means ± SE (*n* = 3–4). Statistical analysis was performed using the ordinary one-way ANOVA function in Prism10 comparing each treatment with the DMSO vehicle (**: *p* < 0.01; ***: *p* < 0.001, ****: *p* < 0.0001). B: human PCLS were cultivated in media (DMEM-F12; control) or treated with **23** and **31** (300 nM) for 18 h (*n* = 3). For immunoblotting, duplicate measurements were assessed and statistical analysis was performed using the ordinary one-way ANOVA function in Prism10 (ns = not significant; *: *p* < 0.05 ***: *p* < 0.001).

## Conclusions

In this study, we successfully developed and characterized a novel series of sEH-targeting PROTACs, designed to achieve the degradation of both enzymatic domains. Through a rational design approach based on the crystal structure of potent sEH-H inhibitor **3**, we identified two possible exit vectors for linker attachment and explored a range of linker chemistries, ultimately leading to the identification of the most promising PROTAC, compound **23**.

The development of a high-throughput cellular sEH degradation assay based on the HiBiT technology significantly facilitated the rapid evaluation of our synthesized PROTACs, allowing for precise determination of degradation efficiency (*D*_max_) and potency (DC_50_). Our structure–activity relationship study revealed that short branch addressing PROTAC **23** exhibited the highest degradation potency, demonstrating activity in primary human and murine cells. The cocrystallization of selected PROTACs with sEH confirmed the proposed binding modes and helped rationalize the observed structure–activity relationships.

Biological and physicochemical characterization of compound **23** highlighted its robust degradation profile, with a peak *D*_max_ of 75% observed after 18 h of treatment. Importantly, control experiments validated the PROTAC-mediated degradation mechanism, confirming that sEH degradation was dependent on target engagement and proteasomal activity. It still needs to be clarified whether the complete degradation of sEH (>90%) can be achieved by a PROTAC, as sEH is partially localized in peroxisomes and is not accessible to the proteasome system. In conclusion, our findings provide compound **23** as a promising tool to investigate if PROTAC-based degradation of sEH represents a viable strategy to mimic the beneficial effects observed in sEH knockout models, for further investigations into sEH biology and its pathophysiological role. Future work will focus on optimizing pharmacokinetic properties and evaluating in vivo efficacy to advance sEH-targeting PROTACs toward therapeutic application.

## Experimental Section

Starting materials, reagents, and solvents were purchased from Sigma-Aldrich (Merck), abcr, Fluka, BLDpharm, and FluoroChem and used without further purification. Solvents were of reagent grade or dry, if specified. Thin-layer chromatography (TLC) was performed on ALUGRAM silica gel sheets purchased from Macherey-Nagel, and spots were visualized by UV light (245 and 365 nm). Flash chromatography was performed on a *puriFlash XS 420* device from *interchim* with a *SPD-20A UV/vis* detector from *Shimadzu.* For normal phase chromatography, Silica HP 30 μm columns from *Interchim* were used (size F0012, F0025, or F0040, depending on the scale of the reaction), and solvents were technical grade. Reversed phase chromatography was conducted using C18 HP 30 μm columns from *Interchim* (size F0012, F0025, or F0040, depending on the scale of the reaction), eluents were acetonitrile (HPLC grade) and Milli-Q purified water. NMR spectra were recorded on either a Bruker DPX-250, Bruker Avance-300, Bruker Avance-400, or Bruker Avance-500 spectrometer. Spectra were calibrated on the nondeuterated solvent residue peak (CDCl_3_ = 7.26 ppm or DMSO-*d*_6_ = 2.50 ppm). ^1^H NMR data are reported with the chemical shift δ (ppm) relative to tetramethylsilane, integrals, multiplicity, and coupling constant (Hz). For ^13^C NMR and ^19^F NMR signals, chemical shifts δ (ppm) are reported. HPLC-MS analysis was performed with a flow rate 1 mL/min on a Shimadzu prominence separation device (column Luna 10 μC18(2) 100A (250 × 4.60 mm) by Phenomenex) connected with a Shimadzu SPD-20A UV/vis UV-detector (monitoring at 254 and 280 nm) and a Shimadzu LCMS-2020 Single Quadrupole mass spectrometer with electrospray ionization (ESI). The final compounds were purified using a Shimadzu prominence preparative HPLC system (column Luna 10 μC18(2) (250·21,20 nm) by Phenomenex) with UV monitoring at 254 and 280 nm and a flow rate of 21 mL/min. For both analytical and preparative HPLC, the mobile phase consisted of ACN/0.1% aqueous formic acid. Gradient for method A: 70% 0.1% aqueous formic acid for 10 min, 5 min of lowering 0.1% aqueous formic acid to 10%. Gradient for method B: 95% 0.1% aqueous formic acid for 10 min, 5 min of lowering 0.1% aqueous formic acid to 10%. High-resolution mass spectra were recorded on either a MALDI LTQ Orbitrap XL instrument or a QExactive plus Thermo instrument equipped with a heated electrospray source (HESI) from Thermo Scientific. Purity is >95% of all tested compounds determined through HPLC-MS analysis. The compound **4**, **9**, **11**, **13**, **14**, **16** was synthesized according to a published method.^24^

### General Procedure 1 (GP1): Synthesis of PROTACs **18a**–**19e** with Alkyne Linkers

To a stirred solution of sEH-H ligand **4** *or* **5** (1.0 equiv) in dry DMF (0.2 mL) and Et_3_N (0.2 mL) were added the corresponding bromide of the CRBN recruiter (1.0 equiv), as well as CuI (0.2 equiv) and bis­(triphenylphosphine)­palladium­(II) dichloride (0.1 equiv), were added. The reaction mixture was stirred at 80 °C under an argon atmosphere for 16 h. Afterward, the mixture was diluted with EtOAc and washed with a saturated aqueous solution of NH_4_Cl (3 × 20 mL). The aqueous phase was then extracted with EtOAc (3 × 20 mL). The combined organic phase was dried over MgSO_4_, filtered, and concentrated in vacuo. The crude product was purified using reversed-phase flash chromatography (ACN/H_2_O = 30:70 → 90:10) and, subsequently, if mentioned, using semipreparative HPLC.

### General Procedure 2 (GP2): Synthesis of PROTACs **21a**–**22f** and **23** with PEG Linkers

In a microtube, sEH-H ligand 4 or 5 (60 mM in the reaction, 1.0 equiv) and the corresponding azide of the CRBN recruiter (1.1 equiv) were dissolved in DMF, and the respective aqueous solutions of CuSO_4_·5H_2_O (0.3 equiv) and sodium ascorbate (0.3 equiv) were added subsequently (solvent ratio DMF: H_2_O = 4:1). The reaction mixture was stirred at r.t. for 16 h and then directly subjected to preparative HPLC purification.

#### *N*-(4-((5-(2-(2,6-Dioxopiperidin-3-yl)-1,3-dioxoisoindolin-4-yl)­pent-4-yn-1-yl)­sulfonamido)-2-(trifluoromethyl)­benzyl)-1-(3-fluorobenzyl)-1*H*-indole-5-carboxamide (**18a**)

**18a** was synthesized according to GP1 from **4** (19 mg, 0.033 mmol) and 4-bromo-2-(2,6-dioxopiperidin-3-yl)­isoindoline-1,3-dione (11 mg, 0.033 mmol) as well as CuI (1.3 mg, 0.007 mmol) and bis­(triphenylphosphine)­palladium­(II) dichloride (2.4 mg, 0.003 mmol). The product was obtained after RP flash chromatography as a colorless solid in 33% yield (9 mg, 0.01 mmol). ^1^H NMR (500 MHz, DMSO-*d*_6_): δ = 11.12 (s, 1H), 10.19 (s, *J* = 26.0 Hz, 1H), 8.86 (t, *J* = 5.8 Hz, 1H), 8.20 (s, 1H), 7.86–7.77 (m, 2H), 7.72–7.66 (m, 2H), 7.62 (d, *J* = 3.2 Hz, 1H), 7.54 (d, *J* = 8.9 Hz, 2H), 7.49–7.44 (m, 2H), 7.38–7.31 (m, 1H), 7.11–7.05 (m, 1H), 7.04–6.98 (m, *J* = 10.7, 8.1 Hz, 2H), 6.63 (d, *J* = 3.2 Hz, 1H), 5.50 (s, 2H), 5.13 (dd, *J* = 12.8, 5.4 Hz, 1H), 4.54 (d, *J* = 5.3 Hz, 2H), 3.44–3.39 (m, 2H), 2.92–2.83 (m, 1H), 2.69 (t, *J* = 7.0 Hz, 2H), 2.62–2.57 (m, 1H), 2.54–2.51 (m, *J* = 4.3, 2.6 Hz, 1H), 2.10–1.96 (m, 3H). ^13^C NMR (126 MHz, DMSO-*d*_6_): δ = 173.2, 170.3, 167.9, 166.7, 166.2, 163.7, 161.7, 141.5, 138.6, 137.8, 135.1, 133.3, 132.4, 131.1, 131.0, 130.7, 130.4, 128.2, 125.9, 123.5, 123.3, 123.2, 121.3, 121.0, 120.0, 116.7, 114.8, 114.6, 114.3, 114.1, 110.2, 102.9, 97.3, 77.4, 50.3, 49.4, 49.1, 39.3, 31.4, 22.9, 22.4, 17.9. ^19^F NMR (282 MHz, DMSO-*d*_6_): δ = −59.38, −113.04. LC-MS (ESI) *m*/*z*: calcd for C_42_H_34_F_4_N_5_O_7_S^+^ [M + H]^+^: 828.21; found: 828.10. HRMS (MALDI): calcd for C_42_H_34_F_4_N_5_O_7_S [M + H]^+^: 828.21096, found: 828.2116. Purity (HPLC-UV 254 nm): 97%. *t*_R_ (method A) = 11.1 min.

#### *N*-(4-((5-(2-(2,6-Dioxopiperidin-3-yl)-1,3-dioxoisoindolin-5-yl)­pent-4-yn-1-yl)­sulfonamido)-2-(trifluoromethyl)­benzyl)-1-(3-fluorobenzyl)-1*H*-indole-5-carboxamide (**18b**)

**18b** was synthesized according to GP1 from **4** (20 mg, 0.035 mmol) and 5-bromo-2-(2,6-dioxopiperidin-3-yl)­isoindoline-1,3-dione (12 mg, 0.035 mmol) as well as CuI (1.3 mg, 0.007 mmol) and bis­(triphenylphosphine)­palladium­(II) dichloride (2.7 mg, 0.004 mmol). The product was obtained after RP flash chromatography as a colorless solid in 14% yield (4 mg, 0.005 mmol). ^1^H NMR (500 MHz, DMSO-*d*_6_): δ = 11.13 (s, 1H), 10.18 (bs, 1H), 8.89 (t, *J* = 5.8 Hz, 1H), 8.21 (d, *J* = 1.2 Hz, 1H), 7.89 (d, *J* = 7.8 Hz, 1H), 7.83 (s, 1H), 7.75 (dd, *J* = 7.8, 1.3 Hz, 1H), 7.70 (dd, *J* = 8.7, 1.6 Hz, 1H), 7.62 (d, *J* = 3.2 Hz, 1H), 7.58–7.52 (m, 2H), 7.51–7.46 (m, 2H), 7.39–7.31 (m, 1H), 7.08 (td, *J* = 8.2, 1.5 Hz, 1H), 7.03–6.97 (m, 2H), 6.63 (d, *J* = 2.8 Hz, 1H), 5.50 (s, 2H), 5.16 (dd, *J* = 12.9, 5.4 Hz, 1H), 4.58 (d, *J* = 5.3 Hz, 2H), 3.33–3.22 (m, 2H), 2.96–2.83 (m, *J* = 17.0, 13.9, 5.4 Hz, 1H), 2.65 (t, *J* = 7.0 Hz, 2H), 2.63–2.53 (m, 2H), 2.05 (dd, *J* = 6.6, 4.4 Hz, 1H), 2.01–1.94 (quint, J = 7.3 Hz, 2H). ^13^C NMR (126 MHz, DMSO-*d*_6_): δ = 173.2, 170.2, 167.9, 167.0, 166.9, 163.7, 161.7, 141.5, 141.4, 137.9, 137.8, 133.4, 132.2, 131.1, 131.0, 130.5, 130.3, 129.7, 128.2, 126.2, 125.9, 124.2, 123.5, 123.4, 121.3, 121.1, 114.8, 114.6, 114.3, 114.1, 110.2, 102.9, 94.6, 80.7, 50.4, 49.6, 49.1, 39.3, 31.4, 22.9, 22.4, 17.8 ^19^F NMR (282 MHz, DMSO-*d*_6_): δ = −59.37, −113.04. LC-MS (ESI) *m*/*z*: calcd for C_42_H_34_F_4_N_5_O_7_S^+^ [M + H]^+^: 828.21; found: 828.10. HRMS (MALDI): calcd for C_42_H_34_F_4_N_5_O_7_S^+^ [M + H]^+^: 828.21096; found: 828.2116. Purity (HPLC-UV 254 nm): 99%. *t*_R_ (method A)= 11.1 min.

#### *N*-(4-((5-(2-(2,6-Dioxopiperidin-3-yl)-1-oxoisoindolin-4-yl)­pent-4-yn-1-yl)­sulfonamido)-2-(trifluoromethyl)­benzyl)-1-(3-fluorobenzyl)-1*H*-indole-5-carboxamide (**18c**)

**18c** was synthesized according to GP1 from **4** (20 mg, 0.035 mmol) and 3-(4-bromo-1-oxoisoindolin-2-yl)­piperidine-2,6-dione (12 mg, 0.035 mmol) as well as CuI (1.3 mg, 0.007 mmol) and bis­(triphenylphosphine)­palladium­(II) dichloride (2.7 mg, 0.004 mmol). The product was obtained after RP flash chromatography as a colorless solid in 28% yield (8 mg, 0.009 mmol). ^1^H NMR (500 MHz, DMSO-d6): δ = 10.98 (s, 1H), 10.18–10.02 (m, 1H), 8.89 (t, *J* = 5.8 Hz, 1H), 8.21 (d, *J* = 1.4 Hz, 1H), 7.71–7.68 (m, 2H), 7.63 (d, *J* = 3.2 Hz, 1H), 7.55–7.43 (m, 6H), 7.38–7.32 (m, 1H), 7.11–7.05 (m, 1H), 7.03–6.98 (m, 2H), 6.63 (dd, *J* = 3.2, 0.7 Hz, 1H), 5.50 (s, 2H), 5.13 (dd, *J* = 13.3, 5.1 Hz, 1H), 4.57 (d, *J* = 5.4 Hz, 2H), 4.45–4.26 (m, 2H), 3.33–3.27 (m, 2H), 2.96–2.84 (m, 1H), 2.66–2.52 (m, 3H), 2.48–2.38 (m, 1H), 2.03–1.93 (m, 3H). ^13^C NMR (75 MHz, DMSO-d6): δ = 172.9, 170.9, 167.6, 167.4, 163.2, 161.3, 143.8, 141.0, 137.3, 134.1, 132.9, 131.9, 130.7, 130.6, 129.8, 128.6, 127.8, 127.1, 126.8, 125.5, 125.2, 123.0, 122.9, 120.9, 120.6, 118.4, 114.3, 114.2, 113.9, 113.7, 109.8, 102.5, 94.5, 77.2, 51.6, 49.9, 48.6, 46.9, 31.2, 22.6, 22.4, 17.3. ^19^F NMR (282 MHz, DMSO-*d*_6_): δ = −59.39, −113.04. LC-MS (ESI) *m*/*z*: calcd for C_42_H_36_F_4_N_5_O_6_S^+^ [M + H]^+^: 814.23; found: 814.10. HRMS (MALDI): calcd for C_42_H_35_F_4_N_5_NaO_6_S^+^ [M + Na]^+^: 836.21364; found: 836.21364. Purity (HPLC-UV 254 nm): 99%. *t*_R_ (method A) = 10.6 min.

#### *N*-(4-((5-(2-(2,6-Dioxopiperidin-3-yl)-1-oxoisoindolin-5-yl)­pent-4-yn-1-yl)­sulfonamido)-2-(trifluoromethyl)­benzyl)-1-(3-fluorobenzyl)-1*H*-indole-5-carboxamide (**18d**)

**18d** was synthesized according to GP1 from **4** (20 mg, 0.035 mmol) and 3-(5-bromo-1-oxoisoindolin-2-yl)­piperidine-2,6-dione (11 mg, 0.035 mmol) as well as CuI (1.3 mg, 0.007 mmol) and bis­(triphenylphosphine)­palladium­(II) dichloride (2.7 mg, 0.004 mmol). The product was obtained after RP flash chromatography as a colorless solid in 28% yield (8 mg, 0.009 mmol). ^1^H NMR (500 MHz, DMSO-*d*_6_): δ = 10.99 (s, 1H), 10.08 (bs, 1H), 8.91 (t, *J* = 5.6 Hz, 1H), 8.21 (s, 1H), 7.71–7.67 (m, 2H), 7.62 (d, *J* = 3.0 Hz, 1H), 7.58–7.45 (m, 5H), 7.43–7.40 (m, 1H), 7.37–7.32 (m, 1H), 7.10–7.05 (m, 1H), 7.02–6.99 (m, 2H), 6.63 (d, *J* = 2.9 Hz, 1H), 5.50 (s, 2H), 5.10 (dd, *J* = 13.2 Hz, 5.0 Hz, 1H), 4.59 (d, *J* ^3^ = 4.9 Hz, 2H), 4.37 (dd, 2H), 3.33–3.28 (m, 2H), 2.94–2.85 (m, 1H), 2.65–2.56 (m, 3H), 2.43–2.33 (m, 1H), 2.03–1.91 (m, 3H). ^13^C NMR (126 MHz, DMSO-*d*_6_): δ = 172.9, 170.9, 167.4, 163.2, 161.3, 142.4, 140.9, 137.3, 132.9, 131.1, 130.5, 129.9, 127.8, 127.1, 126.8, 126.3, 126.0, 125.5, 125.2, 123.2, 122.9, 120.9, 120.6, 116.3, 114.3, 114.1, 113.9, 113.7, 109.8, 102.5, 91.3, 81.1, 51.7, 49.9, 48.7, 47.0, 31.2, 22.6, 22.4, 17.2. LC-MS (ESI) *m*/*z*: calcd for C_42_H_36_F_4_N_5_O_6_S^+^ [M + H]^+^: 814.23; found: 814.05. HRMS (MALDI): calcd for C_42_H_35_F_4_N_5_NaO_6_S^+^ [M + Na]^+^: 836.21364; found: 836.21382. Purity (HPLC-UV 254 nm): 98%. *t*_R_ (method A) = 10.4 min.

#### *N*-(4-((5-(2-(2,6-Dioxopiperidin-3-yl)-3-oxoisoindolin-5-yl)­pent-4-yn-1-yl)­sulfonamido)-2-(trifluoromethyl)­benzyl)-1-(3-fluorobenzyl)-1*H*-indole-5-carboxamide (**18e**)

**18e** was synthesized according to GP1 from **4** (20 mg, 0.035 mmol) and 3-(6-bromo-1-oxoisoindolin-2-yl)­piperidine-2,6-dione (11 mg, 0.035 mmol) as well as CuI (1.3 mg, 0.007 mmol) and bis­(triphenylphosphine)­palladium­(II) dichloride (2.7 mg, 0.004 mmol). The product was obtained after RP flash chromatography as a colorless solid in 21% yield (6 mg, 0.007 mmol). ^1^H NMR (500 MHz, DMSO-*d*_6_): δ = 10.99 (s, 1H), 10.01 (bs, 1H), 8.88 (t, *J* = 5.6 Hz, 1H), 8.21 (s, 1H), 7.70 (d, *J* = 8.5 Hz, 1H), 7.63–7.60 (m, 2H), 7.57–7.46 (m, 6H), 7.38–7.31 (m, 1H), 7.11–7.05 (m, 1H), 7.01 (d, *J* = 8.3 Hz, 2H), 6.63 (d, *J* = 3.0 Hz, 1H), 5.50 (s, 2H), 5.09 (dd, *J* = 13.2, 5.1 Hz, 1H), 4.58 (d, *J* = 5.1 Hz, 2H), 4.35 (dd, *J* = 44.0, 11.9 Hz, 2H), 3.40–3.38 (m, 2H), 2.95–2.84 (m, 1H), 2.64–2.56 (m, 3H), 2.41–2.30 (m, 1H), 2.02–1.91 (m, 3H). ^13^C NMR (126 MHz, DMSO-*d*_6_): δ = 172.9, 170.9, 167.4, 167.3, 163.1, 161.3, 141.7, 141.0, 140.9, 137.3, 134.6, 132.9, 132.0, 130.7, 130.6, 129.8, 127.8, 125.5, 125.3, 123.9, 123.0, 122.8, 122.6, 120.9, 120.6, 114.3, 114.2, 113.9, 113.7, 109.8, 102.5, 89.7, 80.8, 51.7, 49.9, 48.6, 47.3, 31.2, 22.5, 22.4, 17.2. ^19^F NMR (282 MHz, DMSO-*d*_6_): δ = −59.38, −113.02. LC-MS (ESI) *m*/*z*: calcd for C_42_H_36_F_4_N_5_O_6_S^+^ [M + H]^+^: 814.23; found: 814.10. HRMS (MALDI): calcd for C_42_H_35_F_4_N_5_NaO_6_S^+^ [M + Na]^+^: 836.21364; found: 836.21349. Purity (HPLC-UV 254 nm): 97%. *t*_R_ (method A) = 10.5 min.

#### *N*-(4-(Cyclopropanesulfonamido)-2-(trifluoromethyl)­benzyl)-1-((5-(2-(2,6-dioxopiperidin-3-yl)-1,3-dioxoisoindolin-4-yl)­pent-4-yn-1-yl)­sulfonyl)-1*H*-indole-5-carboxamide (**19a**)

**19a** was synthesized according to GP1 from **5** (40 mg, 0.07 mmol) and 4-bromo-2-(2,6-dioxopiperidin-3-yl)­isoindoline-1,3-dione (24 mg, 0.07 mmol) as well as CuI (2.6 mg, 0.014 mmol) and bis­(triphenylphosphine)­palladium­(II) dichloride (5.4 mg, 0.007 mmol). The product was obtained after RP flash chromatography and subsequent preparative HPLC (method A) as a colorless solid in 8% yield (4 mg, 0.006 mmol). ^1^H NMR (500 MHz, DMSO-*d*_6_): δ = 11.17 (s, 1H), 10.07 (s, 1H), 9.05 (t, *J* = 5.8 Hz, 1H), 8.25 (d, *J* = 1.0 Hz, 1H), 7.92 (d, *J* = 8.7 Hz, 1H), 7.88 (dd, *J* = 8.7, 1.6 Hz, 1H), 7.85–7.82 (m, 1H), 7.78 (t, *J* = 7.6 Hz, 1H), 7.73 (d, *J* = 3.6 Hz, 1H), 7.69 (dd, *J* = 7.7, 0.8 Hz, 1H), 7.57 (s, 1H), 7.53–7.47 (m, 2H), 6.94 (d, *J* = 3.6 Hz, 1H), 5.15 (dd, *J* = 12.9, 5.4 Hz, 1H), 4.61 (d, *J* = 5.4 Hz, 2H), 4.00–3.94 (m, 2H), 2.98–2.86 (m, 1H), 2.73–2.53 (m, 5H), 2.12–2.02 (m, 1H), 1.90–1.79 (m, 2H), 0.98–0.91 (m, 4H). ^13^C NMR (126 MHz, DMSO-*d*_6_): δ = 172.8, 169.8, 166.4, 166.2, 165.9, 138.0, 137.6, 136.0, 134.6, 132.6, 131.9, 130.3, 129.9, 129.8, 129.2, 128.3, 127.0, 126.8, 123.8, 123.5, 122.9, 121.1, 119.2, 118.1, 116.9, 112.7, 108.4, 96.1, 77.3, 52.2, 48.9, 30.9, 29.4, 22.1, 21.9, 17.2, 5.1, 1.2. ^19^F NMR (471 MHz, DMSO-*d*_6_): δ = −59.31. LC-MS (ESI) *m*/*z*: calcd for C_38_H_33_F_3_N_5_O_9_S_2_^+^ [M + H]^+^: 824.16; found: 824.30. HRMS (MALDI): calcd for C_38_H_32_F_3_N_5_NaO_9_S_2_^+^ [M + Na]^+^: 846.14857; found: 846.14862. Purity (HPLC-UV 254 nm): 99%. *t*_R_ (method A) = 9.8 min.

#### *N*-(4-(Cyclopropanesulfonamido)-2-(trifluoromethyl)­benzyl)-1-((5-(2-(2,6-dioxopiperidin-3-yl)-1,3-dioxoisoindolin-5-yl)­pent-4-yn-1-yl)­sulfonyl)-1*H*-indole-5-carboxamide (**19b**)

**19b** was synthesized according to GP1 from **5** (45 mg, 0.079 mmol) and 5-bromo-2-(2,6-dioxopiperidin-3-yl)­isoindoline-1,3-dione (27 mg, 0.079 mmol) as well as CuI (3.0 mg, 0.016 mmol) and bis­(triphenylphosphine)­palladium­(II) dichloride (5.6 mg, 0.008 mmol). The product was obtained after RP flash chromatography and subsequent preparative HPLC (method A) as a colorless solid in 32% yield (21 mg, 0.025 mmol). ^1^H NMR (500 MHz, DMSO-*d*_6_): δ = 11.10 (s, 1H), 10.06 (s, 1H), 9.08 (t, *J* = 5.8 Hz, 1H), 8.27 (d, *J* = 1.1 Hz, 1H), 7.95 (d, *J* = 8.7 Hz, 1H), 7.90 (dd, *J* = 8.7, 1.6 Hz, 1H), 7.86–7.83 (m, 2H), 7.76 (d, *J* = 3.7 Hz, 1H), 7.71 (dd, *J* = 7.9, 1.2 Hz, 1H), 7.56 (d, *J* = 1.7 Hz, 1H), 7.52–7.46 (m, 2H), 6.97 (dd, *J* = 3.7, 0.6 Hz, 1H), 5.15 (dd, *J* = 12.9, 5.4 Hz, 1H), 4.61 (d, *J* = 5.4 Hz, 2H), 3.89–3.85 (m, 2H), 2.94–2.82 (m, 1H), 2.67 (tt, *J* = 6.7, 5.5 Hz, 1H), 2.63–2.52 (m, 3H), 2.12–2.08 (m, 1H), 2.07–2.01 (m, 1H), 1.85–1.74 (m, 2H), 0.97–0.91 (m, 4H). ^13^C NMR (126 MHz, DMSO-*d*_6_): δ = 172.8, 169.8, 166.6, 166.5, 166.4, 137.6, 137.4, 136.1, 132.6, 131.7, 130.0, 129.8, 129.2, 128.9, 128.4, 127.0, 125.9, 125.2, 123.9, 123.6, 123.5, 123.0, 121.2, 118.1, 116.9, 112.6, 108.4, 93.4, 80.4, 52.3, 48.5, 30.9, 29.7, 22.1, 21.9, 17.1, 5.1, 1.2. ^19^F NMR (471 MHz, DMSO-*d*_6_): δ = −59.34. LC-MS (ESI) *m*/*z*: calcd for C_38_H_33_F_3_N_5_O_9_S_2_^+^ [M + H]^+^: 824.16; found: 824.35. HRMS (ESI): calcd for C_38_H_31_F_3_N_5_O_9_S_2_^–^ [M – H]^−^: 822.1521; found: 822.1534. Purity (HPLC-UV 254 nm): 99%. *t*_R_ (method A) = 9.8 min.

#### *N*-(4-(Cyclopropanesulfonamido)-2-(trifluoromethyl)­benzyl)-1-((5-(2-(2,6-dioxopiperidin-3-yl)-1-oxoisoindolin-4-yl)­pent-4-yn-1-yl)­sulfonyl)-1*H*-indole-5-carboxamide (**19c**)

**19c** was synthesized according to GP1 from **5** (40 mg, 0.070 mmol) and 3-(4-bromo-1-oxoisoindolin-2-yl)­piperidine-2,6-dione (24 mg, 0.070 mmol) as well as CuI (3.0 mg, 0.016 mmol) and bis­(triphenylphosphine)­palladium­(II) dichloride (5.6 mg, 0.008 mmol). The product was obtained after RP flash chromatography and subsequent preparative HPLC (method A) as a colorless solid in 8% yield (5 mg, 0.006 mmol). ^1^H NMR (500 MHz, DMSO-*d*_6_): δ = 11.01 (s, 1H), 10.08 (s, 1H), 9.08 (t, *J* = 5.8 Hz, 1H), 8.28 (s, 1H), 7.96–7.89 (m, 2H), 7.75 (d, *J* = 3.7 Hz, 1H), 7.69 (dd, *J* = 7.0, 1.6 Hz, 1H), 7.57 (d, *J* = 1.9 Hz, 1H), 7.54–7.43 (m, 4H), 6.96 (d, *J* = 3.6 Hz, 1H), 5.14 (dd, *J* = 13.3, 5.1 Hz, 1H), 4.63 (d, *J* = 5.4 Hz, 2H), 4.43–4.22 (m, 2H), 3.85–3.79 (m, 2H), 2.98–2.87 (m, 1H), 2.72–2.57 (m, 2H), 2.54 (t, *J* = 7.1 Hz, 2H), 2.44–2.35 (m, 1H), 2.05–1.97 (m, 1H), 1.86–1.77 (m, 2H), 0.96–0.93 (m, 4H). ^13^C NMR (126 MHz, DMSO-*d*_6_): δ = 172.9, 171.0, 167.6, 166.5, 143.8, 137.6, 136.1, 134.2, 132.6, 131.9, 129.9, 129.8, 129.3, 128.5, 128.3, 127.1, 126.9, 125.2, 123.9, 123.5, 122.9, 121.2, 118.2, 116.9, 112.6, 108.5, 93.8, 77.4, 52.4, 51.6, 46.9, 31.2, 29.7, 29.0, 22.3, 17.1, 5.1, 1.2. ^19^F NMR (471 MHz, DMSO-*d*_6_): δ = −59.34. LC-MS (ESI) *m*/*z*: calcd for C_38_H_35_F_3_N_5_O_8_S_2_^+^ [M + H]^+^: 810.18; found: 810.40. HRMS (ESI): calcd for C_38_H_33_F_3_N_5_O_8_S_2_^–^ [M – H]^−^: 808.1728; found: 808.1743. Purity (HPLC-UV 254 nm): 99%. *t*_R_ (method A) = 9.3 min.

#### *N*-(4-(Cyclopropanesulfonamido)-2-(trifluoromethyl)­benzyl)-1-((5-(2-(2,6-dioxopiperidin-3-yl)-1-oxoisoindolin-5-yl)­pent-4-yn-1-yl)­sulfonyl)-1*H*-indole-5-carboxamide (**19d**)

**19d** was synthesized according to GP1 from **5** (30 mg, 0.053 mmol) and 3-(5-bromo-1-oxoisoindolin-2-yl)­piperidine-2,6-dione (17 mg, 0.053 mmol) as well as CuI (2.0 mg, 0.011 mmol) and bis­(triphenylphosphine)­palladium­(II) dichloride (3.8 mg, 0.005 mmol). The product was obtained after RP flash chromatography and subsequent preparative HPLC (method A) as a colorless solid in 15% yield (6 mg, 0.008 mmol). ^1^H NMR (500 MHz, DMSO-*d*_6_): δ = 10.88 (s, 1H), 9.13 (t, *J* = 5.8 Hz, 1H), 8.31 (t, *J* = 1.1 Hz, 1H), 7.95 (d, *J* = 0.9 Hz, 2H), 7.76 (d, *J* = 3.7 Hz, 1H), 7.66 (d, *J* = 7.8 Hz, 1H), 7.57 (d, *J* = 2.0 Hz, 1H), 7.53–7.46 (m, 3H), 7.40–7.37 (m, 1H), 6.98 (d, *J* = 3.7 Hz, 1H), 5.10 (dd, *J* = 13.3, 5.1 Hz, 1H), 4.63 (d, *J* = 5.4 Hz, 2H), 4.36 (dd, *J* = 63.5, 17.6 Hz, 2H), 3.86–3.78 (m, 2H), 2.95–2.86 (m, 1H), 2.67 (tt, *J* = 7.1, 5.7 Hz, 1H), 2.62–2.56 (m, 1H), 2.54–2.51 (m, 2H), 2.42–2.31 (m, 1H), 2.02–1.95 (m, 1H), 1.82–1.75 (m, 2H), 0.96–0.92 (m, 4H). * 1 × NH was not detected in this ^1^H NMR experiment. ^13^C NMR (126 MHz, DMSO-*d*_6_): δ = 172.9, 170.9, 167.4, 166.5, 142.3, 137.6, 136.1, 132.6, 131.2, 131.1, 129.9, 129.8, 129.3, 128.4, 127.0, 126.8, 126.4, 125.8, 123.9, 123.5, 123.1, 123.0, 121.3, 116.9, 116.8, 112.6, 108.5, 90.7, 81.3, 52.4, 51.6, 46.9, 31.2, 29.7, 22.4, 16.9, 5.1, 1.2. ^19^F NMR (471 MHz, DMSO-*d*_6_): δ = −59.34. LC-MS (ESI) *m*/*z*: calcd for C_38_H_35_F_3_N_5_O_8_S_2_^+^ [M + H]^+^: 810.18; found: 810.35. HRMS (ESI): calcd for C_38_H_33_F_3_N_5_O_8_S_2_^–^ [M – H]^−^: 808.1728; found: 808.1744. Purity (HPLC-UV 254 nm): 99%. *t*_R_ (method A) = 9.0 min.

#### *N*-(4-(Cyclopropanesulfonamido)-2-(trifluoromethyl)­benzyl)-1-((5-(2-(2,6-dioxopiperidin-3-yl)-3-oxoisoindolin-5-yl)­pent-4-yn-1-yl)­sulfonyl)-1*H*-indole-5-carboxamide (**19e**)

**19e** was synthesized according to GP1 from **5** (30 mg, 0.053 mmol) and 3-(6-bromo-1-oxoisoindolin-2-yl)­piperidine-2,6-dione (17 mg, 0.053 mmol) as well as CuI (2.0 mg, 0.011 mmol) and bis­(triphenylphosphine)­palladium­(II) dichloride (3.8 mg, 0.005 mmol). The product was obtained after RP flash chromatography and subsequent preparative HPLC (method A) as a colorless solid in 11% yield (5 mg, 0.006 mmol). ^1^H NMR (500 MHz, DMSO-*d*_6_): δ = 11.00 (s, 1H), 10.07 (s, 1H), 9.11 (t, *J* = 5.8 Hz, 1H), 8.30 (d, *J* = 1.3 Hz, 1H), 7.98–7.91 (m, 2H), 7.77 (d, *J* = 3.7 Hz, 1H), 7.66 (s, 1H), 7.58–7.54 (m, 2H), 7.54–7.46 (m, 3H), 6.98 (d, *J* = 3.7 Hz, 1H), 5.10 (dd, *J* = 13.3, 5.1 Hz, 1H), 4.63 (d, *J* = 5.4 Hz, 2H), 4.39 (dd, *J* = 61.5, 17.8 Hz, 2H), 3.88–3.80 (m, 2H), 2.95–2.82 (m, 1H), 2.71–2.62 (m, 1H), 2.62–2.56 (m, 1H), 2.53–2.51 (m, 2H), 2.43–2.32 (m, 1H), 2.00 (dtd, *J* = 7.4, 5.2, 2.2 Hz, 1H), 1.82–1.74 (m, 2H), 0.97–0.93 (m, 4H). ^13^C NMR (126 MHz, DMSO-*d*_6_): δ = 172.9, 170.9, 167.3, 166.5, 141.7, 137.6, 136.1, 134.6, 132.6, 132.0, 129.8, 129.3, 128.4, 127.0, 126.8, 125.6, 123.9, 123.9, 123.5, 123.1, 122.4, 121.3, 116.9, 116.8, 112.6, 108.5, 89.1, 80.9, 52.5, 51.7, 47.3, 31.2, 29.7, 22.4, 22.4, 16.9, 5.1, 1.2.^19^F NMR (471 MHz, DMSO-*d*_6_): δ −59.32. LC-MS (ESI) *m*/*z*: calcd for C_38_H_35_F_3_N_5_O_8_S_2_^+^ [M + H]^+^: 810.18; found: 810.40. HRMS (ESI) *m*/*z*: calcd for C_38_H_33_F_3_N_5_O_8_S_2_^–^ [M – H]^−^: 808.1728; found: 808.1741. Purity (HPLC-UV 254 nm): 99%. *t*_R_ (method A) = 9.5 min.

#### *N*-(4-((3-(1-(2-(2-((2-(2,6-Dioxopiperidin-3-yl)-1,3-dioxoisoindolin-4-yl)­amino)-2-oxoethoxy)­ethyl)-1*H*-1,2,3-triazol-4-yl)­propyl)­sulfonamido)-2-(trifluoromethyl)­benzyl)-1-(3-fluorobenzyl)-1*H*-indole-5-carboxamide (**21a**)

**21a** was synthesized according to GP2 from **4** (9.3 mg, 0.016 mmol) and pomalidomide-PEG1-azide (**20a**) (7.5 mg, 0.018 mmol) as well as CuSO_4_·5H_2_O (1.2 mg, 0.005 mmol) and sodium ascorbate (0.9 mg, 0.005 mmol). The analytical pure product was obtained after preparative HPLC (method A) as a colorless solid in 79% yield (12.4 mg, 0.013 mmol). ^1^H NMR (300 MHz, DMSO-*d*_6_): δ = 8.90 (s, 1H), 8.68 (d, *J* = 8.3 Hz, 1H), 8.22 (s, 1H), 7.90 (s, 1H), 7.83 (t, *J* = 7.8 Hz, 1H), 7.71 (d, *J* = 9.0 Hz, 1H), 7.61 (d, *J* = 6.7 Hz, 2H), 7.50 (t, *J* = 10.5 Hz, 3H), 7.45–7.21 (m, 2H), 7.15–6.90 (m, 3H), 6.63 (d, *J* = 2.9 Hz, 1H), 5.49 (s, 2H), 5.16 (dd, *J* = 12.7, 5.4 Hz, 1H), 4.60 (s, 4H), 4.08–3.93 (m, 4H), 3.16 (t, *J* = 7.9 Hz, 2H), 2.97–2.80 (m, *J* = 12.2 Hz, 1H), 2.71 (t, *J* = 7.5 Hz, 2H), 2.66–2.52 (m, 2H), 2.19–2.03 (m, *J* = 13.7 Hz, 1H), 2.04–1.90 (m, *J* = 7.2 Hz, 2H). * 3 × NH signals were not detectable in this ^1^H experiment. ^13^C NMR (75 MHz, DMSO-*d*_6_): δ = 172.7, 169.7, 168.7, 168.4, 167.7, 167.6, 166.8, 164.4, 161.7, 145.6, 141.3, 137.4, 136.5, 135.9, 133.2, 131.4, 130.8, 130.6, 129.8, 127.9, 126.1, 124.9, 123.1, 122.9, 120.9, 120.7, 118.48, 117.1, 114.4, 114.2, 113.9, 113.7, 109.8, 102.5, 69.8, 50.5, 49.1, 48.8, 48.01, 47.6, 47.4, 47.2, 31.0, 23.2, 22.0. ^19^F NMR (282 MHz, DMSO-*d*_6_): δ = −59.36, −113.03. LC-MS (ESI) *m*/*z*: calcd for C_46_H_42_F_4_N_9_O_9_S^+^ [M + H]^+^: 972.27; found: 972.20. HRMS (MALDI) *m*/*z*: calcd for C_46_H_42_F_4_N_9_O_9_S^+^ [M + H]^+^: 972.27568; found: 972.2755. Purity (HPLC-UV 254 nm): 99%. *t*_R_ (method A) = 10.2 min.

#### *N*-(4-((3-(1-(2-(2-(2-((2-(2,6-Dioxopiperidin-3-yl)-1,3-dioxoisoindolin-4-yl)­amino)-2-oxoethoxy)­ethoxy)­ethyl)-1*H*-1,2,3-triazol-4-yl)­propyl)­sulfonamido)-2-(trifluoromethyl)­benzyl)-1-(3-fluorobenzyl)-1*H*-indole-5-carboxamide (**21b**)

**21b** was synthesized according to GP2 from **4** (10 mg, 0.018 mmol) and pomalidomide-PEG2-azide (**20b**) (8.6 mg, 0.019 mmol) as well as CuSO_4_·5H_2_O (1.3 mg, 0.005 mmol) and sodium ascorbate (1.0 mg, 0.005 mmol). The analytical pure product was obtained after preparative HPLC (method A) as a colorless solid in 61% yield (10.9 mg, 0.011 mmol). ^1^H NMR (500 MHz, DMSO-*d*_6_) δ = 11.15 (s, 1H), 10.32 (s, 1H), 10.13 (s, 1H), 8.93 (t, *J* = 5.8 Hz, 1H), 8.71 (d, *J* = 8.4 Hz, 1H), 8.22 (s, 1H), 7.86–7.81 (m, 1H), 7.77 (s, 1H), 7.71 (d, *J* = 7.7 Hz, 1H), 7.62 (t, *J* = 5.7 Hz, 2H), 7.56–7.52 (m, 1H), 7.53–7.47 (m, 2H), 7.41 (d, *J* = 8.5 Hz, 1H), 7.38–7.31 (m, 1H), 7.11–6.99 (m, 3H), 6.63 (d, *J* = 3.0 Hz, 1H), 5.50 (s, 2H), 5.15 (dd, *J* = 12.9, *J* = 5.4 Hz, 1H), 4.60 (d, *J* = 5.3 Hz, 2H), 4.43 (t, *J* = 5.2 Hz, 2H), 4.13 (s, 2H), 3.80 (t, *J* = 5.2 Hz, 2H), 3.70 (dd, *J* = 5.8, *J* = 3.2 Hz, 2H), 3.63 (dd, *J* = 5.6, *J* = 3.2 Hz, 2H), 3.19–3.14 (m, 2H), 2.94–2.83 (m, 1H), 2.66 (t, *J* = 7.5 Hz, 2H), 2.00–1.93 (m, 2H), 0.90–0.85 (m, 3H). ^13^C NMR (126 MHz, DMSO-*d*_6_) δ = 206.9, 173.2, 170.20, 169.7, 168.7, 167.8, 167.1, 163.6, 161.6, 145.7, 141.3, 141.40, 137.7, 136.9, 136.4, 133.3, 131.8, 131.1, 130.2, 128.2, 125.9, 124.8, 123.4, 123.4, 123.3, 122.9, 121.3, 121.0, 118.8, 116.5, 114.8, 114.6, 114.3, 114.1, 110.2, 102.9, 71.0, 70.6, 69.9, 69.3, 51.2, 49.8, 49.6, 48.9, 31.2, 23.6, 14.4, 11.2. ^19^F NMR (282 MHz, DMSO-*d*_6_) δ = −59.34, −113.03. LC-MS (ESI) *m*/*z*: calcd for C_48_H_46_F_4_N_9_O_10_S^+^ [M + H]^+^: 1016.30; found: 1016.40. HRMS (MALDI) *m*/*z*: calcd for C_48_H_46_F_4_N_9_O_10_S^+^ [M + H]^+^: 1016.30190; found: 1016.30119. Purity (HPLC-UV 254 nm): 98%. *t*_R_ (method A) = 10.3 min.

#### *N*-(4-((3-(1-(2-(2-(2-(2-((2-(2,6-Dioxopiperidin-3-yl)-1,3-dioxoisoindolin-4-yl)­amino)-2-oxoethoxy)­ethoxy)­ethoxy)­ethyl)-1*H*-1,2,3-triazol-4-yl)­propyl)­sulfonamido)-2-(trifluoromethyl)­benzyl)-1-(3-fluorobenzyl)-1*H*-indole-5-carboxamide (**21c**)

**21c** was synthesized according to GP2 from **4** (9.7 mg, 0.017 mmol) and pomalidomide-PEG3-azide (**20c**) (9.6 mg, 0.019 mmol) as well as CuSO_4_·5H_2_O (1.3 mg, 0.005 mmol) and sodium ascorbate (1.0 mg, 0.005 mmol). The analytical pure product was obtained after preparative HPLC (method A) as a colorless solid in 84% yield (15.0 mg, 0.014 mmol). ^1^H NMR (300 MHz, DMSO-*d*_6_): δ = 11.16 (s, 1H), 10.34 (s, 1H), 10.14 (s, 1H), 8.91 (t, *J* = 5.5 Hz, 1H), 8.71 (d, *J* = 8.4 Hz, 1H), 8.23 (d, *J* = 1.2 Hz, 1H), 7.90–7.79 (m, 1H), 7.77 (s, 1H), 7.75–7.69 (m, 1H), 7.64–7.59 (m, 2H), 7.58–7.47 (m, 3H), 7.45–7.38 (m, 1H), 7.37–7.29 (m, 1H), 7.13–6.97 (m, 3H), 6.64 (d, *J* = 3.1 Hz, 1H), 5.50 (s, 2H), 5.16 (dd, *J* = 12.9, 5.2 Hz, 1H), 4.61 (s, 2H), 4.40 (t, *J* = 5.3 Hz, 2H), 4.18 (s, 2H), 3.72 (t, *J* = 4.6 Hz, 4H), 3.62 (dd, *J* = 5.8, 3.3 Hz, 2H), 3.453–3.45 (m, 4H), 3.24–3.15 (m, 2H), 2.88 (dd, *J* = 21.4, 9.9 Hz, 1H), 2.71 (t, *J* = 7.5 Hz, 2H), 2.66–2.54 (m, 2H), 2.15–2.03 (m, *J* = 6.3 Hz, 1H), 2.03–1.91 (m, 2H).^13^C NMR (75 MHz, DMSO-*d*_6_): δ = 172.7, 169.7, 169.3, 168.2, 167.4, 167.3, 166.7, 163.8, 161.1, 145.3, 141.0, 140.9, 137.3, 136.5, 135.9, 133.3, 131.3, 130.6, 130.5, 129.7, 127.7, 125.7, 124.8, 122.9, 122.8, 122.4, 120.9, 120.6, 118.3, 118.2, 116.1, 114.3, 114.1, 113.9, 113.6, 109.7, 102.4, 70.8, 70.2, 69.6, 69.5, 68.7, 50.4, 49.2 48.9, 48.6, 30.9, 23.2, 21.9. ^19^F NMR (376 MHz, DMSO-*d*_6_) δ = −59.39, −113.05. LC-MS (ESI) *m*/*z*: calcd for C_50_H_50_F_4_N_9_O_11_S^+^ [M + H]^+^: 1060.32; found: 1060.30. HRMS (MALDI) *m*/*z*: calcd for C_50_H_50_F_4_N_9_O_11_S^+^ [M + H]^+^ 1060.32811; found: 1060.32810. Purity (HPLC-UV 254 nm): 100%. *t*_R_ (method A) = 10.2 min.

#### *N*-(4-((3-(1-(14-((2-(2,6-Dioxopiperidin-3-yl)-1,3-dioxoisoindolin-4-yl)­amino)-14-oxo-3,6,9,12-tetraoxatetradecyl)-1*H*-1,2,3-triazol-4-yl)­propyl)­sulfonamido)-2-(trifluoromethyl)­benzyl)-1-(3-fluorobenzyl)-1*H*-indole-5-carboxamide (**21d**)

**21d** was synthesized according to GP2 from **4** (3.3 mg, 0.0057 mmol) and pomalidomide-PEG4-azide (**20d**) (3.4 mg, 0.0064 mmol) as well as CuSO_4_·5H_2_O (0.44 mg, 0.0017 mmol) and sodium ascorbate (0.35 mg, 0.0017 mmol). The analytical pure product was obtained after preparative HPLC (method A) as a colorless solid in 69% yield (4.4 mg, 0.004 mmol). ^1^H NMR (400 MHz, DMSO-*d*_6_): δ = 11.15 (s, 1H), 10.34 (s, 1H), 8.91 (t, *J* = 5.8 Hz, 1H), 8.71 (d, *J* = 8.4 Hz, 1H), 8.22 (s, 1H), 7.85 (t, *J* = 7.9 Hz, 1H), 7.77 (s, 1H), 7.70 (d, *J* = 8.7 Hz, 1H), 7.65–7.58 (m, 2H), 7.58–7.44 (m, 3H), 7.42–7.29 (m, 2H), 7.08 (dd, *J* = 10.2, 8.0 Hz, 1H), 7.01 (d, *J* = 8.6 Hz, 2H), 6.63 (d, *J* = 3.1 Hz, 1H), 5.49 (s, 2H), 5.15 (dd, *J* = 12.7, 5.3 Hz, 1H), 4.60 (d, *J* = 5.2 Hz, 2H), 4.40 (t, *J* = 5.2 Hz, 2H), 4.18 (s, 2H), 3.72 (dd, *J* = 10.3, 5.6 Hz, 4H), 3.68–3.61 (m, 2H), 3.53–3.47 (m, 3H), 3.47–3.40 (m, 5H), 3.22–3.12 (m, 2H), 2.95–2.83 (m, 1H), 2.70 (t, *J* = 7.5 Hz, 2H), 2.64–2.52 (m, 2H), 2.12–2.02 (m, 1H), 2.03–1.92 (m, 2H). *1 × NH was not detected in this 1H experiment. ^13^C NMR (101 MHz, DMSO-*d*_6_) δ = 172.8, 169.8, 169.4, 168.2, 167.4, 166.7, 163.2, 161.16, 145.3, 141.0, 140.9, 137.3, 136.6, 135.9, 132.9, 131.3, 130.7, 130.6, 129.7, 127.8, 125.5, 124.4, 123.0, 122.8, 122.5, 120.9, 120.6, 118.35, 116.5, 116.42, 116.1, 114.4, 114.2, 113.9, 113.7, 109.8, 102.5, 70.8, 70.2, 69.8, 69.7, 69.6, 69.5, 68.7, 50.4, 49.2, 48.9, 48.6, 30.9, 23.2, 21.9. ^19^F NMR (376 MHz, DMSO-*d*_6_) δ = −59.36, −113.03. LC-MS (ESI) *m*/*z*: calcd for C_52_H_53_F_4_N_9_NaO_12_S^+^ [M + Na]^+^: 1126.34; found: 1126.10. HRMS (ESI) *m*/*z*: calcd for C_52_H_53_F_4_N_9_O_12_S^–^ [M – H]^−^ 1102.3398; found: 1102.3384. Purity (HPLC-UV 254 nm): 100%. *t*_R_ (method A) = 10.1 min.

#### *N*-(4-((3-(1-(17-((2-(2,6-Dioxopiperidin-3-yl)-1,3-dioxoisoindolin-4-yl)­amino)-17-oxo-3,6,9,12,15-pentaoxaheptadecyl)-1*H*-1,2,3-triazol-4-yl)­propyl)­sulfonamido)-2-(trifluoromethyl)­benzyl)-1-(3-fluorobenzyl)-1*H*-indole-5-carboxamide (**21e**)

**21e** was synthesized according to GP2 from **4** (3.3 mg, 0.0057 mmol) and pomalidomide-PEG5-azide (**20e**) (3.7 mg, 0.0064 mmol) as well as CuSO_4_·5H_2_O (0.44 mg, 0.0017 mmol) and sodium ascorbate (0.35 mg, 0.0017 mmol). The analytical pure product was obtained after preparative HPLC (method A) as a colorless solid in 72% yield (4.8 mg, 0.004 mmol). ^1^H NMR (400 MHz, DMSO-*d*_6_): δ = 11.14 (s, 1H), 10.34 (s, 1H), 10.12 (s, 1H), 8.91 (t, *J* = 5.7 Hz, 1H), 8.71 (d, *J* = 8.4 Hz, 1H), 8.22 (d, *J* = 1.0 Hz, 1H), 7.89–7.81 (m, 1H), 7.77 (s, 1H), 7.70 (dd, *J* = 8.7, 1.4 Hz, 1H), 7.65–7.60 (m, 2H), 7.56–7.45 (m, 3H), 7.43–7.30 (m, 2H), 7.13–7.04 (m, 1H), 7.04–6.95 (m, *J* = 8.9 Hz, 2H), 6.63 (d, *J* = 3.1 Hz, 1H), 5.49 (s, 2H), 5.15 (dd, *J* = 12.8, 5.4 Hz, 1H), 4.60 (d, *J* = 5.2 Hz, 2H), 4.41 (t, *J* = 5.2 Hz, 2H), 4.19 (s, 2H), 3.73 (dd, *J* = 10.0, 5.0 Hz, 4H), 3.70 −3.60 (m, 2H), 3.53–3.41 (m, 12H), 3.22–3.14 (m, 2H), 2.98–2.82 (m, 1H), 2.70 (t, *J* = 7.4 Hz, 2H), 2.57 (dd, *J* = 23.1, 11.2 Hz, 2H), 2.11–2.03 (m, 1H), 2.03–1.93 (m, 2H). ^13^C NMR (101 MHz, DMSO-*d*_6_) δ = 172.8, 169.8, 169.4, 168.2, 167.4, 166.7, 163.7,161.0, 145.3, 141.1, 140.9,137.5, 137.3, 136.5, 135.9, 131.3, 130.7, 130.6, 129.8, 127.8, 125.5, 124.4, 123.0, 122.8, 122.5, 120.9, 120.6, 118.4, 116.1, 114.4, 114.2, 113.9, 113.7, 109.79, 102.5, 70.8, 70.2, 69.9, 69.7, 69.65, 69.6, 69.5, 69.4, 68.7, 50.4, 49.2, 48.9, 48.6, 30.9, 29.3, 23.2, 23.2, 21.9. ^19^F NMR (377 MHz, DMSO-*d*_6_): δ = −59.37, −113.03. LC-MS (ESI) *m*/*z*: calcd for C_54_H_58_F_4_N_9_O_13_S^+^ [M + H]^+^: 1148.38; found: 1148.55. HRMS (ESI): calcd for C_54_H_57_F_4_N_9_NaO_13_S^+^ [M + Na]^+^:1170.3626; found: 1170.3622. Purity (HPLC-UV 254 nm): 100%. *t*_R_ (method A) = 10.1 min.

#### *N*-(4-((3-(1-(20-((2-(2,6-Dioxopiperidin-3-yl)-1,3-dioxoisoindolin-4-yl)­amino)-20-oxo-3,6,9,12,15,18-hexaoxaicosyl)-1*H*-1,2,3-triazol-4-yl)­propyl)­sulfonamido)-2-(trifluoromethyl)­benzyl)-1-(3-fluorobenzyl)-1*H*-indole-5-carboxamide (**21f**)

**21f** was synthesized according to GP2 from **4** (10.0 mg, 0.018 mmol) and pomalidomide-PEG6-azide (**20f**) (12.6 mg, 0.019 mmol) as well as CuSO_4_·5H_2_O (1.3 mg, 0.0053 mmol) and sodium ascorbate (1.1 mg, 0.0053 mmol). The analytical pure product was obtained after preparative HPLC (method A) as a colorless solid in 36% yield (7.4 mg, 0.006 mmol). ^1^H NMR (400 MHz, DMSO-*d*_6_): δ = 11.15 (s, 1H), 10.35 (s, 1H), 8.91 (t, *J* = 5.9 Hz, 1H), 8.71 (d, *J* = 8.4 Hz, 1H), 8.22 (d, *J* = 1.4 Hz, 1H), 7.88–7.82 (m, 1H), 7.77 (s, 1H), 7.70 (dd, *J* = 8.7, 1.6 Hz, 1H), 7.64–7.60 (m, 2H), 7.57–7.45 (m, 3H), 7.43–7.30 (m, 2H), 7.12–7.04 (m, 1H), 7.01 (d, *J* = 9.3 Hz, 2H), 6.63 (d, *J* = 3.2 Hz, 1H), 5.49 (s, 2H), 5.15 (dd, *J* = 12.8, 5.5 Hz, 1H), 4.60 (d, *J* = 5.3 Hz, 2H), 4.41 (t, *J* = 5.3 Hz, 2H), 4.19 (s, 2H), 3.77–3.70 (m, 4H), 3.67–3.62 (m, 2H), 3.54–3.50 (m, 2H), 3.49–3.41 (m, 14H), 3.22–3.14 (m, 2H), 2.95–2.83 (m, 1H), 2.70 (t, *J* = 7.5 Hz, 2H), 2.65–2.53 (m, 2H), 2.12–2.03 (m, 1H), 2.03–1.92 (m, 2H). *1 × NH was not detected in this ^1^H NMR experiment. ^13^C NMR (75 MHz, DMSO-*d*_6_): δ = 172.7, 169.7, 169.4, 168.2, 167.3, 166.6, 163.0, 161.4, 145.3, 141.0, 140.9, 137.3, 136.5, 135.9, 131.3, 130.6, 130.5, 129.7, 127.7, 125.5, 125.0, 124.4, 123.2, 123.0, 122.9, 122.8, 122.4, 120.9, 120.6, 118.3, 116.1, 114.3, 114.1, 113.8, 113.7, 109.7, 102.4, 70.8, 70.2, 69.9, 69.8, 69.7, 69.6, 69.5, 68.7, 50.4, 49.2, 48.9, 48.6, 30.9, 23.3, 23.2, 21.9. ^19^F NMR (377 MHz, DMSO-*d*_6_): δ = −59.38, −113.03. LC-MS (ESI) *m*/*z*: calcd for C_56_H_62_F_4_N_9_O_14_S^+^ [M + H]^+^: 1192.40; found: 1192.55. HRMS (ESI) *m*/*z*: calcd for C_56_H_61_F_4_N_9_NaO_14_S^+^ [M + Na]^+^1214.3887; found: 1214.3887. Purity (HPLC-UV 254 nm): 100%. *t*_R_ (method A) = 10.1 min).

#### *N*-(4-((3-(1-(1-((2-(2,6-Dioxopiperidin-3-yl)-1,3-dioxoisoindolin-4-yl)­oxy)-2-oxo-6,9,12,15-tetraoxa-3-azaheptadecan-17-yl)-1*H*-1,2,3-triazol-4-yl)­propyl)­sulfonamido)-2-(trifluoromethyl)­benzyl)-1-(3-fluorobenzyl)-1*H*-indole-5-carboxamide (**23**)

**23** was synthesized according to GP2 from **4** (10.0 mg, 0.018 mmol) and thalidomide-O-PEG4-azide (**25**) (10.1 mg, 0.019 mmol) as well as CuSO_4_·5H_2_O (1.3 mg, 0.0053 mmol) and sodium ascorbate (1.1 mg, 0.0053 mmol). The analytical pure product was obtained after preparative HPLC (method A) as a colorless solid in 42% yield (8.5 mg, 0.007 mmol). ^1^H NMR (400 MHz, DMSO-d6): δ = 11.11 (s, 1H), 8.91 (t, *J* = 5.8 Hz, 1H), 8.22 (d, *J* = 1.4 Hz, 1H), 8.00 (t, *J* = 5.5 Hz, 1H), 7.83–7.76 (m, 2H), 7.71 (dd, *J* = 8.7, 1.6 Hz, 1H), 7.63 (d, *J* = 3.2 Hz, 1H), 7.54 (d, *J* = 8.7 Hz, 1H), 7.48 (dd, *J* = 12.4, 4.9 Hz, 3H), 7.43–7.28 (m, 3H), 7.13–7.04 (m, 1H), 7.04–6.98 (m, 2H), 6.63 (d, *J* = 3.0 Hz, 1H), 5.50 (s, 2H), 5.11 (dd, *J* = 12.9, 5.4 Hz, 1H), 4.78 (s, 2H), 4.60 (d, *J* = 5.5 Hz, 2H), 4.41 (t, *J* = 5.3 Hz, 2H), 3.73 (t, *J* = 5.3 Hz, 2H), 3.54–3.41 (m, 16H), 3.23–3.12 (m, 2H), 2.89 (m, 1H), 2.70 (t, *J* = 7.5 Hz, 2H), 2.64–2.53 (m, 2H), 2.06–2.02 (m, 1H), 2.02–1.93 (m, *J* = 15.2, 7.6 Hz, 2H). *1 × NH was not detected in this ^1^H NMR experiment. ^13^C NMR (75 MHz, DMSO-*d*_6_): 172.9, 170.0, 167.6, 167.1, 166.8, 165.6, 163.3, 161.2, 155.1, 145.4, 141.1, 141.0, 137.4, 137.1, 133.1, 130.8, 130.7, 129.8, 127.8, 125.5, 123.1, 123.0, 122.9, 122.6, 120.9, 120.7, 120.5, 116.8, 116.2, 114.4, 114.3, 113.9, 113.8, 109.9, 102.6, 69.9, 69.8, 69.7, 69.65, 69.6, 68.9, 68.8, 67.6, 50.5, 49.3, 48.9, 48.7, 38.5, 31.0, 23.3, 23.2, 22.1. ^19^F NMR (377 MHz, DMSO-*d*_6_): δ = −59.35, −113.06. LC-MS (ESI) *m*/*z*: calcd for C_54_H_58_F_4_N_9_O_13_S^+^ [M + H]^+^: 1148.38; found: 1148.55. HRMS (ESI) *m*/*z*: calcd for C_54_H_57_F_4_N_9_NaO_13_S^+^ [M + Na]^+^:1170.3626; found: 1170.3626. Purity (HPLC-UV 254 nm): 100%. *t*_R_ (method A) = 9.1 min.

#### *N*-(4-(Cyclopropanesulfonamido)-2-(trifluoromethyl)­benzyl)-1-((3-(1-(2-(2-((2-(2,6-dioxopiperidin-3-yl)-1,3-dioxoisoindolin-4-yl)­amino)-2-oxoethoxy)­ethyl)-1*H*-1,2,3-triazol-4-yl)­propyl)­sulfonyl)-1*H*-indole-5-carboxamide (**22a**)

**22a** was synthesized according to GP2 from **5** (15.0 mg, 0.026 mmol) and pomalidomide-PEG1-azide (**20a**) (12.3 mg, 0.029 mmol) as well as CuSO_4_·5H_2_O (1.9 mg, 0.0079 mmol) and sodium ascorbate (1.6 mg, 0.0079 mmol). The analytical pure product was obtained after preparative HPLC (method A) as a colorless solid in 56% yield (14.4 mg, 0.015 mmol). ^1^H NMR (500 MHz, DMSO-*d*_6_): δ = 11.16 (s, 1H), 10.22 (s, 1H), 10.07 (s, 1H), 9.10 (t, *J* = 5.8 Hz, 1H), 8.68 (d, *J* = 8.3 Hz, 1H), 8.27 (d, *J* = 1.2 Hz, 1H), 7.97–7.83 (m, 4H), 7.70–7.43 (m, 5H), 6.92 (d, *J* = 3.6 Hz, 1H), 5.16 (dd, *J* = 12.9, 5.3 Hz, 1H), 4.63 (d, *J* = 5.3 Hz, 2H), 4.60 (t, *J* = 5.2 Hz, 2H), 4.17 (s, 2H), 3.98 (t, *J* = 5.2 Hz, 2H), 3.75–3.65 (m, 2H), 2.96–2.84 (m, 1H), 2.74–2.55 (m, 5H), 2.12–2.02 (m, 2H), 1.87–1.76 (m, 2H), 0.99–0.92 (m, 4H). ^13^C NMR (101 MHz, DMSO-*d*_6_): δ = 172.8, 169.7, 168.7, 168.3, 166.7, 166.5, 145.0, 137.6, 136.6, 136.0, 135.9, 132.6, 131.3, 129.8, 129.8, 129.2, 128.2, 127.0, 126.7, 125.5, 124.6, 123.80, 123.5, 122.8, 122.7, 121.2, 118.5, 116.9, 116.8, 116.3, 112.6, 108.3, 69.6, 52.9, 49.0, 30.9, 29.7, 22.9, 21.9, 5.1, 1.2. ^19^F NMR (377 MHz, DMSO-*d*_6_): δ = −59.32. LC-MS (ESI) *m*/*z*: calcd for C_42_H_41_F_3_N_9_O_11_S_2_^+^ [M + H]^+^: 968.23; found: 968.40. HRMS (ESI) *m*/*z*: calcd for C_42_H_40_F_3_N_9_NaO_11_S_2_^+^ [M + Na]^+^: 990.2133; found: 990.2108. Purity (HPLC-UV 254 nm): 99%. *t*_R_ (method A) = 8.8 min.

#### *N*-(4-(Cyclopropanesulfonamido)-2-(trifluoromethyl)­benzyl)-1-((3-(1-(2-(2-(2-((2-(2,6-dioxopiperidin-3-yl)-1,3-dioxoisoindolin-4-yl)­amino)-2-oxoethoxy)­ethoxy)­ethyl)-1*H*-1,2,3-triazol-4-yl)­propyl)­sulfonyl)-1*H*-indole-5-carboxamide (**22b**)

**22b** was synthesized according to GP2 from **5** (15.0 mg, 0.026 mmol) and pomalidomide-PEG2-azide (**20b**) (12.9 mg, 0.029 mmol) as well as CuSO_4_·5H_2_O (1.9 mg, 0.0079 mmol) and sodium ascorbate (1.6 mg, 0.0079 mmol). The analytical pure product was obtained after preparative HPLC (method A) as a colorless solid in 51% yield (13.5 mg, 0.013 mmol). ^1^H NMR (500 MHz, DMSO-*d*_6_): δ = 11.14 (s, 1H), 10.32 (s, 1H), 9.11 (t, *J* = 5.7 Hz, 1H), 8.71 (d, *J* = 8.4 Hz, 1H), 8.29 (s, 1H), 7.96–7.82 (m, 3H), 7.73 (s, 1H), 7.69 (d, *J* = 3.6 Hz, 1H), 7.62 (d, *J* = 7.3 Hz, 1H), 7.57 (s, 1H), 7.54–7.46 (m, *J* = 8.9 Hz, 2H), 6.95 (d, *J* = 3.6 Hz, 1H), 5.15 (dd, *J* = 12.7, 5.4 Hz, 1H), 4.63 (d, *J* = 5.3 Hz, 2H), 4.43 (t, *J* = 5.1 Hz, 2H), 4.13 (s, 2H), 3.80 (t, *J* = 5.1 Hz, 2H), 3.70 (dd, *J* = 9.4, 5.8 Hz, 2H), 3.65–3.62 (m, 2H), 2.94–2.83 (m, 1H), 2.74–2.54 (m, 5H), 2.08 (dd, *J* = 16.2, 10.3 Hz, 1H), 1.87–1.72 (m, 2H), 1.03 (t, *J* = 7.2 Hz, 2H), 0.94 (d, *J* = 5.7 Hz, 4H). *1 × NH was not detected in this ^1^H NMR experiment. ^13^C NMR (75 MHz, DMSO-*d*_6_): δ = 172.7, 169.7, 169.2, 168.2, 166.7, 166.5, 144.9, 137.6, 136.5, 136.0, 135.9, 132.5, 131.3, 129.8, 129.8, 129.2, 128.3, 127.0, 126.7, 125.5, 124.4, 123.8, 123.5, 122.5, 121.2, 118.4, 116.1, 112.6, 108.3, 70.6, 70.1, 69.5, 68.8, 52.9, 49.3, 48.9, 45.6, 30.9, 29.7, 22.9, 21.9, 10.4, 5.1. ^19^F NMR (377 MHz, DMSO-*d*_6_): δ = −59.31. LC-MS (ESI) *m*/*z*: calcd for C_44_H_45_F_3_N_9_O_12_S_2_^+^ [M + H]^+^: 1012.25; found: 1012.45 HRMS (ESI): calcd for C_44_H_43_F_3_N_9_O_12_S_2_^–^ [M – H]^−^: 1010.243; found: 1010.2442. Purity (HPLC-UV 254 nm): 99%. *t*_R_ (method A) = 9.2 min.

#### *N*-(4-(Cyclopropanesulfonamido)-2-(trifluoromethyl)­benzyl)-1-((3-(1-(2-(2-(2-(2-((2-(2,6-dioxopiperidin-3-yl)-1,3-dioxoisoindolin-4-yl)­amino)-2-oxoethoxy)­ethoxy)­ethoxy)­ethyl)-1*H*-1,2,3-triazol-4-yl)­propyl)­sulfonyl)-1*H*-indole-5-carboxamide (**22c**)

**22c** was synthesized according to GP2 from **5** (15.0 mg, 0.026 mmol) and pomalidomide-PEG3-azide (**20c**) (14.9 mg, 0.029 mmol) as well as CuSO_4_·5H_2_O (1.9 mg, 0.0079 mmol) and sodium ascorbate (1.6 mg, 0.0079 mmol). The analytical pure product was obtained after preparative HPLC (method A) as a colorless solid in 57% yield (15.8 mg, 0.015 mmol). ^1^H NMR (400 MHz, DMSO-*d*_6_): δ = 10.33 (s, 1H), 9.12 (t, *J* = 5.7 Hz, 1H), 8.71 (dd, *J* = 8.5, 0.6 Hz, 1H), 8.29 (d, *J* = 0.9 Hz, 1H), 7.96–7.82 (m, 3H), 7.73 (s, 1H), 7.69 (d, *J* = 3.7 Hz, 1H), 7.64–7.60 (m, 1H), 7.57 (d, *J* = 1.9 Hz, 1H), 7.55–7.46 (m, 2H), 6.95 (d, *J* = 3.6 Hz, 1H), 5.15 (dd, *J* = 12.8, 5.4 Hz, 1H), 4.63 (d, *J* = 5.3 Hz, 2H), 4.39 (t, *J* = 5.3 Hz, 2H), 4.18 (s, 2H), 3.72 (dt, *J* = 11.7, 4.4 Hz, 6H), 3.61 (dd, *J* = 5.6, 3.5 Hz, 2H), 3.52–3.45 (m, 4H), 2.89 (ddd, *J* = 16.7, 13.7, 5.3 Hz, 1H), 2.72–2.53 (m, 5H), 2.12–2.01 (m, 1H), 1.87–1.76 (m, 2H), 0.98–0.92 (m, 4H). *2 × NH signals were not detected in this ^1^H NMR experiment. ^13^C NMR (75 MHz, DMSO-*d*_6_): δ = 172.8, 169.8, 169.4, 168.2, 166.7, 166.5, 144.8, 137.6, 136.5 136.0, 172.8, 169.8, 169.4, 168.2, 166.7, 166.5, 144.8, 137.6, 136.5 136.0, 135.9, 132.6, 131.3, 129.8, 129.7, 129.2, 128.3, 127.0, 126.7, 125.5, 124.4, 123.8, 123.5, 122.5, 121.2, 118.3, 116.9, 116.1, 112.6, 108.4, 70.8, 70.2, 69.8, 69.5, 68.7, 52.9, 49.2, 48.9, 30.9, 29.7, 22.9, 22.8, 21.9, 5.1, 1.2. ^19^F NMR (377 MHz, DMSO-*d*_6_): δ = −59.32. LC-MS (ESI) *m*/*z*: calcd for C_46_H_49_F_3_N_9_O_13_S_2_^+^ [M + H]^+^: 1056.28; found: 1056.45. HRMS (ESI) *m*/*z*: calcd for C_46_H_47_F_3_N_9_O_13_S_2_^–^ [M – H]^−^: 1054.2692; found: 1054.2699. Purity (HPLC-UV 254 nm): 99%. *t*_R_ (method A) = 8.8 min.

#### *N*-(4-(Cyclopropanesulfonamido)-2-(trifluoromethyl)­benzyl)-1-((3-(1-(14-((2-(2,6-dioxopiperidin-3-yl)-1,3-dioxoisoindolin-4-yl)­amino)-14-oxo-3,6,9,12-tetraoxatetradecyl)-1*H*-1,2,3-triazol-4-yl)­propyl)­sulfonyl)-1*H*-indole-5-carboxamide (**22d**)

**22d** was synthesized according to GP2 from **5** (11.0 mg, 0.019 mmol) and pomalidomide-PEG4-azide (**20d**) (11.1 mg, 0.021 mmol) as well as CuSO_4_·5H_2_O (1.5 mg, 0.0058 mmol) and sodium ascorbate (1.2 mg, 0.0058 mmol). The analytical pure product was obtained after preparative HPLC (method A) as a colorless solid in 71% yield (15.1 mg, 0.014 mmol). ^1^H NMR (400 MHz, DMSO-*d*_6_): δ = 11.05 (s, 1H), 10.34 (s, 1H), 9.11 (t, *J* = 5.7 Hz, 1H), 8.68 (d, *J* = 13.4 Hz, 1H), 8.28 (s, 1H), 7.95–7.82 (m, 3H), 7.73 (s, 1H), 7.69 (d, *J* = 3.7 Hz, 1H), 7.62 (d, *J* = 7.1 Hz, 1H), 7.57 (d, *J* = 1.8 Hz, 1H), 7.54–7.46 (m, 2H), 6.92 (t, *J* = 15.7 Hz, 1H), 5.15 (dd, *J* = 12.8, 5.4 Hz, 1H), 4.63 (d, *J* = 5.2 Hz, 2H), 4.40 (t, *J* = 5.2 Hz, 2H), 4.18 (s, 2H), 3.78–3.68 (m, 8H), 3.64 (dd, *J* = 5.7, 3.3 Hz, 3H), 3.53–3.47 (m, 5H), 2.96–2.81 (m, 1H), 2.72–2.54 (m, 5H), 2.15–1.98 (m, 1H), 1.89–1.72 (m, 2H), 0.96–0.93 (m, 4H). *1 × NH was not detected in this ^1^H NMR experiment. ^13^C NMR (101 MHz, DMSO-*d*_6_): δ = 172.8, 169.8, 169.4, 168.3, 166.7, 166.6, 144.9, 137.7, 136.6, 136.1, 135.9, 132.5, 131.3, 129.9, 129.8, 129.3, 128.3, 127.1, 126.8, 125.5, 124.4, 123.9, 123.5, 122.5, 121.2, 118.4, 117.0, 116.9, 116.1, 112.6, 108.4, 70.8, 70.3, 69.9, 69.7, 69.6, 69.5, 68.7, 52.9, 49.2, 49.0, 30.9, 29.7, 22.9, 21.9, 5.1, 1.2. ^19^F NMR (377 MHz, DMSO-*d*_6_): δ = −59.31. LC-MS (ESI) *m*/*z*: calcd for C_48_H_53_F_3_N_9_O_14_S_2_^+^ [M + H]^+^: 1100.31; found: 1100.50. HRMS (ESI) *m*/*z*: calcd for C_48_H_51_F_3_N_9_O_14_S_2_^–^ [M – H]^−^: 1098.2954; found: 1098.2944. Purity (HPLC-UV 254 nm): 99%. *t*_R_ (method A) = 9.1 min.

#### *N*-(4-(Cyclopropanesulfonamido)-2-(trifluoromethyl)­benzyl)-1-((3-(1-(17-((2-(2,6-dioxopiperidin-3-yl)-1,3-dioxoisoindolin-4-yl)­amino)-17-oxo-3,6,9,12,15-pentaoxaheptadecyl)-*1H*-1,2,3-triazole-4-yl)­propyl)­sulfonyl)-*1H*-indole-5-carboxamide (**22e**)

**22e** was synthesized according to GP2 from **5** (11.0 mg, 0.019 mmol) and pomalidomide-PEG5-azide (**20e**) (12.9 mg, 0.021 mmol) as well as CuSO_4_·5H_2_O (1.5 mg, 0.0058 mmol) and sodium ascorbate (1.2 mg, 0.0058 mmol). The analytical pure product was obtained after preparative HPLC (method A) as a colorless solid in 68% yield (15.0 mg, 0.013 mmol). ^1^H NMR (400 MHz, DMSO-*d*_6_): δ = 11.15 (s, 1H), 10.34 (s, 1H), 9.11 (t, *J* = 5.7 Hz, 1H), 8.70 (d, *J* = 8.4 Hz, 1H), 8.28 (s, 1H), 7.95–7.82 (m, 3H), 7.72 (s, 1H), 7.68 (d, *J* = 3.6 Hz, 1H), 7.61 (d, *J* = 7.3 Hz, 1H), 7.56 (s, 1H), 7.55–7.45 (m, 2H), 6.94 (t, *J* = 5.1 Hz, 1H), 5.13 (dd, *J* = 25.1, 12.7 Hz, 1H), 4.63 (d, *J* = 5.1 Hz, 2H), 4.40 (t, *J* = 5.1 Hz, 2H), 4.18 (s, 2H), 3.72 (dd, *J* = 12.6, 7.9 Hz, 8H), 3.67–3.62 (m, 3H), 3.54–3.45 (m, 9H), 2.95–2.83 (m, 1H), 2.74–2.53 (m, 5H), 2.12–2.02 (m, 1H), 1.87–1.75 (m, 2H), 0.99–0.89 (m, 4H). *1 × NH was not detected in this ^1^H NMR experiment. ^13^C NMR (101 MHz, DMSO-*d*_6_): δ = 172.9, 169.8, 169.5, 168.3, 166.8, 166.7, 144.9, 137.7, 136.6, 136.1, 136.0, 132.5, 131.4, 129.9, 129.8, 129.3, 128.3, 127.3, 126.7, 124.5, 123.9, 123.6, 122.6, 121.3, 118.4, 118.2, 117.1, 117.0, 116.1, 112.6, 108.5, 70.8, 70.3, 69.9, 69.8, 69.6, 69.5, 68.7, 52.9, 49.3, 49.0, 31.0, 29.8, 23.0, 22.9, 22.0, 5.1, 1.2. ^19^F NMR (377 MHz, DMSO-*d*_6_): δ = −59.32. LC-MS (ESI) *m*/*z*: calcd for C_50_H_57_F_3_N_9_O_15_S_2_^+^ [M + H]^+^: 1144.33; found: 1144.55. HRMS (ESI) *m*/*z*: calcd for C_50_H_55_F_3_N_9_O_15_S_2_^–^ [M – H]^−^: 1142.3217; found: 1142.3194. Purity (HPLC-UV 254 nm): 99%. *t*_R_ (method A) = 9.1 min.

#### *N*-(4-(Cyclopropanesulfonamido)-2-(trifluoromethyl)­benzyl)-1-((3-(1-(20-((2-(2,6-dioxopiperidin-3-yl)-1,3-dioxoisoindolin-4-yl)­amino)-20-oxo-3,6,9,12,15,18-hexaoxaicosyl)-1*H*-1,2,3-triazol-4-yl)­propyl)­sulfonyl)-1*H*-indole-5-carboxamide (**22f**)

**22f** was synthesized according to GP2 from **5** (11.0 mg, 0.019 mmol) and pomalidomide-PEG6-azide (**20f**) (13.9 mg, 0.021 mmol) as well as CuSO_4_·5H_2_O (1.5 mg, 0.0058 mmol) and sodium ascorbate (1.2 mg, 0.0058 mmol). The analytical pure product was obtained after preparative HPLC (method A) as a colorless solid in 67% yield (15.5 mg, 0.013 mmol). ^1^H NMR (400 MHz, DMSO-*d*_6_) δ = 11.12 (s, 1H), 10.35 (s, 1H), 10.07 (s, 1H), 9.11 (t, *J* = 5.7 Hz, 1H), 8.71 (d, *J* = 8.4 Hz, 1H), 8.28 (s, 1H), 7.96–7.81 (m, 3H), 7.73 (s, 1H), 7.69 (d, *J* = 3.7 Hz, 1H), 7.62 (d, *J* = 7.3 Hz, 1H), 7.57 (d, *J* = 1.8 Hz, 1H), 7.55–7.46 (m, 2H), 6.95 (d, *J* = 3.7 Hz, 1H), 5.15 (dd, *J* = 12.8, 5.4 Hz, 1H), 4.63 (d, *J* = 5.2 Hz, 2H), 4.40 (t, *J* = 5.2 Hz, 2H), 4.19 (s, 2H), 3.77–3.69 (m, 8H), 3.68–3.62 (m, 3H), 3.55–3.45 (m, 13H), 2.96–2.82 (m, 1H), 2.74–2.54 (m, 5H), 2.18–2.00 (m, 1H), 1.89–1.73 (m, 2H), 0.98–0.90 (m, 4H). ^13^C NMR (101 MHz, DMSO-*d*_6_): δ = 172.8, 169.8, 169.5, 168.3, 166.8, 166.6, 144.9, 137.6, 136.6, 136.1, 136.0, 132.6, 131.4, 129.9, 129.8, 129.3, 128.3, 127.1, 126.8, 125.5, 124.4, 123.9, 123.6, 122.6, 121.3, 118.4, 118.2, 116.1, 112.6, 108.4, 70.8, 70.3, 69.9, 69.8, 69.6, 69.5, 68.7, 52.1, 49.3, 49.0, 30.9, 29.8, 23.0, 22.9, 22.0, 5.1, 1.2. ^19^F NMR (377 MHz, DMSO-*d*_6_): δ = −59.33. LC-MS (ESI) *m*/*z*: calcd for C_52_H_61_F_3_N_9_O_16_S_2_^+^ [M + H]^+^: 1188.36; found: 1188.55. HRMS (ESI) *m*/*z*: calcd for C_52_H_59_F_3_N_9_O_16_S_2_^–^ [M – H]^−^: 1186.3479; found: 1186.3448. Purity (HPLC-UV 254 nm): 99%. *t*_R_ (method A) = 9.0 min.

#### 1-(3-Fluorobenzyl)-*N*-(4-(pent-4-yn-1-ylsulfonamido)-2-(trifluoromethyl)­benzyl)-1*H*-indole-5-carboxamide (**4**)

1-(3-fluorobenzyl)-*N*-(4-amino-2-(trifluoromethyl)­benzyl)-1*H*-indole-5-carboxamide (**11**) (100 mg, 0.25 mmol, 1.0 equiv) was dissolved in 10 mL of dry CHCl_3_, and the solution was sparged with argon. Then, 1-sulfonyl chloride-4-pentyne (52 mg, 0.29 mmol, 1.2 equiv) and pyridine (0.1 mL, 1.09 mmol, 5.0 equiv) were added. The reaction mixture was stirred at 55 °C under an argon atmosphere for 48 h. After cooling to r.t., the solution was acidified with 2 M aqueous HCl (pH = 3) and extracted with DCM (3 × 15 mL). The combined organic phase was dried over MgSO_4_, and the solvent was removed in vacuo. Purification using reversed-phase flash chromatography (ACN/H_2_O = 30:70 → 10:90) afforded **4** as a colorless solid in 74% yield (96 mg, 0.18 mmol). ^1^H NMR (400 MHz, DMSO-*d*_6_): δ (ppm) = 10.16 (s, 1H, N*H*), 8.92 (t,^3^ *J*_*HH*_ = 5.8 Hz, 1H, N*H*), 8.22 (d, *J* = 1.3 Hz, 1H), 7.70 (dd, *J* = 8.7 Hz, *J* = 1.6 Hz, 1H), 7.63 (d, *J* = 3.2 Hz, 1H), 7.54 (d, *J* = 8.9 Hz, 2H), 7.52–7.42 (m, 2H), 7.35 (m, 1H), 7.12–7.05 (m, 1H), 7.04–6.99 (m, 2H), 6.64 (dd, *J* = 3.2 Hz, *J* = 0.6 Hz, 1H*)*, 5.50 (s, 2H), 4.61 (d, *J* = 5.4 Hz, 2H), 3.23–3.17 (m, 2H), 2.76 (t, *J* = 2.6 Hz, 1H), 2.28 (td, *J* = 7.0 Hz, *J* = 2.6 Hz, 2H), 1.82 (m, 2H). ^13^C NMR (126 MHz, DMSO-*d*_6_) δ = 167.4, 158.0, 137.3, 137.2, 130.73, 130.7, 130.61, 130.57 127.7, 125.5, 122.9, 120.9, 120.6, 120.2, 114.3, 113.94, 113.88, 113.6, 109.8, 102.4, 82.9, 74.9, 72.22, 72.18, 72.1, 49.9, 48.6, 22.4, 16.2. ^19^F NMR (282 MHz, DMSO-*d*_6_): δ = −59,7 (s), −112,1 (s). LC-MS (ESI) *m*/*z*: calcd for C_29_H_26_F_4_N_3_O_3_S^+^: 572.16 [M + H]^+^; found: 572.23 [M + H]^+^, purity (254 nm): 99%. HRMS (MALDI) *m*/*z*: calcd for C_29_H_26_F_4_N_3_O_3_S^+^: 572.16255 [M + H]^+^; found: 572.16184 [M + H]^+^.

#### *N*-(4-(Cyclopropanesulfonamido)-2-(trifluoromethyl)­benzyl)-1-(pent-4-yn-1-ylsulfonyl)-1*H*-indole-5-carboxamide (**5**)

To a solution of *N*-(4-(cyclopropanesulfonamido)-2-(trifluoromethyl)­benzyl)-*1H*-indole-5-carboxamide (**16**) (124 mg, 0.28 mmol, 1.0 equiv) in dry DMF (5 mL) was added NaH (60% dispersion in mineral oil, 39 mg, 0.97 mmol, 3.7 equiv) and the mixture was sparged with argon. Then, pent-4-yne-1-sulfonyl chloride (84 mg, 0.48 mmol, 1.7 equiv) was added, and the reaction mixture was stirred at r.t. for 2 h under an argon atmosphere. The reaction was quenched with water (20 mL), and the solution was extracted with EtOAc (3 × 20 mL). The combined organic phase was washed with brine, dried over MgSO_4_, and concentrated in vacuo. The residue was purified using reversed phase flash chromatography (ACN/H_2_O = 30:70 → 90:10) to afford **5** as a colorless solid in 39% yield (62 mg, 0.11 mmol).^1^H NMR (300 MHz, DMSO-*d*_6_): δ = 10.06 (s, 1H), 9.11 (t, *J* = 5.8 Hz, 1H), 8.30 (s, 1H), 8.01–7.86 (m, 2H), 7.72 (d, *J* = 3.7 Hz, 1H), 7.57 (d, *J* = 1.9 Hz, 1H), 7.55–7.46 (m, 2H), 6.97 (d, *J* = 3.7 Hz, 1H), 4.63 (d, *J* = 5.3 Hz, 2H), 3.73–3.67 (m, 2H), 2.80 (t, *J* = 2.6 Hz, 1H), 2.68 (qd, *J* = 10.0, 5.2 Hz, 1H), 2.21 (td, *J* = 7.1, 2.6 Hz, 2H), 1.73–1.60 (m, 2H), 1.08–0.82 (m, 4H). ^13^C NMR (101 MHz, DMSO-*d*_6_): δ = 166.5, 137.6, 136.1, 132.5, 129.9, 129.8, 129.4, 128.3, 126.8, 123.9, 123.5, 121.3, 116.92, 116.86 112.6, 108.5, 82.4, 72.5, 52.4, 29.7, 29.0, 22.3, 16.0, 5.1. ^19^F NMR (282 MHz, DMSO-*d*_6_): δ = −59.31. LC-MS (ESI) *m*/*z*: calcd for C_25_H_25_F_3_N_3_O_5_S_2_^+^ [M + H]^+^: 568.11; found: 568.15.

#### *tert*-Butyl­(3-(*N*-(4-((1-(3-fluorobenzyl)-1*H*-indole-5-carboxamido)­methyl)-3-(trifluoromethyl)­phenyl)­sulfamoyl)­propyl)­carbamate (**26**)

1-(3-fluorobenzyl)-*N*-(4-amino-2-(trifluoromethyl)­benzyl)-1*H*-indole-5-carboxamide (**11**) (100 mg, 0.23 mmol, 1.0 equiv) was dissolved in 3 mL of dry CHCl_3_, and the solution was sparged with argon. Then, *tert*-butyl *N*-[3-(chlorosulfonyl)­propyl]­carbamate (74 mg, 0.27 mmol, 1.2 equiv) and pyridine (0.1 mL, 1.13 mmol, 5.0 equiv) were added. The reaction mixture was stirred at r.t. under an argon atmosphere for 72 h. Afterward, a saturated aqueous solution of NH_4_Cl was added. The aqueous phase was extracted with DCM (3 × 15 mL). The combined organic phase was dried over MgSO_4_, and the solvent was removed in vacuo. Purification using reversed-phase flash chromatography (ACN/H_2_O = 30:70 → 10:90) afforded **26** as a colorless solid in 48% yield (72 mg, 0.11 mmol). ^1^H NMR (400 MHz, DMSO-*d*_6_): δ = 10.12 (s, 1H), 8.92 (t, *J* = 5.8 Hz, 1H), 8.22 (d, *J* = 1.2 Hz, 1H), 7.71 (dd, *J* = 8.7, 1.6 Hz, 1H), 7.63 (d, *J* = 3.2 Hz, 1H), 7.58–7.48 (m, 3H), 7.45–7.40 (m, 1H), 7.39–7.31 (m, *J* = 8.0, 6.1 Hz, 1H), 7.08 (td, *J* = 8.3, 2.1 Hz, 1H), 7.04–6.98 (m, 2H), 6.88 (t, *J* = 5.6 Hz, 1H), 6.65–6.62 (m, 1H), 5.50 (s, 2H), 4.61 (d, *J* = 5.4 Hz, 2H), 3.10 (dd, *J* = 9.2, 6.6 Hz, 2H), 2.97 (q, *J* = 6.2 Hz, 2H), 1.83–1.71 (m, 2H), 1.30 (s, 9H). ^13^C NMR (151 MHz, DMSO-*d*_6_): δ = 167.3, 163.0, 161.4, 155.6, 140.9, 140.9, 137.3, 137.3, 132.9, 130.5, 129.7, 127.7, 125.5, 122.9, 120.9, 120.6, 114.2, 114.1, 113.8, 113.6, 109.7, 102.4, 77.6, 48.9, 48.6, 38.8, 38.1, 30.6, 28.1, 23.9. ^19^F NMR (282 MHz, DMSO-*d*_6_): δ = −59.33, −113.04. LC-MS (ESI) *m*/*z*: calcd for C_27_H_27_F_4_N_4_O_3_S^+^ [M + H-Boc]^+^: 563.17; found: 562.90.

#### *N*-(4-((3-Aminopropyl)­sulfonamido)-2-(trifluoromethyl)­benzyl)-1-(3-fluorobenzyl)-1*H*-indole-5-carboxamide (**27**)

To a solution of *tert*-butyl­(3-(*N*-(4-((1-(3-fluorobenzyl)-1*H*-indole-5-carboxamido)­methyl)-3-(trifluoromethyl)­phenyl)­sulfamoyl)­propyl)­carbamate (**26**) (40 mg, 0.06 mmol, 1.0 equiv) in DCM (1 mL) was added TFA (0.2 mL, 3.02 mmol, 50.0 equiv) and the reaction mixture was stirred at r.t. for 16 h. Afterward, a saturated aqueous solution of NaHCO_3_ was added (pH = 8), and the aqueous phase was extracted with DCM (3 × 15 mL). The combined organic phase was dried over MgSO_4_ and concentrated in vacuo to afford **27** as a yellow solid in 97% yield (33 mg, 0.06 mmol). The product was directly used in the synthesis of **24** without further purification. ^1^H NMR (400 MHz, DMSO-*d*_6_): δ = 8.85 (t, *J* = 5.5 Hz, 1H), 8.22 (s, 1H), 7.71 (d, *J* = 8.7 Hz, 1H), 7.62 (d, *J* = 3.2 Hz, 1H), 7.54 (d, *J* = 8.7 Hz, 1H), 7.43–7.31 (m, *J* = 13.4, 9.1 Hz, 3H), 7.24 (d, *J* = 8.9 Hz, 1H), 7.17–7.05 (m, 1H), 7.04–6.98 (m, *J* = 7.5 Hz, 2H), 6.63 (d, *J* = 3.1 Hz, 1H), 5.49 (s, 2H), 4.57 (d, *J* = 4.5 Hz, 2H), 3.03 (t, *J* = 7.3 Hz, 2H), 2.74 (s, 2H), 1.90–1.78 (m, 2H). * the NH and NH_2_ groups were not detectable in this 1H NMR experiment. ^19^F NMR (282 MHz, DMSO-*d*_6_): δ = −59.01, −113.03. LC-MS (ESI) *m*/*z*: calcd for C_27_H_27_F_4_N_4_O_3_S^+^ [M + H]^+^: 563.17; found: 563.05.

#### *tert*-Butyl-1-((2-(2,6-dioxopiperidin-3-yl)-1,3-dioxoisoindolin-4-yl)­oxy)-2-oxo-6,9,12,15-tetraoxa-3-azaoctadecan-18-oate (**29**)

To a solution of 2-((2-(2,6-dioxopiperidin-3-yl)-1,3-dioxoisoindolin-4-yl)­oxy)­acetic acid (**28**) (50 mg, 0.15 mmol, 1.0 equiv) and PyBOP (94 mg, 0.18 mmol, 1.2 equiv) in DMF (1.5 mL) was added Et_3_N (63 μL, 0.45 mmol, 3.0 equiv) and the mixture was stirred for 10 min at r.t. Subsequently, *tert-*butyl 1-amino-3,6,9,12-tetraoxapentadecan-15-oate (48 mg, 0.15 mmol, 1.0 equiv) was added to the reaction and the mixture was stirred for 16 h at r.t. The solution was diluted with EtOAc (15 mL) and washed with water (15 mL) and brine (15 mL). The aqueous phase was extracted with EtOAc (3 × 15 mL). The combined organic phase was dried over MgSO_4_, and the solvent was removed under reduced pressure. The resulting crude product was purified using reversed-phase flash chromatography (ACN/H_2_O = 30:70 → 90:10) to obtain **29** as a yellow oil in 52% yield (50 mg, 0.08 mmol). ^1^H NMR (400 MHz, DMSO-*d*_6_): δ = 11.11 (s, 1H), 8.00 (t, *J* = 5.6 Hz, 1H), 7.81 (dd, *J* = 8.4, 7.4 Hz, 1H), 7.50 (d, *J* = 7.3 Hz, 1H), 7.40 (d, *J* = 8.5 Hz, 1H), 5.11 (dd, *J* = 12.9, 5.4 Hz, 1H), 4.78 (s, 2H), 3.57 (t, *J* = 6.2 Hz, 2H), 3.53–3.43 (m, 15H), 3.31 (dd, *J* = 11.3, 5.6 Hz, 2H), 2.95–2.82 (m, 1H), 2.65–2.53 (m, 1H), 2.40 (t, *J* = 6.2 Hz, 2H), 2.09–1.97 (m, 1H),1.38 (s, 9H). LC-MS (ESI) *m*/*z*: calcd for C_30_H_40_N_3_O_12_^–^ [M – H]^−^: 634.26; found: 634.05.

#### 1-((2-(2,6-Dioxopiperidin-3-yl)-1,3-dioxoisoindolin-4-yl)­oxy)-2-oxo-6,9,12,15-tetraoxa-3-azaoctadecan-18-oic Acid (**30**)

To a solution of *tert*-butyl1-((2-(2,6-dioxopiperidin-3-yl)-1,3-dioxoisoindolin-4-yl)­oxy)-2-oxo-6,9,12,15-tetraoxa-3-azaoctadecan-18-oate (**29)** (113 mg, 0.18 mmol, 1.0 equiv) in DCM (1 mL) was added TFA (0.7 mL, 8.89 mmol, 50.0 equiv) and the reaction mixture was stirred at r.t. for 3 h. Afterward, the mixture was diluted with DCM (15 mL) and washed with water (3 × 15 mL). The combined aqueous phase was then extracted with DCM (3 × 15 mL). The combined organic phase was dried over MgSO_4_ and concentrated in vacuo to afford **30** as a yellow solid in 97% yield (100 mg, 0.17 mmol). ^1^H NMR (400 MHz, CDCl_3_) δ 9.41 (s, 1H), 7.77–7.71 (m, 2H), 7.54 (d, *J* = 7.1 Hz, 1H), 7.20 (d, *J* = 8.3 Hz, 1H), 5.04–4.94 (m, 1H), 4.67 (s, 2H), 3.75 (t, *J* = 6.0 Hz, 2H), 3.68–3.61 (m, 14H), 3.61–3.52 (m, *J* = 19.7, 9.2, 5.1 Hz, 3H), 2.93–2.74 (m, 3H), 2.58 (t, *J* = 5.9 Hz, 2H), 2.20–2.08 (m, 1H). ^13^C NMR (101 MHz, CDCl_3_) δ = 174.4, 171.9, 168.7, 167.2, 166.7, 165.9, 154.5, 137.0, 133.6, 119.4, 118.0, 117.3, 70.6, 70.5, 70.3, 70.2, 69.4, 67.9, 66.5, 49.3, 39.1, 34.9, 31.4, 22.6. LC-MS (ESI) *m*/*z*: calcd for C_26_H_34_N_3_O_12_^+^ [M + H]^+^: 580.21; found: 580.35.

#### *N*-(4-((1-((2-(2,6-Dioxopiperidin-3-yl)-1,3-dioxoisoindolin-4-yl)­oxy)-2,18-dioxo-6,9,12,15-tetraoxa-3,19-diazadocosane)­sulfonamido)-2-(trifluoromethyl)­benzyl)-1-(3-fluorobenzyl)-1*H*-indole-5-carboxamide (**24**)

To a solution of 1-((2-(2,6-dioxopiperidin-3-yl)-1,3-dioxoisoindolin-4-yl)­oxy)-2-oxo-6,9,12,15-tetraoxa-3-azaoctadecan-18-oic acid (**30**) (13.0 mg, 0.02 mmol, 1.0 equiv) and HATU (17 mg, 0.04 mmol, 2.0 equiv) in DMF (0.2 mL) was added Et_3_N (32 μL, 0.22 mmol, 10.0 equiv) and the mixture was stirred for 10 min at r.t. Subsequently, *N*-(4-((3-aminopropyl)­sulfonamido)-2-(trifluoromethyl)­benzyl)-1-(3-fluorobenzyl)-1*H*-indole-5-carboxamide (**27**) (12.6 mg, 0.02 mmol, 1.0 equiv) was added to the reaction and the mixture was stirred for 3 h at r.t. The solution was diluted with MeOH (3.8 mL) and directly subjected to preparative HPLC (method A) to afford **24** as a colorless solid in 24% yield (6.0 mg, 0.005 mmol). ^1^H NMR (400 MHz, DMSO-*d*_6_): δ = 11.11 (s, 1H), 8.91 (t, *J* = 5.8 Hz, 1H), 8.22 (d, *J* = 1.3 Hz, 1H), 8.00 (t, *J* = 5.6 Hz, 1H), 7.86 (t, *J* = 5.6 Hz, 1H), 7.80 (dd, *J* = 8.5, 7.4 Hz, 1H), 7.70 (dd, *J* = 8.7, 1.6 Hz, 1H), 7.63 (d, *J* = 3.2 Hz, 1H), 7.54 (d, *J* = 8.7 Hz, 1H), 7.50 (dd, *J* = 8.8, 4.7 Hz, 3H), 7.46–7.28 (m, 3H), 7.12–7.04 (m, 1H), 7.01 (d, *J* = 8.9 Hz, 2H), 6.63 (d, *J* = 3.2 Hz, 1H), 5.50 (s, 2H), 5.11 (dd, *J* = 12.9, 5.4 Hz, 1H), 4.78 (s, 2H), 4.60 (d, *J* = 5.3 Hz, 2H), 3.56–3.38 (m, 18H), 3.10 (dt, *J* = 12.7, 6.0 Hz, 4H), 2.95–2.83 (m, 1H), 2.64–2.52 (m, 2H), 2.22 (t, *J* = 6.4 Hz, 2H), 2.10–1.99 (m, 1H), 1.85–1.70 (m, 2H). *1 × N–H was not detected.^13^C NMR (101 MHz, DMSO-*d*_6_): δ = 172.8, 170.2, 169.9, 167.4, 166.9, 166.7, 165.4, 163.4, 161.0, 154.9, 141.0, 140.9, 137.7, 137.2, 136.9, 133.0, 132.9, 130.6, 130.5, 129.5, 127.7, 127.2, 126.8, 125.5, 123.0, 122.9, 122.8, 120.9, 120.6, 120.3, 116.8, 116.0, 114.3, 114.1, 113.9, 113.7, 109.8, 102.4, 69.7, 69.6, 69.4, 68.8, 67.5, 66.7, 48.9, 48.8, 48.6, 38.4, 36.70, 36.1, 30.9, 23.6, 21.9. ^19^F NMR (377 MHz, DMSO-*d*_6_): δ = −59.35, −113.04. LC-MS (ESI) *m*/*z*: calcd for C_53_H_58_F_4_N_7_O_14_S^+^ [M + H]^+^: 1124.37; found: 1124.15. Purity (HPLC-UV 254 nm): 99%. (*t*R= 9.2 min). HRMS (ESI): calcd for C_53_H_57_F_4_N_7_NaO_14_S^+^ [M + Na]^+^: 1146.3512; found: 1146.3511.

#### *N*-(14-Azido-3,6,9,12-tetraoxatetradecyl)-2-((2-(1-methyl-2,6-dioxopiperidin-3-yl)-1,3-dioxoisoindolin-4-yl)­oxy)­acetamide

To a solution of 2-((2-(1-methyl-2,6-dioxopiperidin-3-yl)-1,3-dioxoisoindolin-4-yl)­oxy)­acetic acid (68 mg, 0.20 mmol, 1.0 equiv) in dry DCM (1.5 mL) were added DIPEA (104 μL, 0.59 mmol, 3 equiv) and HATU (82 mg, 0.22 mmol, 1.1 equiv). After stirring the solution for 10 min, *O*-(2-Aminoethyl)-O’-(2-azidoethyl)­triethylene glycol (52 μL, 0.22 mmol, 1.1 equiv) was added and the reaction was stirred for 16 h at r.t. Afterward, the reaction mixture was diluted with EtOAc (10 mL) and washed with brine (10 mL). The aqueous phase was extracted with EtOAc (3 × 15 mL), and the combined organic phase was dried over MgSO_4_. The solvent was removed under reduced pressure, and the crude product was purified by reversed-phase flash chromatography (ACN/H_2_O = 30:70 → 90:10) to obtain the desired product as a yellow oil in 26% yield (30 mg, 0.05 mmol). ^1^H NMR (400 MHz, DMSO-*d*_6_): δ = 8.00 (t, *J* = 5.5 Hz, 1H), 7.84–7.80 (m, 1H), 7.50 (d, *J* = 7.3 Hz, 1H), 7.41 (d, *J* = 8.5 Hz, 1H), 5.18 (dd, *J* = 13.0, 5.4 Hz, 1H), 4.78 (s, 2H), 3.60–3.57 (m, 2H), 3.56–3.49 (m, 12H), 3.46 (t, *J* = 5.7 Hz, 2H), 3.40–3.36 (m, 2H), 3.32–3.28 (m, 2H), 3.02 (s, 3H), 3.01–2.88 (m, 1H), 2.81–2.73 (m, 1H), 2.56 (td, *J* = 13.4, 4.5 Hz, 1H), 2.11–2.01 (m, 1H). LC-MS (ESI) *m*/*z*: calcd for C_26_H_35_N_6_O_10_^+^ [M + H]^+^: 591.24; found: 591.40.

#### 1-(3-Fluorobenzyl)-*N*-(4-((3-(1-(1-((2-(1-methyl-2,6-dioxopiperidin-3-yl)-1,3-dioxoisoindolin-4-yl)­oxy)-2-oxo-6,9,12,15-tetraoxa-3-azaheptadecan-17-yl)-1*H*-1,2,3-triazol-4-yl)­propyl)­sulfonamido)-2-(trifluoromethyl)­benzyl)-1*H*-indole-5-carboxamide (**31**)

**31** was synthesized according to GP2 from **4** (10.6 mg, 18.6 mmol) and *N*-(14-azido-3,6,9,12-tetraoxatetradecyl)-2-((2-(1-methyl-2,6-dioxopiperidin-3-yl)-1,3-dioxoisoindo-lin-4-yl)­oxy)­acetamide (11.0 mg, 18.6 mmol). The analytical pure product was obtained after preparative HPLC (method A) as a colorless solid in 49% yield (10.6 mg, 0.009 mmol). ^1^H NMR (300 MHz, DMSO-*d*_6_): δ = 10.12 (s, 1H), 8.91 (t, *J* = 5.7 Hz, 1H), 8.23 (d, *J* = 1.3 Hz, 1H), 7.99 (t, *J* = 5.2 Hz, 1H), 7.84–7.76 (m, 2H), 7.71 (dd, *J* = 8.7, 1.6 Hz, 1H), 7.63 (d, *J* = 3.2 Hz, 1H), 7.57–7.46 (m, 4H), 7.44–7.29 (m, 3H), 7.13–6.97 (m, 3H), 6.64 (d, *J* = 2.7 Hz, 1H), 5.50 (s, 2H), 5.18 (dd, *J* = 13.0, 5.5 Hz, 1H), 4.78 (s, 2H), 4.61 (d, *J* = 5.0 Hz, 2H), 4.42 (t, *J* = 5.3 Hz, 2H), 3.73 (t, *J* = 5.2 Hz, 2H), 3.51–3.41 (m, *J* = 11.2, 5.0 Hz, 13H), 3.32–3.25 (m, 3H), 3.23–3.14 (m, 2H), 3.02 (s, 3H), 2.98–2.88 (m, 1H), 2.82–2.67 (m, 3H), 2.63–2.52 (m, 1H), 2.13–1.89 (m, 3H). ^13^C NMR (101 MHz, DMSO-*d*_6_): δ = 171.8, 169.6, 167.4, 166.9, 166.7, 165.4, 163.4, 161.0, 155.0, 145.3, 141.0, 140.9, 137.3, 136.9, 133.0, 130.7, 130.6, 129.6, 127.7, 125.5, 123.0, 122.9, 122.4, 120.9, 120.6, 120.4, 116.7, 116.1, 114.3, 114.1, 113.9, 113.7, 109.8, 102.5, 69.8, 69.7, 69.6, 69.5, 68.8, 68.7, 67.5, 50.4, 49.4, 49.2, 48.6, 31.1, 26.6, 23.2, 23.1, 21.2. ^19^F NMR (377 MHz, DMSO-*d*_6_): δ = −59.39, −113.03. LC-MS (ESI) *m*/*z*: calcd for C_55_H_60_F_4_N_9_O_13_S^+^ [M + H]^+^: 1162.39.; found: 1162.65. HRMS (ESI) *m*/*z*: calcd for C_55_H_58_F_4_N_9_O_13_S^–^ [M – H]^−^: 1160.3816; found: 1160.3792. Purity (HPLC-UV 254 nm): 99%. *t*_R_ (method A) = 9.9 min.

#### *tert*-Butyl 1-((2-(1-Methyl-2,6-dioxopiperidin-3-yl)-1,3-dioxoisoindolin-4-yl)­oxy)-2-oxo-6,9,12,15-tetraoxa-3-azaoctadecan-18-oate

To a solution of 2-((2-(1-methyl-2,6-dioxopiperidin-3-yl)-1,3-dioxoisoindolin-4-yl)­oxy)­acetic acid (100 mg, 0.29 mmol, 1.0 equiv) in dry DMF (1.0 mL) were added DIPEA (152 μL, 0.87 mmol, 3.0 equiv) and HATU (121 mg, 0.32 mmol, 1.1 equiv). After stirring the solution for 10 min, *tert*-butyl 1-amino-3,6,9,12-tetraoxapentadecan-15-oate (107 μL, 0.35 mmol, 1.2 equiv) was added, and the reaction was stirred for 16 h at r.t. Afterward, the reaction mixture was diluted with EtOAc (10 mL) and washed with brine (10 mL). The aqueous phase was extracted with EtOAc (3 × 15 mL), and the combined organic phase was dried over MgSO_4_. The solvent was removed under reduced pressure, and the crude product was purified by reversed-phase flash chromatography (ACN/H_2_O = 30:70 → 90:10) to obtain the product as a yellow oil in 10% yield (18 mg, 0.03 mmol). ^1^H NMR (400 MHz, DMSO-*d*_6_): δ = 8.00 (t, *J* = 5.5 Hz, 1H), 7.85–7.79 (m, 1H), 7.50 (d, *J* = 7.3 Hz, 1H), 7.41 (d, *J* = 8.5 Hz, 1H), 5.18 (dd, *J* = 13.0, 5.4 Hz, 1H), 4.78 (s, 2H), 3.57 (t, *J* = 6.2 Hz, 2H), 3.53–3.43 (m, 15H), 3.30 (d, *J* = 5.4 Hz, 1H), 3.02 (s, 3H), 2.98–2.89 (m, 1H), 2.82–2.72 (m, 1H), 2.61–2.52 (m, 1H), 2.40 (t, *J* = 6.2 Hz, 2H), 2.12–1.95 (m, 1H), 1.38 (s, 9H). LC-MS (ESI) *m*/*z*: calcd for C_31_H_44_N_3_O_12_^+^ [M + H]^+^: 650.29; found: 650.15.

#### 1-((2-(1-Methyl-2,6-dioxopiperidin-3-yl)-1,3-dioxoisoindolin-4-yl)­oxy)-2-oxo-6,9,12,15-tetraoxa-3-azaoctadecan-18-oic Acid

To a solution of *tert-*butyl 1-((2-(1-methyl-2,6-dioxopiperidin-3-yl)-1,3-dioxoisoindolin-4-yl)­oxy)-2-oxo-6,9,12,15-tetraoxa-3-azaoctadecan-18-oate (22 mg, 0.03 mmol, 1.0 equiv) in DCM (0.2 mL) was added TFA (0.2 mL, 1.69 mmol, 50.0 equiv) and the reaction mixture was stirred at r.t. for 3 h. Afterward, the mixture was diluted with DCM (15 mL) and washed with water (3 × 15 mL). The combined aqueous phase was then extracted with DCM (3 × 15 mL). The combined organic phase was dried over MgSO_4_ and concentrated in vacuo to afford the product as a brown oil in 77% yield (15 mg, 0.03 mmol), which was used in the next step without further purification. LC-MS (ESI) *m*/*z*: calcd for C_27_H_36_N_3_O_12_^+^ [M + H]^+^: 594.23; found: 594.25.

#### 1-(3-Fluorobenzyl)-*N*-(4-((1-((2-(1-methyl-2,6-dioxopiperidin-3-yl)-1,3-dioxoisoindolin-4-yl)­oxy)-2,18-dioxo-6,9,12,15-tetraoxa-3,19-diazadocosane)­sulfon-amido)-2-(trifluoromethyl)­benzyl)-1*H*-indole-5-carboxamide (**32**)

To a solution of 1-((2-(1-methyl-2,6-dioxopiperidin-3-yl)-1,3-dioxoisoindolin-4-yl)­oxy)-2-oxo-6,9,12,15-tetraoxa-3-azaoctadecan-18-oic acid (14.0 mg, 0.024 mmol, 1.0 equiv) and HATU (17.9 mg, 0.048 mmol, 2.0 equiv) in DMF (0.7 mL) was added Et_3_N (33 μL, 0.24 mmol, 10.0 equiv) and the mixture was stirred for 10 min at r.t. Subsequently, *N*-(4-((3-aminopropyl)­sulfonamido)-2-(trifluoromethyl)­benzyl)-1-(3-fluorobenzyl)-*1H*-indole-5-carbox-amide (**27**) (14.6 mg, 0.03 mmol, 1.1 equiv) was added to the reaction and the mixture was stirred for 3 h at r.t. The solution was diluted with MeOH (3.8 mL) and directly subjected to preparative HPLC (method A) to afford **32** as a colorless solid in 24% yield (6.0 mg, 0.005 mmol).^1^H NMR (500 MHz, DMSO-*d*_6_): δ = 10.15 (s, 1H), 8.90 (t, *J* = 5.8 Hz, 1H), 8.22 (d, *J* = 1.2 Hz, 1H), 8.00 (t, *J* = 5.6 Hz, 1H), 7.85 (t, *J* = 5.7 Hz, 1H), 7.80 (dt, *J* = 11.9, 6.0 Hz, 1H), 7.70 (dd, *J* = 8.7, 1.6 Hz, 1H), 7.62 (d, *J* = 3.2 Hz, 1H), 7.54 (d, *J* = 8.7 Hz, 1H), 7.52–7.46 (m, 3H), 7.44–7.31 (m, 3H), 7.12–7.05 (m, 1H), 7.01 (dd, *J* = 6.4, 5.5 Hz, 2H), 6.65–6.61 (m, 1H), 5.50 (s, 2H), 5.18 (dd, *J* = 13.0, 5.4 Hz, 1H), 4.78 (s, 2H), 4.60 (d, *J* = 5.4 Hz, 2H), 3.55–3.39 (m, 15H), 3.31–3.23 (m, *J* = 5.6 Hz, 3H), 3.13–3.06 (m, 4H), 3.02 (s, 3H), 2.95 (ddd, *J* = 17.3, 14.0, 5.4 Hz, 1H), 2.80–2.72 (m, 1H), 2.59–2.52 (m, 1H), 2.22 (t, *J* = 6.4 Hz, 2H), 2.05 (ddt, *J* = 10.8, 5.2, 2.9 Hz, 1H), 1.81–1.73 (m, 2H). ^13^C NMR (126 MHz, DMSO-*d*_6_): δ = 171.8, 170.1, 169.6, 167.4, 166.9, 166.7, 165.4, 163.2, 161.2, 155.0, 140.9, 137.3, 136.9, 133.0, 130.6, 130.5, 129.7, 127.7, 125.5, 122.9, 122.8, 120.9, 120.6, 120.4, 116.7, 116.1, 114.3, 114.1, 113.8, 113.7, 109.8, 102.4, 69.8, 69.7, 69.6, 69.5, 69.4, 69.3, 67.55, 66.7, 49.3, 48.9, 48.6, 38.4, 36.7, 36.1, 31.1, 26.6, 23.7, 21.2. ^19^F NMR (471 MHz, DMSO-*d*_6_): δ = −59.33, −113.04. LC-MS (ESI) *m*/*z*: calcd for C_54_H_60_F_4_N_7_O_14_S^+^ [M + H]^+^: 1138.38.; found: 1138.25. HRMS (ESI) *m*/*z*: calcd for C_54_H_58_F_4_N_7_O_14_S^+^ [M – H]^−^: 1136.3704; found: 1136.3682. Purity (HPLC-UV 254 nm): 99%. *t*_R_ (method A)= 9.7 min.

### sEH-H Activity Assay

The in vitro potency of the synthesized PROTACs toward sEH-H was determined using a fluorescence-based activity assay with the fluorogenic substrate (3-phenyl-cyano­(6-methoxy-2-naphthalenyl)­methyl ester-2-oxiraneacetic acid) PHOME^29^ according to a published protocol.^34^ For the assay, human and murine full-length sEH were used, which were expressed and purified as described by Lukin et al.^35^ and Lillich et al.,^24^ respectively.

In brief, dilution series of each tested compound were incubated with either human full-length sEH (final concentration 3 nM) or with murine full-length sEH (final concentration 10 nM) in a 96-well plate (black, flat bottom) for 30 min. As a positive control, the protein was incubated with DMSO vehicle. After incubation, an aqueous solution of PHOME (final concentration 50 μM) was added quickly to each well. The fluorescence was then measured every 60 s for 45 min using a *Tecan* Infinite F200 pro multimode plate reader (excitation: 360 nm, emission: 465 nm; bandwidth 35 nm). Inhibition [%] was plotted against the logarithmic concentration, and IC_50_-values were determined using the nonlinear regression curve fit “log­(Inhibitor) vs. Response–Variable slope (four parameters)” in Prism 7.0.

### sEH HiBiT Assay

HeLa^sEH‑HiBiT^ cells were maintained at 37 °C and 5% CO_2_ and split twice a week (1.8 mio cells per flask). The cells were cultivated in 175 cm^2^ cell culture flasks *(greiner BIO-ONE*) in DMEMsup (3 mL DMEM (1X) medium with phenol red (Thermo Fisher Scientific, #41965–039) supplemented with 10% Corning Fetal Bovine Serum (Corning, 35–079-CV), penicillin (100 units/ml), and streptomycin (100 μg/mL) (Gibco #15140), and 1 mM sodium pyruvate (Gibco #11360)). For splitting, Gibco Trypsin-EDTA and Gibco DPBS (no calcium, no magnesium), purchased from Thermo Fisher Scientific, were used. In preparation for the assay, 8 mio cells were seeded into a 175 cm^2^ cell culture flask and incubated for 24 h at 37 °C and 5% CO_2_. Afterward, cells were harvested in DMEMsup, cell density was adjusted to 4 x10^5^ cells/mL, and the cells were seeded into a 384-well TC plate (Nunc white polystyrole, flat bottom, Cat. Nr. 164610) using a Multidrop combi (*Thermo Fisher Scientific*) at 50 μL/well resulting in 2000 cells per well. The plate was sealed with a semipermeable AeraSeal film (*Sigma-Aldrich/Merck*, A9224), and cells were incubated for 24 h at 37 °C and 5% CO_2_. Compound dilutions, including the positive control **2** with a final concentration of 300 nM in the assay, and dilution series of the tested compounds were prepared in DMEMsup (final DMSO concentration 5.5%) from respective compound stocks in DMSO or pure DMSO. Five μL of the respective dilutions were added to the cells in triplicate for a final volume of 55 μL and a final DMSO concentration of 0.5%. The plate was centrifuged for 1 min at 300 rpm, then resealed with AeraSeal film, and the cells were then incubated for 24 h at 37 °C and 5% CO_2_. The same procedure was conducted for incubation times of 18 h, 3 and 1 h. After incubation with the compound dilutions for the respective time intervals, cells were washed four times with DPBS using a Hydrospeed plate washer device (*Tecan*) with a remaining volume of 10 μL in each well. Cell lysis was performed by adding 1 μL of Mammalian Lysis Buffer (*Promega*) to each well, which was followed by centrifugation for 1 min at 300 rpm and incubation for 10 min at rt. Meanwhile, the Nano-Glo substrate mix was freshly prepared from 2600 μL Nano-Glo HiBiT Extracellular Buffer, 52 μL Nano-Glo HiBiT Extracellular Substrate, and 26 μL LgBiT Protein (all part of the Nano-Glo HiBiT Extracellular Detection System Kit, *Promega*). Subsequently, 10 μL of the Nano-Glo substrate mix was added to each well, followed by centrifugation for 1 min at 300 rpm. After incubation for 10 min at rt, the luminescence signal was detected using a Spark Multimode Microplate Reader (*Tecan*). The mean luminescence signal of each triplicate relative to the mean DMSO control signal was plotted against time or concentration in Prism 7.0 (*GraphPad Software, Inc.*). To determine the DC_50_ values, the normalized luminescence signals were plotted against the logarithmic compound concentration, and data analysis was performed using the nonlinear regression curve fit “log­(Inhibitor) vs. response–variable slope (four parameters)” in Prism 7.0.

## Supplementary Material









## Notes

Predicted pose of FL217 in hsEH-H (Figure S1); the HiBiT assay data of sEH PROTAC **2** (Figure S2); HiBiT assay results of **23** treatment of HeLa^sEH‑HiBiT^ cells after 48 h (Figure S3); the control experiments of PROTAC **24** with the HiBiT assay to show proteasomal degradation (Figure S4); water solubility measurements, cytotoxicity and metabolic stability in murine liver microsomes of PROTACs **23** and **24** (Figure S5); volcano plot of the proteomics analysis of PCLS (Figure S6); HPLC traces of PROTACs **23** and **24**; chemical–biological methods of cloning, crystallization, metabolic stability, and cytotoxicity testing; target engagement test methods; and methods and sample preparation for the data from mouse hepatocytes, human M1 macrophages, and human lung tissue (PDF)
Molecular formula strings (CSV)
sEH-H in complex with sEH_and_FL217_with_triazole_linker_indole (PDB)
sEH-H in complex with sEH_and_FL217_sEH_triazole_linker_sulfonamide (PDB)

## Abbreviations

CRBN: cereblon
CuAAC: copper-catalyzed azide–alkyne cycloaddition
EpETrEs: epoxyeicosatrienoic acids
DC_50_: half-maximal degradation concentration
DiHETrE: dihydroxyeicosatrienoic acids
*D*_max_: maximum fractional amount of degraded protein
HOBt: 1-hydroxybenzotriazol
hsEH-H: human soluble epoxide hydrolase domain
msEH-H: murine soluble epoxide hydrolase domain
POI: protein of interest, PROTAC, Proteolysis targeting chimeras
PyBOP: benzotriazol-1-yl-oxytripyrrolidinophosphonium-hexafluorophosphat
sEH: soluble epoxide hydrolase
sEH-H: soluble epoxide hydrolase domain
sEH-P: soluble epoxide phosphatase domain
TFA: trifluoroacetic acid
