# Supplementary material for: Structure-Based Design of PROTACS for the Degradation of Soluble Epoxide Hydrolase
Source: J Med Chem. 2025 Jun 18;68(13):13728–49. doi: 10.1021/acs.jmedchem.5c00552 (PMC12257526; doi:10.1021/acs.jmedchem.5c00552)
Supplement: Supplementary file 1 [file jm5c00552_si_001.pdf]

Supporting Information

Structure-based Design of PROTACS for the Degradation of Soluble Epoxide Hydrolase

Julia Schönfeld[1,‡], Steffen Brunst,[1,7,‡], Ludmila Ciomirtan[2], Lena Willmer[3,4], Michel A. Chromik[5], Adarsh Kumar[1], Timo Froemel[6], Nick Liebisch[1], Arne Hackspacher[1], Johanna H. M. Ehrler[1], Lukas Wintermeier[2], Christina Hesse[3,4], Jan Fiedler[3], Jan Heering[7], Hinrich Freitag[4,8], Patrick Zardo[9], Hans-Gerd Fieguth[10], Astrid Brüggerhoff[1], Josefine Jakob[11, 12], Björn Häupl[11,12,13,14], Lilia Weizel[1], Astrid Kaiser[1], Manfred Schubert-Zsilavecz[1], Thomas. Oellerich[11,12,13,14,15], Ingrid Fleming[6], Nils H. Schebb[5], Robert Fürst[16], Aimo Kannt[7,17], Stefan Knapp[1, 18], Ewgenij Proschak[1,7], Kerstin Hiesinger[1,]*

[1] Institute of Pharmaceutical Chemistry, Goethe University, 60438 Frankfurt am Main, Germany

[2] Institute of Pharmaceutical Biology, Goethe University, 60438 Frankfurt am Main, Germany

[3] Fraunhofer Institute for Toxicology and Experimental Medicine ITEM, Member of Fraunhofer Cluster Immune Mediated Diseases (CIMD), 60596 Frankfurt am Main, Germany

[4] Biomedical Research in Endstage and Obstructive Lung Disease Hannover (BREATH), Member of the German Center for Lung Research (DZL), 30625 Hannover, Germany

[5] Chair of Food Chemistry, Faculty of Mathematics and Natural sciences, University of Wuppertal, 42119 Wuppertal, Germany.

[6] Institute for Vascular Signalling, Centre for Molecular Medicine, Goethe University, 60596 Frankfurt am Main, Germany

[7] Fraunhofer Institute for Translational Medicine and Pharmacology ITMP, 60596 Frankfurt am Main, Germany

[8] Institute of Pathology, Hannover Medical School, 30625 Hannover, Germany

[9] Department of Cardiothoracic Transplantation and Vascular Surgery, Hannover Medical School, 30625 Hannover, Germany

[10] KRH Clinics Hannover, 30459 Hannover, Germany

[11] Department of Medicine, Hematology and Oncology, University Hospital, Goethe University Frankfurt, 60596 Frankfurt am Main, Germany

[12]Frankfurt Cancer Institute (FCI), 60596 Frankfurt am Main, Germany

[13] German Cancer Consortium (DKTK), partner site Frankfurt/Mainz, a partnership between DKFZ and UCT Frankfurt-Marburg, Germany, 60590 Frankfurt am Main, Germany

[14] German Cancer Research Center (DKFZ), 69120 Heidelberg, Germany

[15] University Cancer Center (UCT), 60590 Frankfurt am Main, Germany

[16] Pharmaceutical Biology, Department of Pharmacy – Center for Drug Research, Ludwig-Maximilians-Universität München, 81377 Munich, Germany

[17] Institute for Clinical Pharmacology, Goethe University, 60596 Frankfurt am Main, Germany

[18] Structural Genomics Consortium (SGC), Buchmann Institute for Life Sciences, 60438 Frankfurt/Main, Germany

Corresponding Author

*Hiesinger@pharmchem.uni-frankfurt.de

Content

Supplementary figures S4
HPLC traces S7
Chemical biology S9
Cloning of the sEH-HiBiT construct and generation of the cell line HeLa$^{sEH\text{-}HiBiT}$ S9
sEH HiBiT Assay S12
sEH-H activity assay S13
solubility limit assay S13
Evaluation of the linker length with MOE S13
Crystallography S14
CellTiter-Glo Assay S15
Metabolic stability in mouse and rat liver microsomes S16
Target engagement assay and proteomics S16
Experiments in primary mouse hepatocytes S17
Experiments in human M1 macrophages S18
Experiments in precision-cut lung slices S20
References S22

Supplementary figures

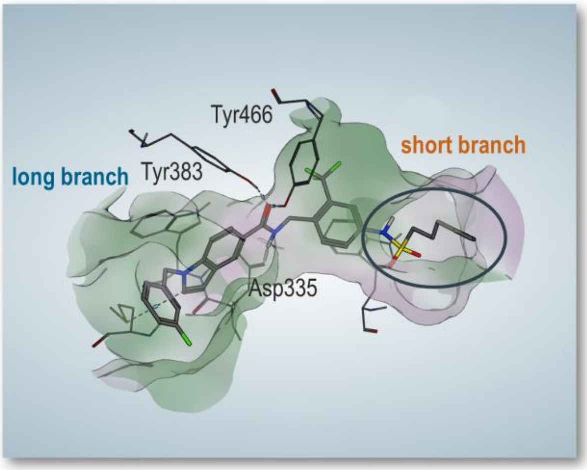

Figure S1: Predicted binding pose of prolonged inhibitor scaffold FL217 with the software MOE. The crystal structure of sEH-H in complex with inhibitor FL217 was used for this experiment (PDB code: 7P4K).

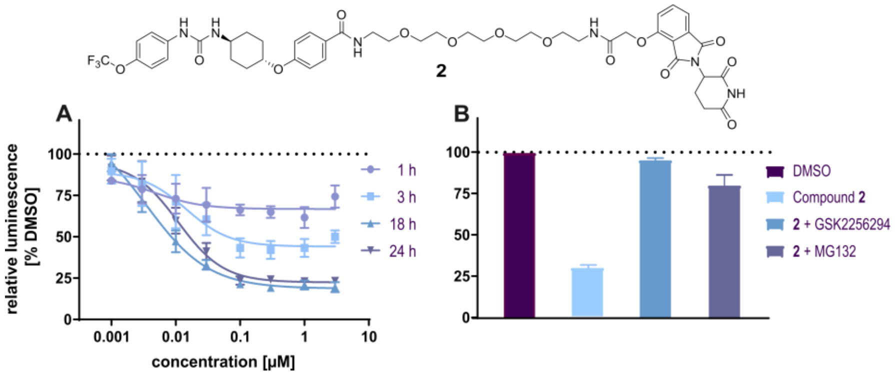

Figure S2: sEH PROTAC 2 (Wang et al.[1]) was used as a positive control in the sEH HiBiT assay. A): time and concentration dependent sEH degradation (incubation times: 1 h, 3 h, 18 h, 24 h). B) Control experiments for mode of action. HeLa$^{sEH\text{-}HiBiT}$ were co-treated with **2** [300 nM] and sEH-H inhibitor GSK2256294 [3 µM] or proteasome inhibitor MG 132 [3 µM] for 18 h.

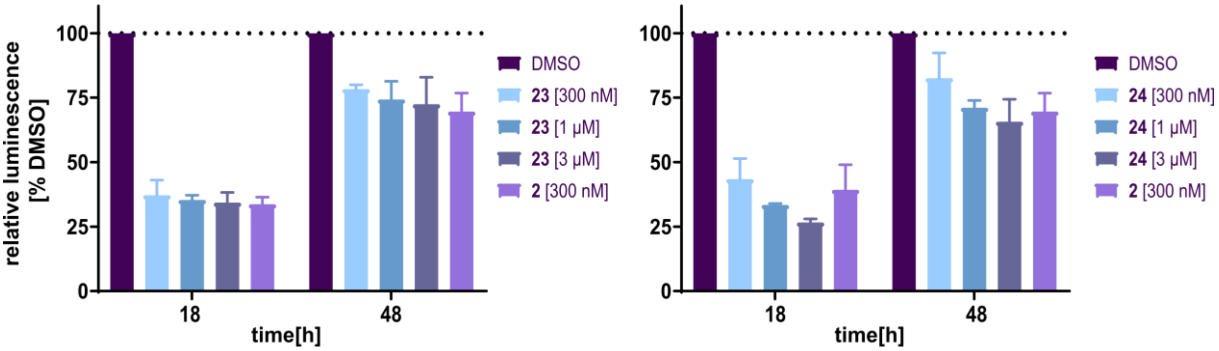

Figure S3: Less sEH degradation was observed after 48 h, due to re-synthesis of sEH or metabolic instability of the respective PROTAC.

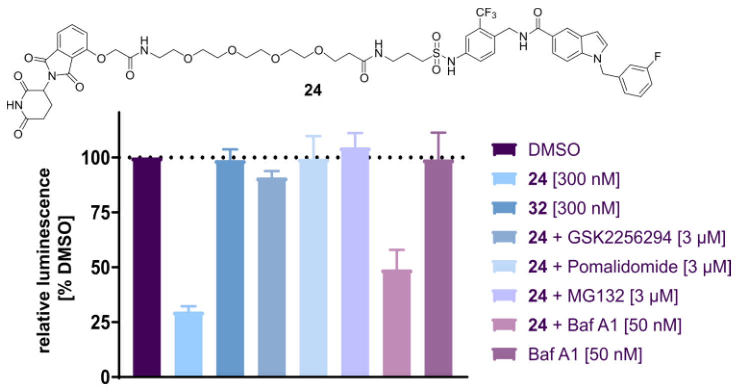

Figure S4: Control experiments for PROTAC **24** (N=3). HeLa$^{sEH\text{-}HiBiT}$ were co-treated with **24** [300 nM] and sEH-H inhibitor GSK2256294 [3 µM] or CRBN ligand Pomalidomide [3 µM] or proteasome inhibitor MG 132 [3 µM] or lysosome inhibitor Bafylomicin A1 [50 nM] for 18 h.

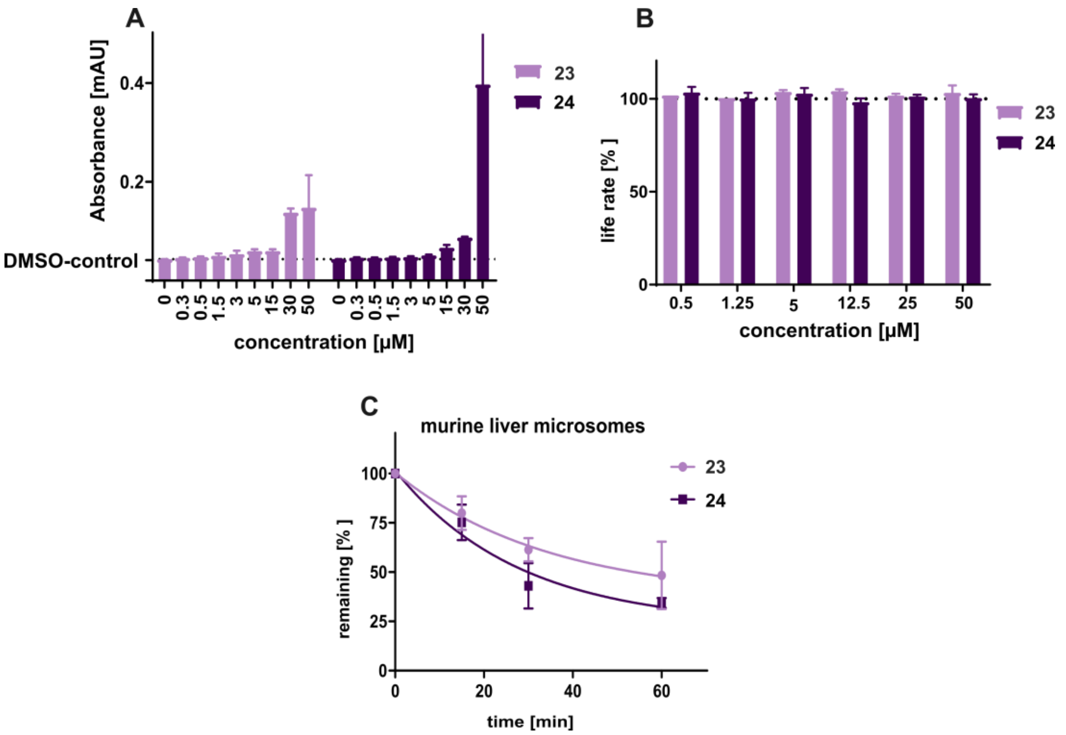

Figure S5: Biochemical characterization of PROTACs **23** and **24**. A) Water solubility was measured at concentrations from 0.3-50 µM (for experimental details see “solubility limit assay”). N=3. B) Cell viability was measured using the Cell Titer Glo assay kit (Promega). HepG2 cells were treated for 72 h with **23** or **24** at indicated concentrations. N=3. C) Metabolic stability was evaluated in murine liver microsomes (for experimental details see “metabolic stability in mouse and rat liver microsomes”). N=3.

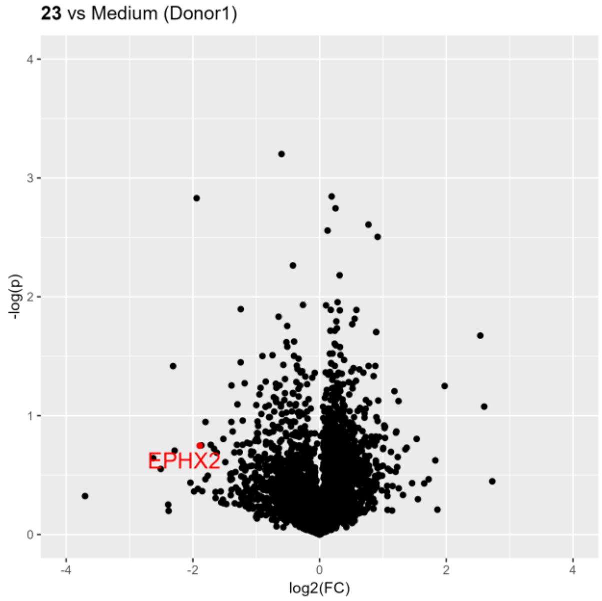

Figure S6: Volcano plot of proteomics analysis of the treated human lung tissue (precision-cut lung slices) with LC–MS/MS analysis.

HPLC traces

<Sample Information>

Sample Name : Compound **24**
Sample ID : JSF268
Data Filename : JSF268 pff_11.10.2023_002.lcd
Method Filename : C18_Scout_Gradient_70-10_20min.lcm
Batch Filename : Batch File 70-10 JSF geringe Massen.lcb

Vial #	: 1-61	Sample Type	: Unknown
Injection Volume	: 10 uL		
Date Acquired	: 11.10.2023 19:20:43	Acquired by	: System Administrator
Date Processed	: 11.10.2023 19:40:45	Processed by	: System Administrator

<Chromatogram>

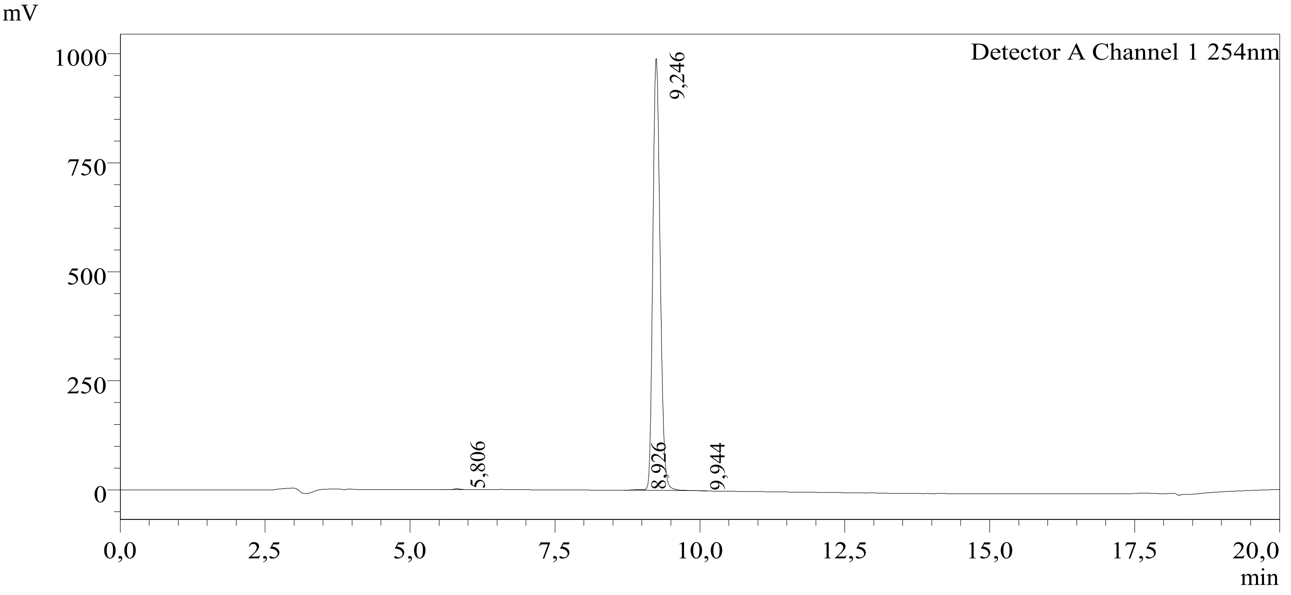

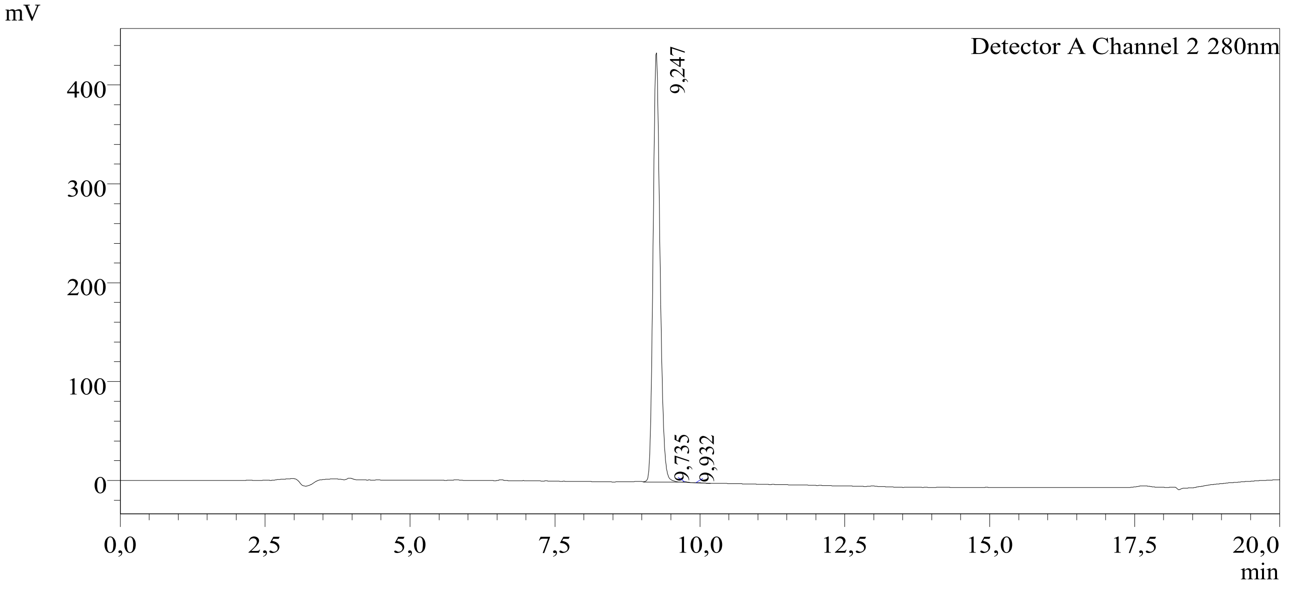

Peak Table

Detector A Channel 1 254nm

Peak#	Ret. Time	Area	Height	Conc.	Unit	Mark	Name
1	5,806	11019	1979	0,125			
2	8,926	19354	1857	0,220			
3	9,246	8771717	990015	99,597		V	
4	9,944	5151	687	0,058			
Total		8807242	994538				

Detector A Channel 2 280nm

Peak#	Ret. Time	Area	Height	Conc.	Unit	Mark	Name
1	9,247	3619837	434012	99,820			
2	9,735	4245	630	0,117			
3	9,932	2286	286	0,063			
Total		3626367	434928				

<Sample Information>

Sample Name : Compound **23**
Sample ID :JSF252
Data Filename : JSF252 pf_06.09.2023_004.lcd
Method Filename : C18_Scout_Gradient_70-10_20min_550-1500mz.lcm
Batch Filename : Batch File 70-10 JSF 550-1500 mz.lcb
Vial # : 1-101 Sample Type : Unknown
Injection Volume : 10 uL
Date Acquired : 06.09.2023 19:47:09 Acquired by : System Administrator Date Processed : 08.09.2023 09:30:29 Processed by : System Administrator

<Chromatogram>

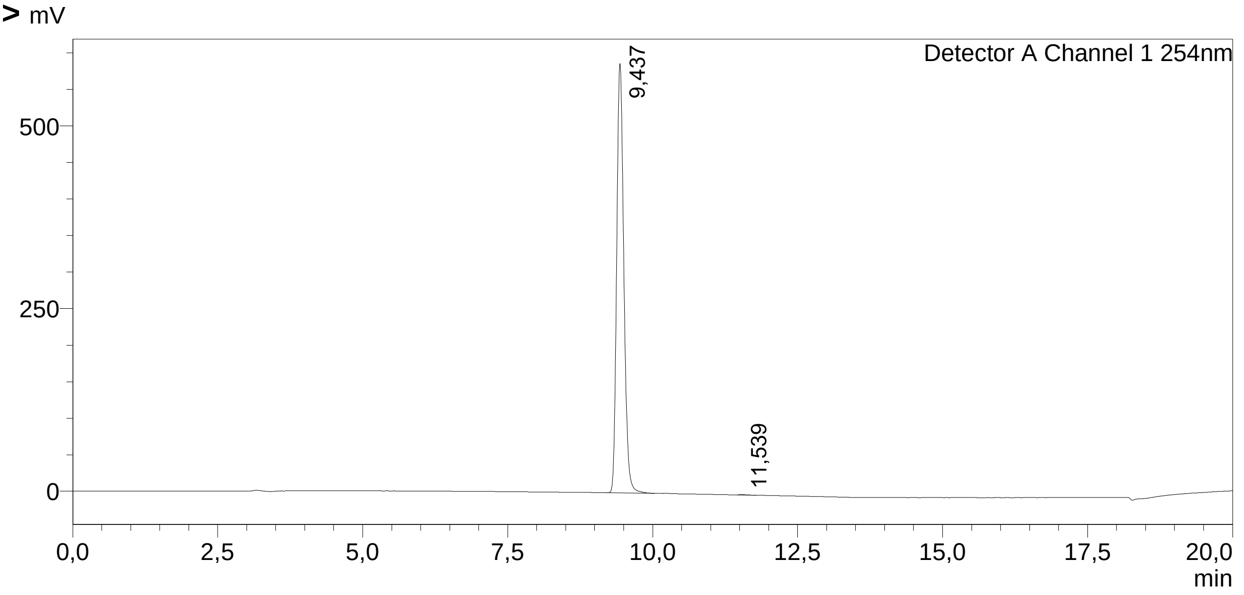

mV

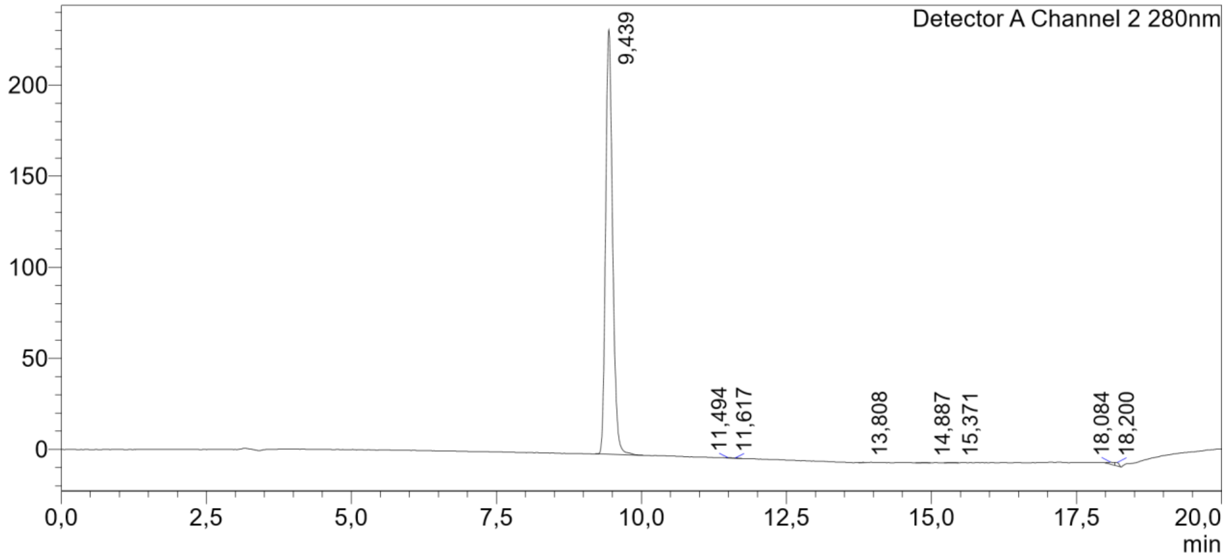

Peak Table

Detector A Channel 1 254nm

Peak#	Ret. Time	Area	Height	Conc.	Unit	Mark	Name
1	9,437	5039334	587855	99,918			
2	11,539	4123	559	0,082			
Total		5043456	588414				

Detector A Channel 2 280nm

Peak#	Ret. Time	Area	Height	Conc.	Unit	Mark	Name
1	9,439	1960536	233141	98,842			
2	11,494	1253	207	0,063			
3	11,617	1481	236	0,075		V	
4	13,808	1073	168	0,054		V	
5	14,887	1860	211	0,094			
6	15,371	1433	170	0,072			
7	18,084	8250	976	0,416			
8	18,200	7608	1821	0,384		V	
Total		1983495	236929				

Chemical biology

Cloning of the sEH-HiBiT construct and generation of the cell line HeLa$^{sEH-HiBiT}$

In order to generate the cell line HeLa$^{sEH-HiBiT}$, HeLa cells were stably transfected with the construct hsEH_aa1-aa555_Linker-HiBiT_pSB-hPGK using the Sleeping Beauty system.[2] The construct was generated using Gibson cloning[3] and encodes for human sEH (aa1-aa555) C-terminally fused to the HiBiT peptide. Expression is governed under the control of aby the constitutively active hPGK promoter.

First an intermediate construct hsEH_aa1-aa555_Linker-HiBiT_pSBtet was generated, which can be used to stably transfect cells that express hsEH_aa1-aa555_Linker-HiBiT under the control of the Doxycyclin inducible tetOn promotor. The intermediate construct was generated by inserting a hsEH_aa1-aa555_Linker-HiBiT sequence into pSBtet-bla (addgene #60510).

Thereafter, the tetOn promoter was replaced with the hPGK promoter giving the final construct hsEH_aa1-aa555_Linker-HiBiT_pSB-hPGK.

Coding DNA sequence (CDS) and protein sequence of the hsEH-Linker-HiBiT are identical for the intermediate and final construct.

CDS of (hsEH-Linker-*HiBiT*):
ATGACGCTGCGCGCGGCCGTCTTCGACCTTGACGGGGTGCTGGCGCTGCCAGCGGTGTTC
GGCGTCCTCGGCCGCACGGAGGAGGCCCTGGCGCTGCCCAGAGGACTTCTGAATGATGCT
TTCCAGAAAGGGGGACCAGAGGGTGCCACTACCCGGCTTATGAAAGGAGAGATCACACT
TTCCCAGTGGATACCACTCATGGAAGAAAACTGCAGGAAGTGCTCCGAGACCGCTAAAG
TCTGCCTCCCCAAGAATTTCTCCATAAAAGAAATCTTTGACAAGGCGATTTCAGCCAGAA
AGATCAACCGCCCCATGCTCCAGGCAGCTCTCATGCTCAGGAAGAAAGGATTCACTACTG
CCATCCTCACCAACACCTGGCTGGACGACCGTGCTGAGAGAGATGGCCTGGCCCAGCTGA
TGTGTGAGCTGAAGATGCACTTTGACTTCCTGATAGAGTCGTGTCAGGTGGGAATGGTCA
AACCTGAACCTCAGATCTACAAGTTTCTGCTGGACACCCTGAAGGCCAGCCCCAGTGAGG
TCGTTTTTTTGGATGACATCGGGGCTAATCTGAAGCCAGCCCGTGACTTGGGAATGGTCA
CCATCCTGGTCCAGGACACTGACACGGCCCTGAAAGAACTGGAGAAAGTGACCGGAATC
CAGCTTCTCAATACCCCGGCCCCTCTGCCGACCTCTTGCAATCCAAGTGACATGAGCCAT
GGGTACGTGACAGTAAAGCCCAGGGTCCGTCTGCATTTTGTGGAGCTGGGCTCCGGCCCT
GCTGTGTGCCTCTGCCATGGATTTCCCGAGAGTTGGTATTCTTGGAGGTACCAGATCCCTG
CTCTGGCCCAGGCAGGTTACCGGGTCCTAGCTATGGACATGAAAGGCTATGGAGAGTCAT
CTGCTCCTCCCGAAATAGAAGAATATTGCATGGAAGTGTTATGTAAGGAGATGGTAACCT
TCCTGGATAAACTGGGCCTCTCTCAAGCAGTGTTCATTGGCCATGACTGGGGTGGCATGC
TGGTGTGGTACATGGCTCTCTTCTACCCCGAGAGAGTGAGGGCGGTGGCCAGTTTGAATA
CTCCCTTCATACCAGCAAATCCCAACATGTCCCCTTTGGAGAGTATCAAAGCCAACCCAG
TATTTGATTACCAGCTCTACTTCCAAGAACCAGGAGTGGCTGAGGCTGAACTGGAACAGA
ACCTGAGTCGGACTTTCAAAAGCCTCTTCAGAGCAAGCGATGAGAGTGTTTTATCCATGC
ATAAAGTCTGTGAAGCGGGAGGACTTTTTGTAAATAGCCCAGAAGAGCCCAGCCTCAGC
AGGATGGTCACTGAGGAGGAAATCCAGTTCTATGTGCAGCAGTTCAAGAAGTCTGGTTTC
AGAGGTCCTCTAAACTGGTACCGAAACATGGAAAGGAACTGGAAGTGGGCTTGCAAAAG
CTTGGGACGGAAGATCCTGATTCCGGCCCTGATGGTCACGGCGGAGAAGGACTTCGTGCT
CGTTCCTCAGATGTCCCAGCACATGGAGGACTGGATTCCCCACCTGAAAAGGGGACACAT
TGAGGACTGTGGGCACTGGACACAGATGGACAAGCCAACCGAGGTGAATCAGATCCTCA
TTAAGTGGCTGGATTCTGATGCCCGGAACCCACCGGTGGTCTCAAAGATGAGCAGCGGCA
ACAGCGGCGGATCTAGCGG*AGTTTCTGGATGGCGGCTGTTCAAGAAGATCAGC*

Protein sequence (hsEH-Linker-*HiBiT*):
MTLRAAVFDLDGVLALPAVFGVLGRTEEALALPRGLLNDAFQKGGPEGATTRLMKGEITLSQ
WIPLMEENCRKCSETAKVCLPKNFSIKEIFDKAISARKINRPMLQAALMLRKKGFTTAILTNT
WLDDRAERDGLAQLMCELKMHFDFLIESCQVGMVKPEPQIYKFLLDTLKASPSEVVFLDDIG
ANLKPARDLGMVTILVQDTDTALKELEKVTGIQLLNTPAPLPTSCNPSDMSHGYVTVKPRVR
LHFVELGSGPAVCLCHGFPESWYSWRYQIPALAQAGYRVLAMDMKGYGESSAPPEIEEYCM
EVLCKEMVTFLDKLGLSQAVFIGHDWGGMLVWYMALFYPERVRAVASLNTPFIPANPNMSP
LESIKANPVFDYQLYFQEPGVAEAELEQNLSRTFKSLFRASDESVLSMHKVCEAGGLFVNSPE
EPSLSRMVTEEEIQFYVQQFKKSGFRGPLNWYRNMERNWKWACKSLGRKILIPALMVTAEK

DFVLVPQMSQHMEDWIPHLKRGHIEDCGHWTQMDKPTEVNQILIKWLDSDARNPPVVSKMS SGNSGGSSG*VSGWRLFKKIS*

Cloning details intermediate construct hsEH_aa1-aa555_Linker-HiBiT_pSBtet:

The primers used for DNA amplification were purchased from *Eurofin*s and all PCRs were performed using Q5® High-Fidelity DNA Polymerase (*New England BioLabs*) according to the manufacturer's recommendations. PCR was always followed by a digestion with DpnI (*Thermo Fisher Scientific*) at 37 °C for 1 h, inactivated at 80 °C for 20 min and a purification step using the GeneJET PCR Purification Kit (*Thermo Fisher Scientific*) according to the supplier's protocol.

The hsEH-Linker-HiBit insert was generated in two steps. In PCR 1 a dsDNA fragment encompassing the sequence coding for Linker-HiBit was amplified from a plasmid containing the CDS for Linker (SSGNSGGSSG) and HiBit (VSGWRLFKKIS) codon-optimized for *h.s.* Forward primer (5'-CCACCGGTGGTCTCAAAGATGAGCAGCGGCAACAGC-3') and reverse primer (5'-TCGATGGAAGCTTGGCCTGACAGGCCTCAGCTGATCTTCTTGAACAGCCG-3') were used to attach 21 base pair (bp) to the 5' end overlapping with the end of the CDS of hsEH and 29 bp to the 3' end introducing the TGA Stop codon and overlapping to the vector backbone of pSBtet-Bla (sequence around the SfiI site 3' of MCS). The mixture was heated at 98 °C for 1 min followed by 25 amplification cycles at 98 °C for 20 s, 66 °C for 20 s, and 72 °C for 30 s, respectively. After amplification a final step of 5 min at 72 °C was performed.

In order to generate the desired insert (hsEH_aa1-aa555_Linker-HiBiT) PCR 2 then followed a fusion PCR approach by using as template both, the product from PCR 1 and the published sEH construct of Hahn et al.[4], and the reverse primer already used in PCR 1 in combination with the forward primer (5'-TACCCTCGAAAGGCCTCTGAGGCCACCATGACGCTGCGCGC-3'). The latter introduced 27 bp to the 5' end that overlap with the sequence around the 5' SfiI site in pSBtet-Bla (addgene #60510) in order to later enable gibson assembly. The mixture was heated at 98 °C for 1 min followed by 25 amplification cycles at 98 °C for 40 s, 65 °C for 20 s, and 72 °C for 1 min 30 s, respectively. After amplification a final step of 5 min at 72 °C was performed.

In PCR 3 the entire vector backbone of pSBtet-Bla (addgene #60510) was amplified using the forward primer (5'-TGAGGCCTGTCAGGCCAAGCTTCCATCGA-3') and the reverse primer (5'-CATGGTGGCCTCAGAGGCCTTTCGAGGGTA-3'). The mixture was heated at 98 °C for 1 min followed by 25 amplification cycles at 98 °C for 20 s, 66 °C for 20 s, and 72 °C for 5 min, respectively. After amplification a final step of 8 min at 72 °C was performed.

The final construct was generated using the NEBuild® HiFi DNA Assembly Cloning Kit (*New England BioLabs*) according to the manufacturer's protocol by performing the reaction in 5 µl containing ~ 40 ng of insert (PCR 2) and ~52 ng of backbone DNA (PCR 3).

Cloning details of final construct hsEH_aa1-aa555_Linker-HiBiT_pSB-hPGK:

To generate a dsDNA fragment containing the hPGK promotor PCR 4 was performed using the pLKO.1-puro-shNM (cf. SHC002 from Sigma-Aldrich) as template with the forward primer (5'-GGTCCGCTATCTAGACGAGTAGCAGAGATCCACTTTGGCC-3') and the reverse primer (5'-GGCAAAAGAGTTGGAATTGGCCCTGGGGAGAGAGGTCGG-3'). The mixture was heated at 98 °C for 30 s followed by 25 amplification cycles at 98 °C for 10 s, 62°C for 30 s, and 72 °C for 30 s, respectively. After amplification a final step of 4 min at 72 °C was performed.
In PCR 5 the plasmid hsEH_aa1-aa555_Linker-HiBiT_pSBtet (the intermediate construct) was used as template. Using the forward primer (5'-GCCAATTCCAACTCTTTTGCCTTATACC-3') and the reverse primer (5'- ACTCGTCTAGATAGCGGACC-3') a linearized amplificate of the plasmid was produced leaving out only the section corresponding to the tet-On promoter. The mixture was heated at

98 °C for 2 min followed by 30 amplification cycles at 98 °C for 40 s, 62 °C for 20 s, and 72 °C for 5 min, respectively. After amplification a final step of 8 min at 72 °C was performed.

The final construct was generated using the NEBuilder® HiFi DNA Assembly Cloning Kit (*New England BioLabs*) according to the manufacturer's protocol by performing the reaction in 5 µl containing ~13 ng of hPGK fragment (PCR 4) and ~ 66 ng of PCR 5.

Plasmid validation:

Generated plasmids were validated by overlapping Sanger sequencing (*Microsynth Seqlab*) of the entire sequence spanning promoter and hsEH-HiBit CDS.

Stable transfection of HeLa cells using the Sleeping Beauty System:[2]

Two days prior to transfection, HeLa cells were seeded in 6-well plates at a density of 4 x 10^5 cells per well in 3 mL DMEM (1X) medium with phenol red (*Thermo Fisher Scientific, #41965-039*) supplemented with 10% Corning® Fetal Bovine Serum (*Corning*®, 35-079-CV), penicillin (100 units/ml), and streptomycin (100 µg/mL) (Gibco #15140), and 1 mM sodium pyruvate (Gibco #11360). In the following the medium is referred to as DMEMsup. On the day of transfection each well was washed with 2 mL PBS and incubated with 1 mL Opti-MEM™ medium. Transfection followed the protocol of the Lipofectamine™ 3000 reagent with slight modifications. Briefly, 500 µl Opti-MEM™ medium was mixed with 4.2 µg hsEH_aa1-aa555_Linker-HiBiT_pSB-hPGK, 0.2 µg pSB100x plasmid and 8.8 µL P3000™ reagent. In another tube, 500 µL Opti-MEM™ medium was mixed with 4 µL Lipofectamine™ 3000 reagent. Both mixtures were combined and incubated at r.t. for 10 min. The final mixture was added to one well of the 6-well plate and incubated at 37 °C and 5% CO_2 for 4 h. Then, the medium was replaced by 2 mL DMEMsup. One day after transfection, the cells were transferred to a 75 cm^2 tissue culture flask and subjected to selection medium (50 mL DMEMsup mixed with 25 µL of a 10 mg/mL blasticidin solution resulting in 5 µg/ml blasticidin). After 4 days, the newly generated cell lines were transferred to a 175 cm^2 tissue culture flask and further selected for 10 to 14 days.

sEH HiBiT Assay

$HeLa^{sEH\text{-}HiBiT}$ cells were maintained at 37°C and 5% CO_2 and split twice a week (1.8 mio cells per flask). The cells were cultivated in 175 cm² cell culture flasks (*greiner BIO-ONE*) in DMEMsup. For splitting, Gibco™ Trypsin-EDTA and Gibco™ DPBS (no calcium, no magnesium) purchased from *Thermo Fisher Scientific* were used.

In preparation for the assay, 8 mio cells were seeded into a 175 cm² cell culture flask and incubated for 24 h at 37 °C and 5% CO_2. Afterwards, cells were harvested in DMEMsup, cell density was adjusted to 4 x10^5 cells/mL, and the cells were seeded into a 384-well TC plate (Nunc™ white polystyrole, flat bottom, Cat.Nr. 1262058) using a Multidrop combi (*Thermo Fisher Scientific*) at 50 µl/well resulting in 2000 cells per well. The plate was sealed with a semipermeable AeraSeal™ film (*Sigma-Aldrich/Merck*, A9224) and cells were incubated for 24 h at 37 °C and 5% CO_2. Compound dilutions, including the positive control **2** with a final concentration of 300 nM in the assay, and dilution series of the tested compounds were prepared in DMEMsup (final DMSO concentration 5.5%) from respective compound stocks in DMSO or pure DMSO. 5 µL of the respective dilutions were added to the cells in triplicates for a final volume of 55 µL and a final DMSO concentration of 0.5%. The plate was centrifuged for 1 min at 300 rpm, then resealed with AeraSeal™ film, and the cells then incubated for 24 h at 37 °C and 5% CO_2. The same procedure was conducted for incubation times of 18 h, 3 h and 1 h. After incubation with the compound dilutions for the respective time intervals, cells were washed four times with DPBS using a Hydrospeed™ plate washer device (*Tecan*) with a remaining volume of 10 µL in each well. Cell

lysis was performed by adding 1 µL of Mammalian Lysis Buffer (*Promega*) to each well which was followed by centrifugation for 1 min at 300 rpm and incubation for 10 min at rt. Meanwhile, the Nano-Glo® substrate mix was freshly prepared from 1600 µL Nano-Glo® HiBiT Extracellular Buffer, 52 µL Nano-Glo® HiBiT Extracellular Substrate and 26 µL LgBiT Protein (all part of the Nano-Glo® HiBiT Extracellular Detection System Kit, *Promega*). Subsequently, 10 µL of the Nano-Glo® substrate mix were added to each well, followed by centrifugation for 1 min at 300 rpm. After incubation for 10 min at rt, the luminescence signal was detected using a Spark Multimode Microplate Reader (*Tecan*). The mean luminescence signal of each triplicate relative to the mean DMSO control signal was plotted against time or concentration in Prism 7.0 (*GraphPad Software, Inc.*). To determine the DC_{50} values, the normalized luminescence signals were plotted against the logarithmic compound concentration, and data analysis was performed using the nonlinear regression curve fit "log(Inhibitor) vs. Response – Variable slope (four parameters)" in Prism 7.0.

sEH-H activity assay

The *in vitro* potency of the synthesized PROTACs towards sEH-H was determined using a fluorescence-based activity assay with the fluorogenic substrate (3-phenyl-cyano(6-methoxy-2-naphthalenyl)methyl ester-2-oxiraneacetic acid) PHOME[5] according to a published protocol.[6] For the assay, human and murine full-length sEH were used which were expressed and purified as described by Lukin et al.[7] and Lillich et al.,[8] respectively.

In brief, dilution series of each tested compound were incubated with either human full-length sEH (final concentration 3 nM) or with murine full-length sEH (final concentration 10 nM) in a 96-well plate (black, flat bottom) for 30 min. As a positive control, the protein was incubated with DMSO vehicle. After incubation, an aqueous solution of PHOME (final concentration 50 µM) was added quickly to each well. The fluorescence was then measured every 60 s for 45 min using a *Tecan* Infinite F200 pro multimode plate reader (excitation: 360 nm, emission: 465 nm; bandwidth 35 nm). Inhibition [%] was plotted against the logarithmic concentration and IC_{50}-values were determined using the nonlinear regression curve fit "log(Inhibitor) vs. Response –Variable slope (four parameters)" in Prism 7.0.

solubility limit assay

The solubility in water was measured for compounds **23** and **24** in a simple assay setup based on the changing light absorption of a solution upon precipitation and light scattering of a compound. A dilution series of the compounds were prepared in DMSO and 0.5 µL of the respective stock solution was pipetted into a transparent 96-well plate with a flat bottom (in triplicates). In addition, 99.5 µL of DBPS buffer (Gibco™ Dulbecco's phosphate buffered saline (1x, pH = 7.0-7.3, Thermo Fisher Scientific) supplemented with 0.01 % aqueous Triton X100 solution) was added to each well (0.5% final DMSO concentration). For each well, the absorption was measured at 600 nm using a *Tecan* Spark Multimode plate reader. The absorption values of the compound dilutions were compared to the DMSO vehicle using Microsoft Excel and the change of absorption marked the solubility limit.

Evaluation of the linker length with MOE

The structure of FL217 (**3**) in complex with C-terminal domain of sEH (PDB code: 7P4K) was loaded into MOE software suite. Subsequently, default preparation using QuickPrep Routine was applied. Then, one of the monomers was deleted. Then, positions of protein residues outside the radius of 4.5 A around the ligand FL217 were fixed. Using the Builder tool, either the sulfonamide moiety or the N-

benzyl moiety of FL217 was subsequently changed by the alkyl chain of different length bound to N-methyl triazole, and the energy of the complex was minimized using the AMBER:EHT force field with default settings. It was observed that in both cases, the linker of three carbons is sufficient to exit the protein. The pdb files of the structures with three carbon linker are enclosed.

Crystallography

Crystallization of sEH-H-PROTACs complex

sEH-H was expressed and purified as described by Lillich et al.[8] and concentrated to ~16.7 mg/mL. The protein was cocrystallized with PROTACs **21b** and **22b** by adding them to the protein with a final concentration of 1 mM and 0.6 mM, respectively, and incubated on ice for 1 hour. Initial crystallization trials were performed using commercially available screens. A drop volume of 200 nL was equilibrated against 20 μL of reservoir solution in 96-well sitting drop SWISSCI plates. The crystallization plates were incubated at 20 °C. The crystals for sEH-H-**21b** complex were obtained in 0.2 M Ammonium acetate, 0.1 M Sodium citrate tribasic dihydrate pH = 5.6, 30% w/v Polyethylene glycol 4,000, and those for sEH-H-**22b** were obtained in 20% PEG6000, 10% ethylene glycol, 0.1 M tris pH = 7.5, 0.1 M magnesium chloride.

Data collection and processing

A single crystal for each complex was picked from the crystallization plate, treated with 25% ethylene glycol in reservoir solution as the cryoprotectant, and frozen in liquid nitrogen. The data were collected at the Swiss Light Source X06SA beamline. The temperature was maintained at 100 K during the data collection. 900 images were collected for each crystal, and the data were processed using XDS.[9] The intensities were scaled and converted into structure factors using AIMLESS.[10,11] of the CCP4 suite.[12]

Structure determination and refinement

The structures were solved by molecular replacement using the MOLREP program[13] of the CCP4 suite with a published sEH-H structure (PDB ID: 7P4K)[8,14] as the search template. The model was built manually using COOT[15], and the structure was refined by REFMAC[16,17] of the CCP4 suite. The model was validated using the wwPDB Validation System (https://validate-rcsb-2.wwpdb.org/) prior to deposition. The data collection, processing, and refinement statistics are shown in Table S1. The coordinates for sEH-H-**21b** and sEH-H-**22b** were deposited in the Protein Data Bank with PDB IDs 8S76 and 8S77, respectively.

Table S1. Data collection, processing, and refinement statistics for sEH-H-PROTACs complex

	**21b (8S76)**	**22b (8S77)**
**Data collection and reduction**		
Wavelength (Å)	1.0	1.0
Space group	P21	P21
Resolution range (Å)	47.36 - 1.48	47.26 - 1.36
Last resolution shell (Å)	1.51 - 1.48	1.38 - 1.36
Unit cell parameters		
a,b,c (Å)	47.40, 80.36, 90.22	47.28, 80.34, 89.25
α, β, γ (°)	90, 92.17, 90	90, 91.87, 90
Total number of observations	379754 (18194)	475714 (22251)
Unique reflections	111646 (5253)	141572 (6994)

Mosaicity (˚)	0.15	0.18
Multiplicity	3.4 (3.5)	3.4 (3.2)
Mean I/σ(I)	10.3 (1.6)	11.7 (1.1)
Completeness (%)	99.3 (96.3)	99.8 (57.4)
$R_{merge}{}^{b}$	0.048 (0.780)	0.038 (0.959)
$R_{meas}{}^{c}$	0.067 (1.053)	0.053 (1.332)
$R_{pim}{}^{d}$	0.045 (0.703)	0.036 (920)
**Refinement**		
Resolution range (Å)	47.36 - 1.48	47.26 - 1.36
Number of reflections used	106133	134595
Number of Free R flagged reflections	5464	6949
$R_{cryst}{}^{e}$	0.16618	0.19429
$R_{free}{}^{f}$	0.19918	0.21493
rmsd Bond length (Å)	0.011	0.010
rmsd Bond angle (˚)	1.834	1.838
Ramachandran plot, residues in		
Most favored region (%)	98	96
Additionally allowed region (%)	2	3
Average B-factor (Å^2)	25.515	25.476

[a]Values for the last resolution shell are in parentheses.

$^{b}R_{merge} = \Sigma_{hkl}\Sigma_i|I_i(hkl)-\langle I(hkl)\rangle| / \Sigma_{hkl}\Sigma_i I_i(hkl)$, where $I(hkl)$ is the intensity of reflection hkl

$^{c}R_{meas} = \Sigma_{hkl}\{N(hkl)/[N(hkl)-1]\}^{1/2}\Sigma_i|I_i(hkl)-\langle I(hkl)\rangle| / \Sigma_{hkl}\Sigma_i I_i(hkl)$

$^{d}R_{pim} = \Sigma_{hkl}\{1/[N(hkl)-1]\}^{1/2}\Sigma_i|I_i(hkl)-\langle I(hkl)\rangle| / \Sigma_{hkl}\Sigma_i I_i(hkl)$

$^{e}R_{cryst} = \Sigma_{hkl}\ ||F_{obs}|-|F_{calc}|| / \Sigma_{hkl}|F_{obs}|$

$^{f}R_{free}$ is the cross-validation R-factor computed for the test set of unique reflections.

CellTiter-Glo Assay

For the viability assay, HepG2 cells were maintained at 5% CO_2 and 37°C in Dulbecco's modified Eagle's medium (DMEM) high-glucose with phenol red (Gibco #41965) supplemented with 20% fetal bovine serum (FBS; capricon), 1 mM sodium pyruvate (Gibco #11360), penicillin (100 units/ml), and streptomycin (100 µg/mL) (Gibco #15140). For the last passage before the cells were used in the assay, the tissue culture (TC) flask was coated with collagen G. Therefore, PBS supplemented with 0.01 mg/mL collagen G (Merck, #L7213) was incubated for 30 min at 37°C in the new TC flask and then immediately replaced with new growth medium. After 3-4 days, cells were harvested using Trypsin and recovered in white DMEM high-glucose medium (Gibco #31053) supplemented with 10% FBS, 100 units/mL penicillin, and 100 µg/mL streptomycin as well as 2 mM L-Glutamin (Gibco). After passage through a 40 µm cell strainer (pluriselect #43-50040), cell density was adjusted to 100,000 cells/mL, and 30 µL/well, (equivalent to 3000 cells/well) were seeded into 96-well half area white PS (polystyrol) flat bottom TC plates (Greiner bio-One, #675083). PROTACs **23** and **24** were prepared as dilution series in medium with 2% DMSO, and 10 µL were added per well resulting in the indicated compound concentrations and 0.5% DMSO during treatment. Treatment with 50 µM Paclitaxel was conducted in parallel as an assay performance control. After 72 h, CellTiter-Glo® reagent (Promega G7570) was applied at 20 µL/well and protected from light evolved for 30 min at rt. Luminescence was then recorded using the standard attenuation protocol on a Tecan SPARK.

Mean of three technical replicates (N = 3) per concentration was normalized using wells with medium alone and wells with cells treated with only DMSO as 0 and 100% cell viability control, respectively; controls N = 8 each. Live rates were calculated from two biological replicates (n = 2) and are reported as mean ± SEM.

Metabolic stability in mouse and rat liver microsomes

For PROTACs **23** and **24**, the microsomal stability was determined. The solubilized test compounds (5 µL, final concentration 10 µM) were pre-incubated at 37°C in 432 µL of phosphate buffer (0.1 M, pH = 7.4) together with 50 µL NADPH regenerating system (30 mM glucose-6-phosphate, 4 U/mL glucose-6-phosphate dehydrogenase, 10 mM NADP, 30 mM $MgCl_2$). After 5 min, the reaction was started by the addition of 13 µL of microsome mix from the liver of Sprague−Dawley rats (*Invitrogen*; 20 mg protein/mL in 0.1 M phosphate buffer) in a shaking water bath at 37°C. The reaction was stopped by adding 500 µL of ice-cold methanol at 0, 15, 30 and 60 min. The samples were centrifuged at 5000 g for 5 min at 4°C. The supernatants were analyzed and test compounds were quantified by HPLC: The composition of the mobile phase is adapted to the test compound in a range of MeOH 40-90% and water (0.1% formic acid) 10-60%; flow-rate: 1 mL/min; stationary phase: Purospher® STAR, RP18, 5 µm, 125×4, precolumn: Purospher® STAR, RP18, 5 µm, 4×4; detection wavelength: 254 nm and 280 nm; injection volume: 50 µL. Control samples were performed to check the test compounds´ stability in the reaction mixture: first control was without NADPH, which is needed for the enzymatic activity of the microsomes, sond control was with inactivated microsomes (incubated for 20 min at 90°C), and third control was without test compound (to determine the baseline). The amounts of the test compounds were quantified by an external calibration curve. Data are expressed as the mean ± SEM remaining compound from three independent experiments.

Target engagement assay and proteomics

Cell cultivation

Stably transfected soluble epoxide hydrolase overexpressing HeLa cells were cultured in DMEM (high glucose) with 10% FCS, 100 $U \cdot mL^{-1}$ penicillin, 100 $\mu g \cdot mL^{-1}$ streptomycin and 1 mM sodium pyruvate in 60.1 cm^2 dishes in a humidified incubator at 37°C and 5% CO_2. For passaging, cells were detached from dishes using trypsin and $1 \cdot 10^6$ cells were transferred to new dishes every three to four days.

For the sEH activity assay and the targeted proteomics analysis, approximately $2.5 \cdot 10^6$ cells were incubated with 3 µM of the test compounds PROTACs (0.5 % DMSO) and harvested on ice by scraping.

Measurement of sEH inhibition

Cell pellets were homogenized in PBS with an ultrasonic tip and pooled. Homogenates (0.5-1.3 mg protein/mL, determined by bicinchoninic acid assay) were used for sEH activity assay. Activity assay was started by addition of a mixture of the substrates (70 µM 14(15)-, 40 µM 11(12)- and 20 µM 8(9)-EpETrE) and the mixture was incubated for 30 minutes at 37°C. The assay was stopped by addition of ice-cold ethyl acetate, and oxylipins were extracted twice by liquid-liquid extraction. The combined ethyl acetate phases were collected, evaporated to dryness and the residue was reconstituted in methanol. Products were quantified using targeted LC-MS/MS measurement of 14,15- (*m/z* 337.2 → 207.1), 11,12- (*m/z* 337.2 → 167.1) and 8,9 DiHETrE (337.2 → 127.1) using 17,18-DiHETE (*m/z* 335.2 → 247.2) as internal standards.[18,19]

Targeted LC-MS/MS based proteomics:

Sample preparation was carried out as described.[20,21] In brief, cell pellets were re-dissolved in 5% *w/v* sodium deoxycholate (SDC) containing 1% protease inhibitor mix, sonicated and centrifuged (15,000 x *g*, 4°C, 10 min). A solution containing 500 µg total protein were used for further sample preparation. Proteins were precipitated and washed with ice-cold acetone. Dry pellets were re-dissolved in 6 M urea to a final concentration of 5 mg·mL^{-1} and incubated with 200 mM dithiothreitol (DTT, in 50 mM NH_4HCO_3) for 1 h followed by an 1 h incubation with 200 mM iodoacetamide (IAA, in 50 mM NH_4HCO_3) for alkylation of the resulting free sulfhydryl groups. Unreacted IAA was consumed by another 1 h incubation with DTT.

Tryptic digestion using > 6.000 U·g^{-1} trypsin from porcine pancreas (Serva Electrophoresis GmbH, Heidelberg, Germany; product number 37286.03) was carried out for 15h at 37°C (pH ≈ 7.8). Reaction was stopped by addition of concentrated HAc to reduce the pH to 3-4 and to precipitate SDC. Following centrifugation, peptides were purified by SPE using Strata-X 33 µm polymeric reversed phase material, Phenomenex LTD, Aschaffenburg, Germany). Residues were re-dissolved in 15% ACN (0.1% HAc) and centrifuged (15,000 x *g*, 4°C, 10 min). Clear supernatants were analyzed by targeted LC-MS/MS proteomics analysis and sEH was quantified based on the quantifier peptide TEEALALPR^{2+} (transition y_7^+ as quantifier and b_2^+/ y_5^+ as qualifier) and the qualifier peptide WLDSDAR^{2+} (b_2^+ and y_5^+) using the heavy labeled peptides (TEEALALPR^{2+} and WLDSDAR^{2+}, lys: U-^{13} C_6; U-^{15} N_2; arg: U-^{13} C_6; U-^{15} N_4) all obtained from JPT Peptides Berlin, Germany as internal standards.[18–21]

Experiments in primary mouse hepatocytes

Isolation and culture of murine hepatocytes

Primary murine hepatocytes were isolated as previously described[22], using a modified two-step isolation method. Briefly, after cervical dislocation, disinfection, and laparotomy of 6- to 8-week-old C57BL6/N mice, a 24 G Introcan™ safety catheter (*Braun*) was inserted into the inferior vena cava. The portal vein was then dissected, and retrograde liver perfusion was performed using pre-warmed Hank's balanced salt solution (without Ca^{2+} and Mg^{2+}) supplemented with 0.5 mmol/L EDTA. After 3 min, the perfusion medium was switched to Hams F12 Medium (*Thermo Fisher Scientific*) containing Liberase™ TM (25 µg/mL, *Merck*) for additional 5 min. The pH of both solutions was stabilized with carbogen delivered through a glass frit during perfusion. The isolated cells were seeded at a density of 2.5×10^5 cells/mL on collagen-coated plates (50 µg/mL) in Williams' Medium E (*Thermo Fisher Scientific*) supplemented with 8% heat-inactivated fetal calf serum, 2 mmol/L L-glutamine, 100 U/mL penicillin, and 100 µg/mL streptomycin. After 4 h, the medium was replaced with FCS-free medium. Treatments with 0.5% DMSO and PROTACs **23** or **24** or the respective control compounds **31** and **32** (0.3 µmol/L in 0.5% DMSO) were initiated 24 h post-isolation and continued for 18 h.

Immunoblotting

For immunoblotting, cells were lysed in Triton X-100 lysis buffer containing 50 mmol/L Tris-HCl (pH 7.5), 150 mmol/L NaCl, 10 mmol/L Sodium Phosphate Buffer, 20 mmol/L NaF, and 1% Triton X-100, supplemented with protease and phosphatase inhibitors. Detergent soluble proteins were resuspended in SDS-PAGE sample buffer, separated by SDS-PAGE, and transferred to membranes for Western blotting. Membranes were blocked with Rotiblock (*Roth*) for 2 h, followed by overnight incubation at 4 °C with primary antibodies against soluble epoxide hydrolase (custom-made, #4536.2, *ProteinTech*) and β-actin (*Merck*). After washing, horseradish peroxidase-conjugated sondary antibodies were applied for 2 h, and protein bands were visualized using Lumi-Light Plus (*Roche*). Luminescence was captured using the Fusion FX7 imaging system (*Vilber-Lourmat*).

Experiments in human M1 macrophages

Fluorescence microscopy experiments in human M1 macrophages

Materials used in these experiments:

Reagent	Supplier
**Dextran from *Leuconostoc* spp.**	Sigma-Aldrich Chemie GmbH, Taufkirchen, Germany
**Recombinant human Granulocyte-Macrophage Colony-Stimulating Factor (GM-CSF)**	PeproTech, Rocky Hill, NJ, USA
**Recombinant human type II interferon (IFNg)**	PeproTech, Rocky Hill, NJ, USA
**Roti®-Histofix, 4 %**	Carl Roth GmbH, Karlsruhe, Germany
**Triton X®-100**	Merck KGaA, Darmstadt, Germany
**Fetal Bovine Serum (FCS) Superior**	Biochrom AG, Berlin, Germany
**Human serum (HS)**	Deutsches Rotes Kreuz, Blutspendedienst Baden-Württemberg/Hessen, Frankfurt, Germany
**Penicillin-Streptomycin (P:10,000 U/ml, S: 10 mg/ml)**	PAN-Biotech GmbH, Aidenbach, Germany
**Roswell Park Memorial Institute (RPMI)-1640**	PAN-Biotech GmbH, Aidenbach, Germany
**BSA in TBS-T (1 %, 5 %; BSA-T)**	1 %/5 % BSA, in TBS-T

**RPMI-1640 (basal RPMI)**	100 U/ml penicillin, 100 µg/ml streptomycin, in RPMI- 1640
**RPMI-1640 (RPMI-HS)**	2.5 % HS, 100 U/ml penicillin, 100 µg/ml streptomycin, in RPMI-1640
**ibiTreat µ-Slide 8 well**	Ibidi GmbH, Planegg/Martinsried, Germany

Isolation of primary monocytes

Primary human leukocytes were freshly isolated from buffy coats obtained from human peripheral blood provided by the Deutsches Rotes Kreuz (Blood Donation Service Baden-Württemberg/Hessen, Institute for Transfusion Medicine and Immunohematology, Frankfurt, Germany). The peripheral blood was diluted 1:1 with PBS at r.t. A volume of 30 mL of this diluted blood was mixed with 10 mL of dextran solution to facilitate erythrocyte sedimentation over 30 min at r.t. The leukocyte-rich supernatant (upper layer) was carefully collected and layered onto 10 mL of lymphocyte separation medium (LSM), followed by low-density centrifugation (800 g, 10 min, r.t., without brake) to separate the leukocytes.

For monocyte isolation, the peripheral blood mononuclear cells (PBMCs) that sedimented on top of the separation medium were collected, washed twice with ice-cold PBS, and once with RPMI basal medium. The cells were centrifuged at 300 g for 10 min at 4 °C, and the resulting cell pellet was resuspended in 50 mL of cold RPMI basal medium. The cell suspension was then plated and incubated at 37 °C for at least 1 h. Non-adherent lymphocytes were removed by washing the monocytes twice with warm RPMI basal medium, after which the monocytes were overlaid with RPMI + 2,5% Human Serum medium.

Differentiation of primary monocytes into macrophages

Primary monocytes, isolated as described above, were differentiated into M1 macrophages over a period of seven days. To achieve this, the monocytes were treated with 10 ng/mL of GM-CSF for M1 differentiation in RPMI-HS medium for 48 h. After a medium change, the cells were incubated for an additional 72 h with GM-CSF. The medium was then refreshed, and the cells were incubated for another 48 h with GM-CSF in combination with 10 ng/mL of IFNγ. After differentiation, the monocyte-derived macrophages were used directly.

Immunofluorescence staining in M1 macrophages

Primary M1 macrophages (1×10^5) were seeded into 8-well µ-slides and cultured for 24 h. Cells were then treated with 300 nM of **23** or **31** for 18 h. Following treatment, the cells were washed twice with PBS, fixed in 4% formaldehyde, and permeabilized with 0.2% Triton X-100. Non-specific binding sites were blocked by incubating the cells with 0.2% BSA for 30 min. The cells were subsequently incubated with a rabbit anti-human sEH antibody (dilution 1:400) for 2 h. After three washes with PBS, the cells were incubated with an Alexa Fluor 633-conjugated secondary antibody (goat anti-rabbit, dilution 1:400) for 1 h. Nuclei were stained with Hoechst 33342 (1 µg/mL). Fluorescence from sEH staining

(λ_{ex} = 590-650 nm, λ_{em} = 662-738 nm), and nuclei (λ_{ex} = 325-375 nm, λ_{em} = 435-485 nm) was visualized using a Leica DMI6000B fluorescence microscope at 63x objective.

Experiments in precision-cut lung slices

The lungs were carefully inflated with a warm 2% agarose-DMEM solution. Subsequently, an experienced pulmopathologist conducted a macroscopic evaluation of the lung and cut it into sections before we drilled 8 mm punches. Using a Krumdieck tissue slicer (Alabama Research and Development, Munford, AL, USA), the punches were cut in cold EBSS into thin slices approximately 300 µm thick precision-cut lung slices (PCLS). The PCLS were thoroughly washed overnight and then cultured in DMEM (two slices per 500 µl) under standard immersion culture conditions (37 °C, 5% CO2, and 100% humidity).

PCLS were treated with/without 300 nM of **23** or **31** in DMEM. After 18h incubation, PCLS were snapfrozen and stored at -80 °C.

Ethics

The utilization of human lung tissue obtained from patients was authorized by the Ethics Committee of Hannover Medical School (Hannover, Germany) in accordance with "The Code of Ethics of the World Medical Association" (revised on April 22, 2015, approval number 2701–2015). Written informed consent was obtained from all patients for the use of their lung tissue in research.

Western Blots

Proteins were extracted using selfmade RIPA buffer (20 mM Tris-HCl, 150 mM NaCl, 1% NP-40, 1% sodium deoxycholate, 0.1% SDS). Protein samples were separated by gel electrophoresis on 10% Mini-PROTEAN® TGX™ Precast Gels (Bio-Rad, #4561033EDU) and subsequently transferred to polyvinylidene difluoride (PVDF) membranes by overnight electroblotting.

The membranes were blocked for 1 hour at room temperature with Tris-buffered saline containing 1% Tween-20 (TBST) and 5% milk. Primary antibody incubation was performed overnight at 4°C in TBST with 5% milk using the following antibodies: rabbit anti-EPHX2 (10833-1-AP, Proteintech) at 1:500 dilution, and mouse anti-GAPDH (ab8245, Abcam) at 1:30,000 dilution.

After three washes with TBST, membranes were incubated with secondary antibodies diluted in TBST with 5% milk: anti-rabbit HRP (#7074, Cell Signaling Technology) and anti-mouse HRP (#7076, Cell Signaling Technology), both at 1:10,000 dilution. Precision Streptavidin-HRP was also used at a 1:10,000 dilution. Following another set of three washes with TBST, immunoreactive bands were visualized using the WesternSure PREMIUM Chemiluminescent Substrate (LI-COR GmbH, #926-95000).

The chemiluminescent signals were detected using the Odyssey XF imager and analyzed with the LI-COR acquisition software.

For proteome profiling, human lung tissue samples were homogenized with ReadyPrep™ Mini Grinders (BIO-RAD) in urea lysis buffer (8 M urea, 20 mM HEPES, pH 8.0, 1 mM sodium orthovanadate, 2.5 mM sodium pyrophosphate, 1 mM beta-glycerophosphate). Protein concentrations of the lysates were determined using the 660 nm assay kit (Thermo Fisher Scientific) according to the manufacturer's

instructions. 25 µg of protein per sample were reduced with DTT (10 mM for 1 h at 37 °C), alkylated with iodoacetamide (25 mM for 15 min at 37 °C in the dark) and digested using Lys-C (Wako/Fujifilm) for 2 h at 37 °C in an enzyme-to-substrate ratio of 1:50 (w/w). After dilution with 20 mM HEPES (pH 8.0) to a concentration of 2 M urea, digestion was continued overnight with trypsin (Promega) at 37 °C and 1:50 (w/w) enzyme-to-substrate ratio. The peptide mixtures were acidified, purified using C18 spin tips (Havard) and dried by vacuum centrifugation. Afterward, the peptide samples were dissolved in 0.1 % formic acid and peptide concentrations were determined using a fluorometric peptide assay (Thermo Fisher Scientific). The peptide samples were analyzed by LC-MS/MS on a Vanquish Neo UHPLC system (Thermo Fisher Scientific) coupled online to an Orbitrap Astral mass spectrometer (Thermo Fisher Scientific) in a data-independent acquisition scheme (DIA). 400 ng of peptides from each sample were concentrated and desalted on a PepMap Neo trap cartridge (Thermo Fisher Scientific, particle size 100 Å, inner diameter 300 µm, length 5 mm), followed by separation on a 50 cm µPAC C18 analytical column (Thermo Fisher Scientific) using a 30 min method (30 min linear gradient) of 1% to 36% acetonitrile in 0.1% formic acid at a flow rate of 250 nl/min. Precursor ion survey scans were acquired using the Orbitrap mass analyzer with the following parameters: resolution 240,000, scan range *m/z* 380-980, automatic gain control (AGC) target 5x10^6, maximum injection time 10 ms, RF lens setting 40%. For fragment ion scans using the Astral mass analyzer, precursor ions were isolated for collision-induced dissociation (HCD) through each survey scan with an isolation window of *m/z* 2, resulting in 299 scan events. The normalized HCD collision energy was set to 25% and for fragment ion analysis the AGC target was 5x10^4 at a maximum injection time of 3 ms.

Raw DIA data were analyzed using Proteome Discoverer (version 3.1.1.93, Thermo Fisher Scientific). Spectra were searched against the Uniprot human reference proteome and 245 frequently observed contaminants using the CHIMERYS search algorithm. The mass tolerance for fragment ions was set to 10 ppm. Oxidation of methionine was considered as dynamic modification while carbamidomethylation of cysteine was defined as a fixed modification. The peptide length was defined to be between seven to 30 amino acids with one allowed missed cleavage site. One to four charges per peptide were allowed. At both peptide and protein level, the false discovery rate (FDR) was set at 1%. Further data processing was done using R studio (version 2024.09.1). First, contaminants were removed. To control for equal sample loading, intensities from each LC-MSMS run were normalized on the median of the summed-up intensities from each sample (Plubell et al., 2017, https://doi.org/10.1074/mcp.M116.065524). Finally, data were filtered for proteins found in at least 70% of the samples. The remaining missing values were imputed using the DreamAI algorithm in R.[23]

References

(1) Wang, Y.; Morisseau, C.; Takamura, A.; Wan, D.; Li, D.; Sidoli, S.; Yang, J.; Wolan, D. W.; Hammock, B. D.; Kitamura, S. PROTAC-Mediated Selective Degradation of Cytosolic Soluble Epoxide Hydrolase Enhances ER Stress Reduction. *ACS Chem. Biol.* **2023**, *18* (4), 884–896. DOI: 10.1021/acschembio.3c00017.

(2) Kowarz, E.; Löscher, D.; Marschalek, R. Optimized Sleeping Beauty transposons rapidly generate stable transgenic cell lines. *Biotechnol. J.* **2015**, *10* (4), 647–653. DOI: 10.1002/biot.201400821.

(3) Gibson, D. G.; Young, L.; Chuang, R.-Y.; Venter, J. C.; Hutchison, C. A.; Smith, H. O. Enzymatic assembly of DNA molecules up to several hundred kilobases. *Nat. Methods* **2009**, *6* (5), 343–345. DOI: 10.1038/NMETH.1318.

(4) Hahn, S.; Achenbach, J.; Buscató, E.; Klingler, F.-M.; Schroeder, M.; Meirer, K.; Hieke, M.; Heering, J.; Barbosa-Sicard, E.; Loehr, F.; Fleming, I.; Doetsch, V.; Schubert-Zsilavecz, M.; Steinhilber, D.; Proschak, E. Complementary screening techniques yielded fragments that inhibit the phosphatase activity of soluble epoxide hydrolase. *ChemMedChem* **2011**, *6* (12), 2146–2149. DOI: 10.1002/cmdc.201100433.

(5) Wolf, N. M.; Morisseau, C.; Jones, P. D.; Hock, B.; Hammock, B. D. Development of a high-throughput screen for soluble epoxide hydrolase inhibition. *Anal. Biochem.* **2006**, *355* (1), 71–80. DOI: 10.1016/j.ab.2006.04.045.

(6) Brunst, S.; Schönfeld, J.; Breunig, P.; Burgers, L. D.; DeMeglio, M.; Ehrler, J. H. M.; Lillich, F. F.; Weizel, L.; Hefendehl, J. K.; Fürst, R.; Proschak, E.; Hiesinger, K. Designing a Small Fluorescent Inhibitor to Investigate Soluble Epoxide Hydrolase Engagement in Living Cells. *ACS Med. Chem. Lett.* **2022**, *13* (7), 1062–1067. DOI: 10.1021/acsmedchemlett.2c00073.

(7) Lukin, A.; Kramer, J.; Hartmann, M.; Weizel, L.; Hernandez-Olmos, V.; Falahati, K.; Burghardt, I.; Kalinchenkova, N.; Bagnyukova, D.; Zhurilo, N.; Rautio, J.; Forsberg, M.; Ihalainen, J.; Auriola, S.; Leppänen, J.; Konstantinov, I.; Pogoryelov, D.; Proschak, E.; Dar'in, D.; Krasavin, M. Discovery of polar spirocyclic orally bioavailable urea inhibitors of soluble epoxide hydrolase. *Bioorg. Chem.* **2018**, *80*, 655–667. DOI: 10.1016/j.bioorg.2018.07.014.

(8) Lillich, F. F.; Willems, S.; Ni, X.; Kilu, W.; Borkowsky, C.; Brodsky, M.; Kramer, J. S.; Brunst, S.; Hernandez-Olmos, V.; Heering, J.; Schierle, S.; Kestner, R.-I.; Mayser, F. M.; Helmstädter, M.; Göbel, T.; Weizel, L.; Namgaladze, D.; Kaiser, A.; Steinhilber, D.; Pfeilschifter, W.; Kahnt, A. S.; Proschak, A.; Chaikuad, A.; Knapp, S.; Merk, D.; Proschak, E. Structure-Based Design of Dual Partial Peroxisome Proliferator-Activated Receptor γ Agonists/Soluble Epoxide Hydrolase Inhibitors. *J. Med. Chem.* **2021**, *64* (23), 17259–17276. DOI: 10.1021/acs.jmedchem.1c01331.

(9) Kabsch, W. XDS. *Acta Crystallogr. D. Biol. Crystallogr.* **2010**, *66* (Pt 2), 125–132. DOI: 10.1107/S0907444909047337.

(10) Evans, P. Scaling and assessment of data quality. *Acta Crystallogr. D. Biol. Crystallogr.* **2006**, *62* (Pt 1), 72–82. DOI: 10.1107/S0907444905036693.

(11) Evans, P. R. An introduction to data reduction: space-group determination, scaling and intensity statistics. *Acta Crystallogr. D. Biol. Crystallogr.* **2011**, *67* (Pt 4), 282–292. DOI: 10.1107/S090744491003982X.

(12) Agirre, J.; Atanasova, M.; Bagdonas, H.; Ballard, C. B.; Baslé, A.; Beilsten-Edmands, J.; Borges, R. J.; Brown, D. G.; Burgos-Mármol, J. J.; Berrisford, J. M.; Bond, P. S.; Caballero, I.; Catapano, L.; Chojnowski, G.; Cook, A. G.; Cowtan, K. D.; Croll, T. I.; Debreczeni, J. É.; Devenish, N. E.; Dodson, E. J.; Drevon, T. R.; Emsley, P.; Evans, G.; Evans, P. R.; Fando, M.; Foadi, J.; Fuentes-Montero, L.; Garman, E. F.; Gerstel, M.; Gildea, R. J.; Hatti, K.; Hekkelman, M. L.; Heuser, P.; Hoh, S. W.; Hough, M. A.; Jenkins, H. T.; Jiménez, E.; Joosten, R. P.; Keegan, R. M.; Keep, N.; Krissinel, E. B.; Kolenko, P.; Kovalevskiy, O.; Lamzin, V. S.; Lawson, D. M.; Lebedev, A. A.; Leslie, A. G. W.; Lohkamp, B.; Long, F.; Malý, M.; McCoy, A. J.; McNicholas, S. J.; Medina, A.; Millán, C.; Murray, J. W.; Murshudov, G. N.; Nicholls, R. A.; Noble, M. E. M.; Oeffner, R.; Pannu, N. S.; Parkhurst, J.

M.; Pearce, N.; Pereira, J.; Perrakis, A.; Powell, H. R.; Read, R. J.; Rigden, D. J.; Rochira, W.; Sammito, M.; Sánchez Rodríguez, F.; Sheldrick, G. M.; Shelley, K. L.; Simkovic, F.; Simpkin, A. J.; Skubak, P.; Sobolev, E.; Steiner, R. A.; Stevenson, K.; Tews, I.; Thomas, J. M. H.; Thorn, A.; Valls, J. T.; Uski, V.; Usón, I.; Vagin, A.; Velankar, S.; Vollmar, M.; Walden, H.; Waterman, D.; Wilson, K. S.; Winn, M. D.; Winter, G.; Wojdyr, M.; Yamashita, K. The CCP4 suite: integrative software for macromolecular crystallography. *Acta Crystallogr. D. Struct. Biol.* **2023**, *79* (Pt 6), 449–461. DOI: 10.1107/S2059798323003595.

(13) Vagin, A.; Teplyakov, A. MOLREP : an Automated Program for Molecular Replacement. *J. Appl. Crystallogr.* **1997**, *30* (6), 1022–1025. DOI: 10.1107/S0021889897006766.

(14) Ni, X.; Kramer, J. S.; Lillich, F.; Proschak, E.; Chaikuad, A.; Knapp, S. *Soluble epoxide hydrolase in complex with FL217*, 2022. DOI: 10.2210/pdb7P4K/pdb.

(15) Emsley, P.; Lohkamp, B.; Scott, W. G.; Cowtan, K. Features and development of Coot. *Acta Crystallogr. D. Biol. Crystallogr.* **2010**, *66* (Pt 4), 486–501. DOI: 10.1107/S0907444910007493.

(16) Murshudov, G. N.; Skubák, P.; Lebedev, A. A.; Pannu, N. S.; Steiner, R. A.; Nicholls, R. A.; Winn, M. D.; Long, F.; Vagin, A. A. REFMAC5 for the refinement of macromolecular crystal structures. *Acta Crystallogr. D. Biol. Crystallogr.* **2011**, *67* (Pt 4), 355–367. DOI: 10.1107/s0907444911001314.

(17) Kovalevskiy, O.; Nicholls, R. A.; Long, F.; Carlon, A.; Murshudov, G. N. Overview of refinement procedures within REFMAC5: utilizing data from different sources. *Acta Crystallogr. D. Struct. Biol.* **2018**, *74* (Pt 3), 215–227. DOI: 10.1107/S2059798318000979.

(18) Kampschulte, N.; Alasmer, A.; Empl, M. T.; Krohn, M.; Steinberg, P.; Schebb, N. H. Dietary Polyphenols Inhibit the Cytochrome P450 Monooxygenase Branch of the Arachidonic Acid Cascade with Remarkable Structure-Dependent Selectivity and Potency. *Journal of agricultural and food chemistry* **2020**, *68* (34), 9235–9244. DOI: 10.1021/acs.jafc.0c04690.

(19) Kampschulte, N.; Berking, T.; Çelik, I. E.; Kirsch, S. F.; Schebb, N. H. Inhibition of cytochrome P450 monooxygenase-catalyzed oxylipin formation by flavonoids: Evaluation of structure-activity relationship towards CYP4F2-selective inhibitors. *Eur. J. Med. Chem.* **2022**, *238*, 114332. DOI: 10.1016/j.ejmech.2022.114332.

(20) Hartung, N. M.; Mainka, M.; Pfaff, R.; Kuhn, M.; Biernacki, S.; Zinnert, L.; Schebb, N. H. Development of a quantitative proteomics approach for cyclooxygenases and lipoxygenases in parallel to quantitative oxylipin analysis allowing the comprehensive investigation of the arachidonic acid cascade. *Analytical and bioanalytical chemistry* **2023**, *415* (5), 913–933. DOI: 10.1007/s00216-022-04489-3.

(21) Hartung, N. M.; Ostermann, A. I.; Immenschuh, S.; Schebb, N. H. Combined Targeted Proteomics and Oxylipin Metabolomics for Monitoring of the COX-2 Pathway. *Proteomics* **2021**, *21* (3-4), e1900058. DOI: 10.1002/pmic.201900058.

(22) Charni-Natan, M.; Goldstein, I. Protocol for Primary Mouse Hepatocyte Isolation. *STAR Protoc.* **2020**, *1* (2), 100086. DOI: 10.1016/j.xpro.2020.100086.

(23) Ma, W.; Kim, S.; Chowdhury, S.; Li, Z.; Yang, M.; Yoo, S.; Petralia, F.; Jacobsen, J.; Li, J. J.; Ge, X.; Li, K.; Yu, T.; Calinawan, A. P.; Edwards, N.; Payne, S. H.; Boutros, P. C.; Rodriguez, H.; Stolovitzky, G.; Zhu, J.; Kang, J.; Fenyo, D.; Saez-Rodriguez, J.; Wang, P. *DreamAI: algorithm for the imputation of proteomics data*, 2020. DOI: 10.1101/2020.07.21.214205.
